# Establishment of a *Macaca fascicularis* gut microbiome gene catalog and comparison with the human, pig, and mouse gut microbiomes

**DOI:** 10.1093/gigascience/giy100

**Published:** 2018-08-18

**Authors:** Xiaoping Li, Suisha Liang, Zhongkui Xia, Jing Qu, Huan Liu, Chuan Liu, Huanming Yang, Jian Wang, Lise Madsen, Yong Hou, Junhua Li, Huijue Jia, Karsten Kristiansen, Liang Xiao

**Affiliations:** 1 BGI-Shenzhen, Shenzhen 518083, China; 2 China National GeneBank, BGI-Shenzhen, Shenzhen 518120, China; 3 Shenzhen Key Laboratory of Human Commensal Microorganisms and Health Research, BGI-Shenzhen, Shenzhen 518083, China; 4 James D. Watson Institute of Genome Sciences, Hangzhou 310058, China; 5 School of Bioscience and Biotechnology, South China University of Technology, Guangzhou, 510006, China; 6 BGI Innovation College of QingDao University, Qingdao, 266071, China; 7 Laboratory of Genomics and Molecular Biomedicine, Department of Biology, University of Copenhagen, 2100 Copenhagen Ø, Denmark; 8 Institute of Marine Research (IMR), Postboks 1870, Nordnes, N-5817, Bergen, Norway; 9 BGI-Qingdao, BGI-Shenzhen, Qingdao,266555 China

**Keywords:** *Macaca fascicularis*, gut microbiota gene catalog, gut microbiome, core genera, high-fat/low-fiber diet, low-fat/high-fiber diet, metagenomics

## Abstract

*Macaca fascicularis*, the cynomolgus macaque, is a widely used model in biomedical research and drug development as its genetics and physiology are close to those of humans. Detailed information on the cynomolgus macaque gut microbiota, the functional interplay between the gut microbiota and host physiology, and possible similarities to humans and other mammalians is very limited. The aim of this study was to construct the first cynomolgus macaque gut microbial gene catalog and compare this catalog to the human, pig, and mouse gut microbial gene catalogs. We performed metagenomic sequencing on fecal samples from 20 cynomolgus macaques and identified 1.9 million non-redundant bacterial genes of which 39.49% and 25.45% are present in the human and pig gut bacterial gene catalogs, respectively, whereas only 0.6% of the genes are present in the mouse gut bacterial gene catalog. By contrast, at the functional levels, more than 76% Kyoto Encyclopedia of Genes and Genomes orthologies are shared between the gut microbiota of all four mammalians. Thirty-two highly abundant bacterial genera could be defined as core genera of these mammalians. We demonstrated significant differences in the composition and functional potential of the gut microbiota as well as in the distribution of predicted bacterial phage sequences in cynomolgus macaques fed either a low-fat/high-fiber diet or a high-fat/low-fiber diet. Interestingly, the gut microbiota of cynomolgus macaques fed the high-fat/low-fiber diet became more similar to the gut microbiota of humans.

## Background

The intestine is home to trillions of bacteria, which in number equal or even outnumber the number of host cells [1]. Accumulating evidence points to a link between the gut microbiota and several common diseases, including obesity [2–4], diabetes [5, 6], Crohn's disease [7], ulcerative colitis [8], rheumatoid diseases [9], cardiovascular disease [10, 11], and colorectal cancer [12]. Recent evidence also links changes in the gut microbiota to certain mental disorders [13, 14].

In order to establish causality between a given alteration of the gut microbiota and disease, rodent models are most frequently used. Previous studies have clearly demonstrated that the mouse gut microbiome is very different from that of humans [15–17]. Non-human primates (NHPs) are seemingly more biologically relevant animal models for humans, but very little information on their microbiomes is available. In captivity, *Macaca fascicularis*, the cynomolgus macaque, has been reported to have undergone a loss of native microbes, and the primary bacterial genera in gut were reported to be *Prevotella* and *Bacteroides*, similar to dominant genera in the human gut [18, 19]. Thus, detailed studies on the composition and functional capacity of the gut microbiota of the cynomolgus macaque are warranted in order to examine the potential of this model for biomedical research.

Previous studies have explored the gut microbiota of different monkey species using 16S rRNA gene amplicon sequencing, providing little information on gene identity and function of the monkey gut microbiome [18–21]. In the present study, fecal samples from 20 cynomolgus macaques were used for metagenomics sequencing, resulting in the generation of a catalog comprising 1.9M NR bacterial genes. Comparison of the human, pig, mouse, and cynomolgus macaque gut microbiomes demonstrated that the cynomolgus macaque gut microbiome is more similar to that of human than those of pig and mouse at the gene level. We observed that the gut microbiota of cynomolgus macaques fed either a low-fat/high-fiber diet or a high-fat/low-fiber diet exhibited differences in composition and functional potential, which to a certain degree mimicked those observed in humans shifted between intake of a low-fat/high-fiber diet and a high-fat/low-fiber diet [22]. We envisage that the present gut bacterial gene catalog and the functional characterization will serve as a valuable reference and resource for biomedical research using the cynomolgus macaque as a model.

## Data Description

To establish a *M. fascicularis*, the cynomolgus macaque, gut microbial gene catalog, fecal samples from 20 cynomolgus macaque individuals were collected. The animals were divided into two groups and fed either a low-fat/high-fiber diet or a high-fat/low-fiber diet for three months. Further details are given in the Methods section. Total DNA was extracted from freshly collected fecal samples from all animals and used for sequencing on the Illumina HiSeq2000 platform as described previously [1]. In total, 140 Gb of data were generated, with an average of 7 Gb per sample (Additional File 1). The raw data were filtered with a quality-control cutoff (adapter sequence <15 bp, “N” base < 3bp, Q >20, final length >30), and host sequences were removed by alignment against the *M. fascicularis* genome (National Center for Biotechnology Information [NCBI] accession no. NC_02 2272.1 - NC_02 2292.1), resulting in 131 Gb of clean data used for assembly and open reading frames (ORFs) prediction using SOAPdenovo [23] and Metagene2 [24], respectively. Redundant ORFs from each sample were removed by CD-HIT [25], providing a 1.9-M NR cynomolgus macaque gut microbial gene catalog. The gene profiles were generated by mapping clean data to the gene catalog with soap2.22 [26]. The genes in the catalog were aligned against the NCBI-NR, the Kyoto Encyclopedia of Genes and Genomes (KEGG) [27], and the carbohydrate-active enzymes (CAZy) [28] database to obtain taxonomic and functional annotation.

## Analyses

### Construction of cynomolgus macaque gut bacterial gene catalog

*De novo* assembly, gene prediction, and elimination of redundant genes were performed as previously described [29], generating a NR geneset comprising 1,991,169 ORFs with an average length of 757 bp.

**Figure 1:**
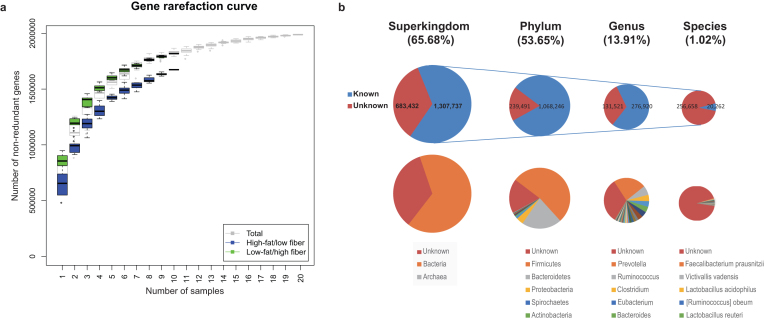
Rarefaction curve based on gene numbers and taxonomic annotation of the cynomolgus macaque gut bacterial gene catalog. **(a)** Rarefaction curve based on the gene numbers of all cynomolgus macaque samples and the individual subgroups. **(b)** Taxonomic annotation of 1.9-M cynomolgus macaque gut bacterial gene catalog. More than 65% of the genes from the cynomolgus macaque gut bacterial gene catalog could be annotated to the bacterial superkingdom, and 13.91% of the genes could be annotated to the genus level.

A rarefaction analysis based on gene number revealed a curve approaching saturation with 15 samples. Incidence-based coverage estimator, Chao1 indices, further indicated that we captured 97.00% of the gut microbial genes in the samples (Fig. 1a).

We could taxonomically classify 65.68% of the NR genes with CARMA3 [30]. More than 99.99% of the annotated genes could be assigned to the bacteria superkingdom. Of these genes, 1,068,246 (53.65%) could be annotated to the phylum level. At the phylum level, 52.94% of the annotated genes could be annotated to Firmicutes and 21.25% of the genes could be annotated to Bacteroidetes. At the genus and the species level, 276,920 (13.91%) and 20,262 (1.02%) of the macaque gut bacterial genes could be annotated to the genus and the species level, respectively (Fig. 1b). At the genus level, most of the annotated genes (34.55%) belonged to *Prevotella*, followed by *Ruminococcus* (9.91%), *Clostridium* (6.73%), *Eubacterium* (6.12%), and *Bacteroides* (6.00%) (Fig. 1b). We also mapped the cynomolgus macaque gene catalog to the KEGG database [27]. We could map 1,057,148 (53.09%) genes to KEGG orthology (KO) levels, of which 775,931 (38.97%) genes had pathway information. Pathways related to genetic information processing (replication and repair and translation), metabolism (carbohydrates, amino acids, energy, and nucleotides), and environmental information processing (membrane transport) (Additional File 2a) dominated. Additionally, we mapped the cynomolgus macaque gut bacterial gene catalog to the CAZy database. We were able to map 67,995 (3.41%) of the cynomolgus macaque gut bacterial genes to 248 CAZy families (Additional File 2b).

### The characteristics of cynomolgus macaque gut microbiome

**Figure 2:**
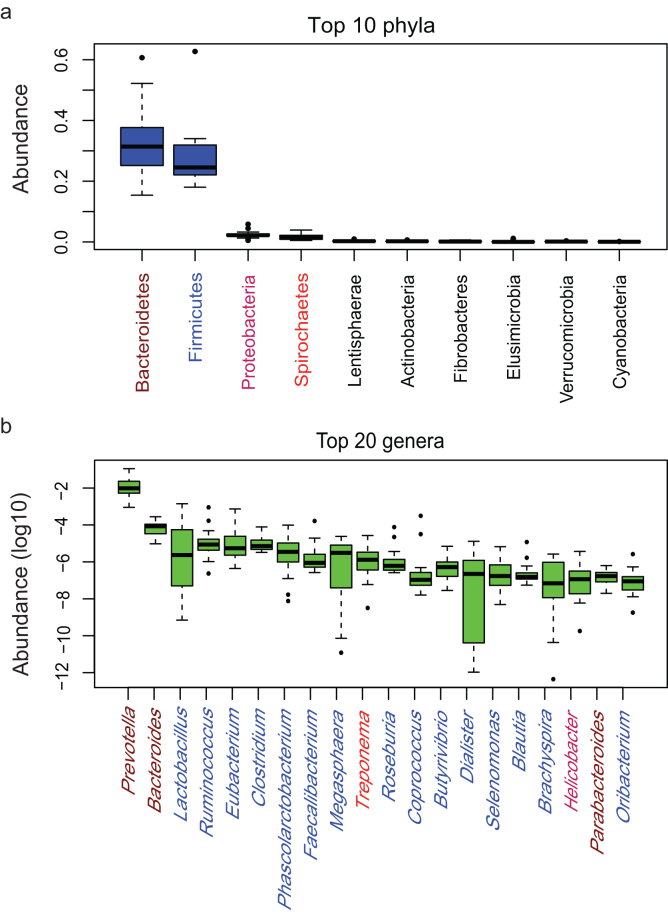
Characteristic of the cynomolgus macaque gut microbiota. **(a)**The top 10 phyla in the cynomolgus macaque gut microbiota. *Bacteroidetes* and *Firmicutes* are the main two phyla in the cynomolgus macaque gut microbiota. **(b)** The top 20 genera in the cynomolgus macaque gut microbiota. *Prevotella* is the main genus in the cynomolgus macaque gut microbiota.

Based on the taxonomical annotation, Bacteroidetes and Firmicutes were the two main phyla (Fig. 2a) and *Prevotella* and *Bacteroides* were the dominant genera (Fig. 2b) in the cynomolgus macaque gut microbiota. We found 80 core genera that were shared among all individuals with a lowest average abundance higher than 2.04e-07 (Additional File 3).

We identified three enterotype-like clusters in these 20 individual cynomolgus macaque samples, primarily driven by the highly abundant genera *Prevotella, Lactobacillus, and Ruminococcus* (Additional File 4a and 4b).

### Comparison with the human, mouse, and pig gut microbiomes

**Figure 3:**
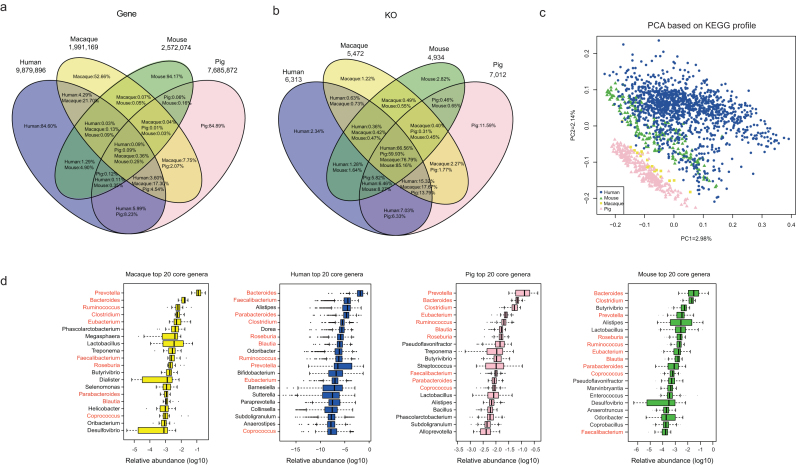
Comparison with the human, mouse, and pig gut microbiomes. **(a)** Unique NR genes in the cynomolgus macaque, human, pig, and mouse gut bacterial gene catalogs. Less than 0.4% genes overlapped between all four species, which emphasizes the marked differences between the cynomolgus macaque, human, pig, and mouse gut microbiome at the gene level. **(b)** Comparison of the cynomolgus macaque, human, pig, and mouse microbiotas based on KEGG annotation, which emphasizes the functional similarity between the cynomolgus macaque, human, pig, and mouse gut microbiota despite the marked differences at the gene level shown in a. **(c)** Principal component analysis–based on overlapping KOs of the cynomolgus macaque, human, mouse, and pig gut microbiota. **(d)** The top 20 core genera in the cynomolgus macaque, human, pig, and mouse gut microbiota. The 10 shared genera are marked in red.

The cynomolgus macaque gut bacterial catalog was compared with the human [31], pig [32], and mouse [15] catalogs. The human gut gene catalog includes 9,879,896 genes, the pig gut gene catalog includes 7,685,872 genes, and the mouse gut gene catalog includes 2,572,074 genes (Additional File 5). In the cynomolgus macaque gut bacterial gene catalog, 39.49% of the genes are included in the human gut bacterial gene catalog, 25.45% of the genes are present in the pig gut bacterial gene catalog, whereas only 0.6% of the genes are found in the mouse gut gene catalog. Moreover, less than 0.4% of cynomolgus macaque gut genes are shared by these four species, underscoring the marked differences between the gut microbiomes of these mammalian species at the gene level (Fig. 3a).

We randomly picked 1 million genes 10 times from the human, pig, and mouse gene catalog, respectively, and then mapped the high-quality reads generated from the cynomolgus macaque samples to these selections. The mapping rates to the human and pig microbial gene catalogs were 6.26% and 5.30%, respectively, whereas the mapping rate to the mouse catalog was only 0.51% (Additional File 6a, *P* value = 5.07e-09 in human vs pig). Additionally, high-quality reads from 20 samples of pig and mouse were also mapped to the 9.9-M human gene catalog. More reads of cynomolgus macaque gut microbiome (39.23%) could be mapped to the human gene catalog compared to reads from the pig (26.98%) and mouse (16.01%) (Additional File 6b). The pig gut microbiota exhibited a higher alpha diversity (Additional File 7a) than human, cynomolgus macaque, and mouse microbiomes.

At the functional level, 53.09% of the macaque and 48.77% of the mouse gut genes can be assigned to KOs, 42.10% of the human gut genes can be assigned to KOs, whereas about 35.79% of the pig gut genes can be assigned to KOs. The similarity of annotated KOs among the cynomolgus macaque, human, pig, and mouse gut microbiotas is very high (Fig. 3b). We identified 4,202 KOs involved in membrane transport and carbohydrate metabolism that are shared among the cynomolgus macaque, human, pig, and mouse gut microbiomes. Although the percentage of common KOs (82.87%) shared between human and cynomolgus macaque is less than the percentage shared between human and pig (95.37%), a principal component analysis (PCA) showed that the cynomolgus macaque gut microbiome is closer to the human microbiome than the pig microbiome (Fig. 3c). The distribution of CAZy classes was very similar among these four mammalian gut microbiomes (Additional File 2b).

We also identified bacterial genera that occurred in all samples from each of these four mammals. We term these core genera and identified 80 such core bacterial genera in the cynomolgus macaque (20 samples), 44 in human (1,267 samples) [31], 86 in pig (287 samples) [32], and 60 in mouse (184 samples) [15]. Comparing the core genera from the cynomolgus macaque, human, pig, and mouse, we found 32 genera that are shared among all four mammals (Additional File 8a), but we also noted that the abundance of these genera differed between each host (Additional File 8b). Among the 20 most abundant genera in each species, 10 genera are shared. These included *Prevotella*, *Bacteroides*, *Clostridium*, *Eubacterium*, *Parabacteroides*, *Ruminococcus*, *Faecalibacterium*, *Roseburia*, *Blautia*, and *Coprococcus*, which may constitute a core mammalian gut microbiota (Fig. 3d).

We compared the enterotype-like clusters of the cynomolgus macaque, the mouse, and the pig to human. In the human gut microbiota, enterotype-like clusters have been reported to be driven by *Bacteroides*, *Prevotella*, and *Ruminococcus* [12, 22, 33–35], and, in some cases, *Bifidobacterium* [5], *Alistipe*s, and *Faecalibacterium* [36]. In the cynomolgus macaque, we found that the enterotype-like clusters were driven by *Lactobacillus*, *Prevotella*, and *Ruminococcus*. In the mouse, the enterotype-like clusters were driven by *Alistipes*, *Akkermansia*, and *Clostridium*. In the pig, we observed that enterotype-like clusters were driven by *Streptococcus*, *Prevotella*, and *Lactobacillus* (Additional File 4). Based on the networks of the 32 core genera of these four mammals (Additional Files 9 and 10), we also analyzed the relationship of these enterotype-representative genera with other genera. We found that *Prevotella* correlated negatively with *Bacteroides* in human gut microbiota, but in cynomolgus macaque and pig microbiotas, *Prevotella* correlated positively with *Bacteroides*. Additionally, in the human and cynomolgus macaque gut microbiotas, *Ruminococcus* correlated positively with both *Blautia* and *Dorea*. Differences in enterotypes in humans have been linked to dietary patterns [22, 37]. However, to what extent the different patterns of enterotype-like clusters in these four species reflect differences in diets and/or genetics remains to be established. The finding that colonization by human microbiotas in germ-free mice only partial indicates that genetics may play a role [38–40].

### Diet-related changes in the cynomolgus macaque gut microbiota

**Figure 4:**
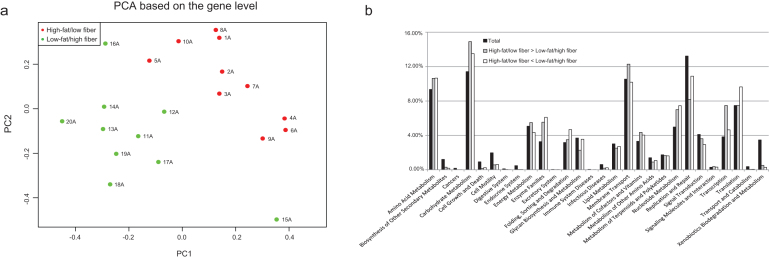
Diet-related differences in the cynomolgus macaque gut microbiota. **(a)** PCA of cynomolgus macaque samples based on gene profiles. **(b)** KEGG functional classification of the 82,120 gene makers. The black bars represent the total percentage in the 1.9-M gene catalog. The gray bars represent gene markers enriched in the high-fat/low-fiber diet group. The white bars represent gene markers enriched in the low-fat/high-fiber control group.

Comparison of cynomolgus macaques fed the low-fat/high-fiber or the high-fat/low-fiber diets for three months revealed that the latter group on average had slightly higher body mass (Wilcoxon rank sum test*, P* value <0.05) and elevated fasting blood glucose (Wilcoxon rank sum test*, P* value <0.05) (Additional File 11). Notably, the reads from cynomolgus macaque individuals that had consumed the high-fat/low-fiber diet showed a significantly higher mapping rate to the human and the pig genesets (*P* value = 2.06e-04 in human and *P* value = 3.25e-04 in pig), but not to the mouse gene sets (*P* value = 0.14). In response to these diets, we observed changes of alpha diversity. Intake of the high-fat/low-fiber diet tended to decrease alpha diversity, but the difference did not reach statistical significance (*P* value = 0.14) (Additional File 7b). However, individuals fed the high-fat/low-fiber diet could be clearly distinguished from the control group at the gene level (Fig. 4a). We found that 82,120 gene markers differed in abundance comparing the two groups (*P* value <0.01). Most of these marker genes are involved in metabolism of carbohydrates, amino acids, nucleotides, and vitamins.

Analysis of genera that differed significantly in abundance between the two groups of cynomolgus macaques was performed (Wilcoxon rank sum test, *P* value <0.05). We found five genera, including *Parabacteroides* and *Succinatimonas*, being enriched in individuals fed the high-fat/low-fiber diet, whereas in the gut microbiota of individuals fed the low-fat/high-fiber diet, 11 genera, including *Ruminococcus, Roseburia, and Eubacterium*, were enriched (Additional File 12). KOs involved in carbohydrate metabolism, energy metabolism, membrane transport, and transcription were more abundant in individuals fed the high-fat/low-fiber diet compared to the low-fat/high-fiber diet (Fig. 4b). At the module or pathway level, the gut microbiota of cynomolgus macaques fed a high-fat/low-fiber diet was functionally enriched in saccharide, polyol, and lipid transport systems; phosphate and amino acid transport systems; and metabolic modules involved in branched-chain amino acid, carbohydrate, lipid, and methane metabolism. The gut microbiota of cynomolgus macaques fed a low-fat/high-fiber diet was functionally enriched in bacterial secretion system, protein export, purine metabolism, and lipopolysaccharide biosynthesis (Additional Files 13 and 14). Since the two diets differ both in fat and fiber content, the observed changes most likely reflect changes in both of these constituents. Differences in the composition and functional potential of the gut microbiota in response to a low-fat/high-fiber diet or a high-fat/low-fiber diet have also been reported in a human study [22]. We observed that some of the KEGG pathways that differed in abundance in the human study in response to the different diet, including bacterial secretion system and protein export, also differed in response to the two diets in cynomolgus macaques.

### The distribution of predicted phage sequences in gut microbiome of cynomolgus macaques

In total 311,017 (15.62%) of the genes in the cynomolgus macaque gut gene catalog were predicted as bacterial phage sequences by Metafinder [41] (ANI >1.7%). Similar ratios of phage genes in the human, mouse, and pig gut gene catalogs were also predicted using the same pipeline (Additional File 15). By comparing the distribution of these predicted phage genes between cynomolgus macaques fed the high-fat/low-fiber diet and low-fat/high-fiber diet, 56,800 gene were found to differ significantly in abundance between the two groups (Wilcoxon rank sum test, *P* < 0.05) (Additional File 16). Of these, 43,602 were enriched in the control group, while 13,198 genes were enriched in macaques fed the high-fat/low-fiber diet. Additionally, the heat map clearly separated these genes between the two diet groups (Additional File 17).

## Discussion

Here, we constructed a gut bacterial gene catalog of *M. fascicularis*, the cynomolgus macaque, comprising 1,991,169 NR genes and compared it with the human, mouse, and pig gut bacterial gene catalogs. This catalog represents the first geneset generated from an NHP and provides a comprehensive reference resource for metagenomics-based research. The comparison with human, pig, and mouse demonstrates that the overlap between different mammals is very modest at the gene level but high at the KO functional level. Jonathan et al. reported that the gut microbiotas of captive NHPs have undergone humanization [18]. Our results also show that the cynomolgus macaque gut microbiome is more similar to the human gut microbiome than the other analyzed mammalian species. However, the degree of similarity is only slightly greater, and the comparisons emphasize the quite large differences at the gene levels among the cynomolgus macaque, human, pig, and mouse. However, similarity at the functional level is high between all species. Thus, from a purely metagenomics point of view, the use of cynomolgus macaques for biomedical research needs more research. Based on the high genetic similarity between human and cynomolgus macaque, it will be of interest to determine if colonization with human microbiotas will be more efficient in cynomolgus macaque than in pig or mouse. We demonstrate that intake of diets with different content of fat and fiber elicited pronounced differences in the gut microbiota of cynomolgus macaques and that some of these differences recapitulated differences in humans ingesting a low-fat/high-fiber diet or a high-fat/low-fiber diet [22].

We were able to define a set of core gut bacterial genera based on the available data on the gut microbiomes established by shotgun sequencing of fecal samples from four mammalian species. *Prevotella*, *Bacteroides*, *Clostridium*, *Eubacterium*, *Parabacteroides*, *Ruminococcus*, *Faecalibacterium*, *Roseburia*, *Blautia*, and *Coprococcus* were found to be the dominant bacterial genera present in gut microbiotas of human, cynomolgus macaque, pig, and mouse. However, the relative abundance of these genera varies profoundly among the four species.

A previous case-control comparison of enteric viromes in captive rhesus macaques showed several viruses associated with idiopathic chronic diarrhea [42]. We explored the presence of bacteria phages in the cynomolgus macaque gut microbiome. Interestingly, 15.6% of the genes in the current cynomolgus macaque gut gene catalog could be annotated as bacterial phages. Furthermore, the relative abundance of a subset of these phages differed significantly between cynomolgus macaques fed the low-fat/high-fiber diet and those fed the high-fat/low-fiber diet, underscoring that phages are abundant in the gut and may change in abundance in response to dietary intake. Thus, phages may play an important role in gut homeostasis, but the difference in relative abundance in response to dietary intake may also simply reflect changes in the relative abundance of their bacterial hosts [11].

## Methods

### Animals, sample collection, and transportation

Fresh feces from 20 cynomolgus macaques (*M. fascicularis*) aged 13–16 years were sampled. The animals were housed at room temperature with a 12-hour light/dark cycle at the JinJieKang Biotechnology Company, Yunnan, China, following guidelines approved by the Association for Assessment and Accreditation of Laboratory Animal Care. The experimental protocol was approved by the Animal Care and Use Committee at the JinJieKang Biotechnology Company. The animals had *ad libitum* access to water. The animals were divided into two groups of 10 animals. Ten males were fed a low-fat/high-fiber diet (8% of energy from fat, 131 g fiber/kg), and nine males and one female were fed a high-fat/low-fiber diet (39% of energy from fat, 20 g fiber/kg) for three months. After three months, the animals were weighed and blood was collected for blood glucose measurements at Kunming Jinyu Medical Laboratory Co., Ltd. Fresh feces were collected, immediately frozen, and kept on dry ice during transportation to BGI Shenzhen for further processing.

### DNA extractions and sequencing

DNA extraction was performed using 200 mg feces per sample following the method reported by Qin et al [29], except that cell lysis was performed by bead beating the samples twice for 30 seconds with an incubation of 2 minutes on ice between beatings. The concentration of fecal DNA was measured using Nanodrop. Following the manufacturer's instructions (Illumina), we constructed one DNA paired-end library with an insert size of 350 bp for each sample. Metagenomic sequencing was performed on the Illumina 2000 platform using a 100-bp paired-end strategy.

### Construction of the gene catalog

Raw reads were filtered with a quality-control cutoff (adapter sequence <15 bp, “N” base <3 bp, Q >20, final length >30) and host genomic DNA (NCBI accession no. NC_02 2272.1 - NC_02 2292.1). An average of 3.49% of the raw reads, which were of low quality or mapped to the host genome DNA, were removed. The remaining reads were considered high-quality reads. We obtained 131 Gb of high-quality data with an average of 6.55 Gb per sample. To construct a cynomolgus macaque gut microbial gene catalog, we assembled the Illumina reads from each sample into longer contigs with the SOAPdenovo2 software (SOAPdenovo2, RRID: SCR_01 4986) [23, 29]. A total of 56.43% of the reads were assembled into 2.02 million contigs with a length exceeding 500 bases. Metagene2 [24, 29] was used to predict ORFs in contigs obtained for each sample, with an average 220,862 ORFs per sample. An NR geneset comprising ∼1.9 M genes was constructed by pairwise comparison of all genes in all samples, using CD-HIT (CD-HIT, RRID:SCR_007105) [25] with identity of >95% and overlap of >90%. Taxonomic assignments (taxonomic database: version March 2012) were made using CARMA3 [30] on the basis of Basic Local Alignment Search Tool for Proteins (BLASTP) against the NCBI-NR database (version September 2013, the same version used for the mouse and pig gut microbiome catalogs).

### Functional annotation of gene catalog

We translated the nucleotide sequences of gene catalog into amino acid sequences, then aligned against the proteins or domains in eggNOG v3 (eggNOG, RRID:SCR_002456) [43] and KEGG v59 (KEGG, RRID:SCR_012773) [27] databases using BLASTP (v2.2.24, default parameter except that -F: F). KEGG annotation was performed using an in-house pipeline, where each protein was assigned to a KO when the highest-scoring annotated hit(s) contained at least one alignment over 60 hits.

### Quantification of gene relative abundance

High-quality reads from each sample were aligned against the gene catalog by SOAP2.22 (SOAPaligner/soap2, RRID:SCR_005503) [26] (with default parameters except for -r 2 -l 30 -M 4 -p 2 -v 10). The relative abundance of each gene in each sample was determined as previously described [5].

### Quantification of genus and KO relative abundances

For the relative abundance profile at the genus level, we used the phylogenetic assignment of each gene and summed the relative abundance of genes from the same genus to calculate the abundance of a particular genus. The relative abundance of each genus in a sample constituted the genus profile of that sample. Using the same method, the relative abundance of each KO was calculated from the sum of the relative abundances of the corresponding genes.

### KEGG module and pathway enrichment analysis

One-tailed Wilcoxon rank-sum test was performed for all the KOs that occurred in more than five samples and adjusted for multiple testing using the Benjamin-Hochberg procedure. The Z-score for each KO could then be calculated:

\begin{equation*}
{{\boldsymbol{\rm Z}}_{{\boldsymbol{\rm K}}{{\boldsymbol{\rm O}}_{\boldsymbol{\rm i}}}}} = {\theta ^{ - 1}}(1 - {{\boldsymbol{\rm P}}_{{\boldsymbol{\rm K}}{{\boldsymbol{\rm O}}_{\boldsymbol{\rm i}}}}})
\end{equation*}

where θ^−1^ is the inverse normal cumulative distribution, ${{\boldsymbol{\rm P}}_{{\boldsymbol{\rm K}}{{\boldsymbol{\rm O}}_{\boldsymbol{\rm i}}}}}$ is the adjusted P value for that KO. The aggregated Z-score for a KEGG pathway (or module) is then:

\begin{equation*}
{{\rm{Z}}_{{\rm{pathway}}}} = \frac{{{\bf 1}}}{{\sqrt {\boldsymbol{\rm k}} }}\sum {{\boldsymbol{\rm Z}}_{{\boldsymbol{\rm K}}{{\boldsymbol{\rm O}}_{\boldsymbol{\rm i}}}}}
\end{equation*}

where k is the number of KOs involved in the pathway (or module).

We corrected the background distribution of Z_pathway_ by subtracting the mean (μ_k_) and dividing by the s.d. (σ_k_) of the aggregated Z-scores of 1,000 sets of k KO, chosen randomly from the whole metabolic KO network:

\begin{equation*}
{{\rm{Z}}_{{\rm{adjustedpathway}}}} = \frac{{{{\boldsymbol{\rm Z}}_{{\boldsymbol{\rm pathway}}}} - {{\boldsymbol{\rm \mu }}_{\boldsymbol{\rm k}}}}}{{{{\boldsymbol{\rm \sigma }}_{\boldsymbol{\rm k}}}}}.
\end{equation*}

The Z_adjustedpathway_ was used as the final reporter score for evaluating the enrichment of specific pathways or modules. A reporter score of $ \ge $1.6 (90% confidence according to normal distribution) could be used as a detection threshold for significantly differentiating pathways. This is the same procedure as previously described [44, 45].

### Rarefaction curve analysis

Rarefaction analysis was performed to assess the gene richness. For a given number of samples, we performed random sampling 100 times in the cohort with replacement and estimated the total number of genes present in these samples by the Chao1 richness estimator [46].

### Enterotypes-like cluster

Genus relative abundances were used for analysis of partitioning around means (PAM)-based enterotypes-like clusters in cynomolgus macaque, pig, and mouse samples [15, 32]. In this study, the R package “stats” was used to perform a hierarchical clustering of samples using Jensen-Shannon distances followed by PCA using the R package “ade4.”

### Comparison with the human, mouse, and pig gut gene catalogs

The human [31], mouse [15], and pig [32] gut genesets were compared to the cynomolgus macaque geneset. If two and more genes had >95% identity and >90% overlap with the query, we considered the genes to be identical. For comparison at the functional level, shared KOs were identified and computed by unique KO ID.

### Differences in taxonomic abundance between diets

We analyzed differences in abundance at the phylum, genus, and species level using Wilcoxon rank sum test (*P* <0.05).

### Association between diets and metagenomic markers

To identify associations between metagenome profiles and the two diets, a 2-tailed Wilcoxon rank-sum test [5] implemented in R (R package stats) was used.

### Phage genes identification and comparison between the two diet groups

Phage genes were identified from the human, mouse, pig, and cynomolgus macaque gut gene catalogs using Metafinder [41] (ANI >1.7%). Phage genes that differed in abundance between samples from cynomolgus macaques fed the low-fat/high-fiber diet and the high-fat/low-fiber diet were selected by Wilcoxon rank sum test (P < 0.05).

## Supplementary Material

GIGA-D-17-00351_(Original_Submission).pdf

GIGA-D-17-00351_Revision_1.pdf

GIGA-D-17-00351_Revsion_2.pdf

Response_to_Reviewer_Comments_Original_Submission.pdf

Response_to_Reviewer_Comments_Revision_1.pdf

Reviewer_1_Report_(Original_Submission) -- Ilias Lagkouvardos, PhD 02-01-2018 Reviewed

Reviewer_1_Report_(Revision_1) -- Ilias Lagkouvardos, PhD 4/6/2018 Reviewed

Reviewer_2_Report_(Original_Submission) -- Intawat Nookaew 2/21/2018 Reviewed

Reviewer_2_Report_(Revision_1) -- Intawat Nookaew 4/24/2018 Reviewed

Additional Files

## Avaliablity of Supporting data

The metagenomic shotgun sequencing data for all samples have been deposited in the EBI database under the accession code PRJEB22765. Supplemental data are available in the *GigaScience* database, GigaDB [47]. The data has also been uploaded to the China National GeneBank (CNGB) Microbiome Database, from which they can be accessed at https://db.cngb.org/microbiome/genecatalog/macaca_fascicularis.

## Additional files

**Additional file 1**. Data production from cynomolgus macaque fecal samples.

**Additional file 2**. KEGG pathway classification and CAZy classification.

a. KEGG pathway classification. 53.09% of the cynomolgus macaque gene catalog could be annotated to the KO level.

b. CAZy classification. 3.41% of the cynomolgus macaque gene catalog could be annotated in the CAZy database.

**Additional file 3**. The average abundance of the 80 core genera shared among all cynomolgus macaque individuals.

**Additional file 4**. The enterotype-like cluster in the cynomolgus macaque, mouse, and pig samples.

a. Enterotype-like clusters in the cynomolgus macaque samples.

b. Abundances of the main contributors to each enterotype-like cluster in the cynomolgus macaque samples.

c. Enterotype-like clusters in the mouse samples.

d. Abundances of the main contributors to each enterotype-like cluster in mouse samples.

e. Enterotype-like clusters in the pig samples.

f. Abundances of the main contributors to each enterotype-like cluster in the pig samples.

**Additional file 5**. The general features of the human, macaque, mouse, and pig gut bacterial gene catalogs.

**Additional file 6**. Mapping ratio of cynomolgus macaque, human, pig and mouse samples.

a. Average mapping ratio of cynomolgus macaque sample reads to 1 million genes randomly selected (10 times) from the cynomolgus macaque, human, pig and mouse gene catalogs.

b. Average mapping ratio of 20 samples from mouse, pig, cynomolgus macaque, and human mapped to 9.9M human gut gene catalogs.

**Additional file 7**. Alpha diversity

a. Alpha diversity calculated as Shannon effective of the cynomolgus macaque gut microbiota compared to the human, pig, and mouse gut microbiota. The alpha diversity of pig gut microbiota is highest compared to the gut microbiota of the other three species, and the alpha diversity of the human gut microbiota is lowest.

b. Alpha diversity calculated as Shannon effective of the cynomolgus macaque gut microbiota in samples from animals fed the low-fat/high fiber diet or the high-fat/low fiber diet, with the latter tending to exhibit lower alpha diversity.

**Additional file 8**. Core genera in the gut microbiota of the cynomolgus macaque, pig, human, and mouse.

a. Venn diagram of core genera in the cynomolgus macaque, pig, human, and mouse;

b: heatmap of the 32 mammalian core genera.

**Additional file 9**. Genera networks of 32 mammalian core genera in each mammalian gut microbiota.

a. Genera network of 32 mammalian core genera in the human gut microbiota.

b. Genera network of 32 mammalian core genera in the cynomolgus macaque gut microbiota.

c. Genera network of 32 mammalian core genera in the mouse gut microbiota.

d. Genera network of 32 mammalian core genera in the pig gut microbiota.

The size of the node is proportional to the genus abundance. Node color corresponds to phylum taxonomic classification. Edge color represents positive (red) and negative (green) correlations, and the edge thickness is equivalent to the absolute values of Spearman correlation coefficient. (q-value < 0.05)

**Additional file 10**. Correlative relationships of 32 mammalian core genera showed in additional file 9.

**Additional file 11**. Phenotypic information of all cynomolgus macaque individuals.

**Additional file 12**. Analysis of differences in abundance at the phylum, genus and species level.

**Additional file 13**. Enrichment of KEGG modules in the gut microbiotas of animal fed the low-fat/high fiber diet and the high-fat low fiber diet.

**Additional file 14**. Enrichment of KEGG pathways in cynomolgus macaques fed the high-fat/low fiber or the low-fat/high fiber diets.

**Additional file 15**. Summary of the phage genes identified in the cynomolgus macaque, human, pig, and mouse gut microbiome gene catalogs.

**Additional file 16**. List of predicted phage genes that differ significantly in abundance between the high-fat/low fiber diet and low-fat/high fiber diet fed cynomolgus macaque groups.

**Additional file 17**. Heatmap of the abundance of predicted phage genes that differ significantly in abundance between the high-fat/low fiber diet and low-fat/high fiber diet fed cynomolgus macaque groups. We selected phage genes with zero abundance in all the low-fat/high fiber diet fed cynomolgus macaque individuals and exhibited non-zero abundance in all the high-fat/low fiber diet fed cynomolgus macaque individuals and vice versa, i.e., zero abundance in all the high-fat/low fiber diet fed cynomolgus macaque individuals and non-zero abundance in all the low-fat/high fiber diet fed cynomolgus macaque individuals

**Additional file 18**. Selected scripts used for bioinformatics analyses.

## Abbreviations

BLASTP: Basic Local Alignment Search Tool for Proteins; CAZy: carbohydrate-active enzymes; KEGG: Kyoto Encyclopedia of Genes and Genomes; KO: KEGG orthology; NCBI: National Center for Biotechnology Information; NHP: nonhuman primates; NR: nonredundant; ORF: open reading frame; PCA: principal component analysis.
