# Supplementary material for: Establishment of a Macaca fascicularis gut microbiome gene catalog and comparison with the human, pig, and mouse gut microbiomes
Source: Gigascience. 2018 Aug 18;7(9):giy100. doi: 10.1093/gigascience/giy100 (PMC6137240; doi:10.1093/gigascience/giy100)
Supplement: GIGA-D-17-00351_(Original_Submission).pdf [file giy100_giga-d-17-00351_(original_submission).pdf]

GigaScience

Establishment of a Macaca fascicularis gut microbiome gene catalog and comparison with the human, pig and mouse gut microbiomes

--Manuscript Draft--

**Manuscript Number:**	GIGA-D-17-00351
**Full Title:**	Establishment of a Macaca fascicularis gut microbiome gene catalog and comparison with the human, pig and mouse gut microbiomes
**Article Type:**	Research
**Funding Information:**	
**Abstract:**	Macaca fascicularis, the cynomolgus macaques, is a widely used model in biomedical research and drug development as its genetics and physiology are close to humans. Detailed information on the cynomolgus macaques gut microbiota, the functional interplay between the gut microbiota and host physiology, and possible similarities to humans and other mammalians is very limited. In this study, faecal samples from 20 cynomolgus macaques individuals were used for metagenomic sequencing to construct the first catalog of gut bacterial genes in the cynomolgus macaques. In total 1.9 million non-redundant bacterial genes were identified of which 39.49% are present in the human gut bacterial gene catalog and 25.45% are present in the pig gut bacterial gene catalog, whereas only 0.6% of the genes are present in the mouse gut gene catalog. By contrast, at the function levels, more than 76% KOs are shared between all four mammalians. Thirty-two highly abundant bacterial genera could be defined as core genera of mammalians. We demonstrated significant differences in the composition and functional potential of the gut microbiota as well as in the distribution of predicted bacterial phages sequences in cynomolgus macaques fed either a low-fat/high fiber diet or a high-fat/low fiber diet. Interestingly, the gut microbiota of the cynomolgus macaques fed the high-fat/low fiber diet became more similar to the gut microbiota of humans.<br><br>Keywords: Macaca fascicularis, gene catalog, gut microbiome, core genera of mammalian, high-fat/low fiber diet, low-fat/high fiber diet
**Corresponding Author:**	Liang Xiao<br><br>CHINA
**Corresponding Author Secondary Information:**	
**Corresponding Author's Institution:**	
**Corresponding Author's Secondary Institution:**	
**First Author:**	Xiaoping Li
**First Author Secondary Information:**	
**Order of Authors:**	Xiaoping Li
	Suisha Liang
	Zhongkui Xia
	Jing Qu
	Huan Liu
	Chuan Liu
	Huanming Yang
	Jian Wang
	Lise Madsen

Powered by Editorial Manager® and ProduXion Manager® from Aries Systems Corporation

	Yong Hou
	Junhua Li
	Huijue Jia
	Karsten Kristiansen
	Liang Xiao
**Order of Authors Secondary Information:**	
**Opposed Reviewers:**	
**Additional Information:**	
**Question**	**Response**
Are you submitting this manuscript to a special series or article collection?	No
**Experimental design and statistics**<br><br>Full details of the experimental design and statistical methods used should be given in the Methods section, as detailed in our Minimum Standards Reporting Checklist. Information essential to interpreting the data presented should be made available in the figure legends.<br><br>Have you included all the information requested in your manuscript?	Yes
**Resources**<br><br>A description of all resources used, including antibodies, cell lines, animals and software tools, with enough information to allow them to be uniquely identified, should be included in the Methods section. Authors are strongly encouraged to cite Research Resource Identifiers (RRIDs) for antibodies, model organisms and tools, where possible.<br><br>Have you included the information requested as detailed in our Minimum Standards Reporting Checklist?	Yes
**Availability of data and materials**<br><br>All datasets and code on which the conclusions of the paper rely must be either included in your submission or deposited in publicly available repositories (where available and ethically appropriate), referencing such data using a unique identifier in the references and in the "Availability of Data and Materials"	Yes

Powered by Editorial Manager® and ProduXion Manager® from Aries Systems Corporation

section of your manuscript.<br><br>Have you have met the above requirement as detailed in our Minimum Standards Reporting Checklist?	

Manuscript Click here to download Manuscript Manuscript_final.docx

Establishment of a *Macaca fascicularis* gut microbiome gene catalog and comparison with the human, pig and mouse gut microbiomes

Xiaoping Li[1,2,3,#], Suisha Liang[1,2,3,#], Zhongkui Xia[1,2,3], Jing Qu[1,2,6], Huan Liu[1,2], Chuan Liu[1,2,3], Huanming Yang[1,2,4], Jian Wang[1,2,4], Lise Madsen[1,7,8], Yong Hou[1,2], Junhua Li[1,2,3,5], Huijue Jia[1,2,3], Karsten Kristiansen[1,2,8*], Liang Xiao[1,2*]

1 BGI-Shenzhen, Shenzhen 518083, China,

2 China National GeneBank, BGI-Shenzhen, Shenzhen 518120, China,

3 Shenzhen Key Laboratory of Human Commensal Microorganisms and Health Research, BGI-Shenzhen, Shenzhen 518083, China,

4 James D. Watson Institute of Genome Sciences, Hangzhou 310058, China,

5 School of Bioscience and Biotechnology, South China University of Technology, Guangzhou, 510006, China

6 BGI Innovation College of QingDao University, Qingdao, 266071, China

7 Laboratory of Genomics and Molecular Biomedicine, Department of Biology, University of Copenhagen, 2100 Copenhagen Ø, Denmark

8 Institute of Marine Research (IMR), Postboks 1870, Nordnes, N-5817, Bergen, Norway.

# These authors contributed equally to this work

* Corresponding authors

Abstract

Macaca fascicularis, the cynomolgus macaques, is a widely used model in biomedical research and drug development as its genetics and physiology are close to humans. Detailed information on the cynomolgus macaques gut microbiota, the functional interplay between the gut microbiota and host physiology, and possible similarities to humans and other mammalians is very limited. In this study, faecal samples from 20 cynomolgus macaques individuals were used for metagenomic sequencing to construct the first catalog of gut bacterial genes in the cynomolgus macaques. In total 1.9 million non-redundant bacterial genes were identified of which 39.49% are present in the human gut bacterial gene catalog and 25.45% are present in the pig gut bacterial gene catalog, whereas only 0.6% of the genes are present in the mouse gut gene catalog. By contrast, at the function levels, more than 76% KOs are shared between all four mammalians. Thirty-two highly abundant bacterial genera could be defined as core genera of mammalians. We demonstrated significant differences in the composition and functional potential of the gut microbiota as well as in the distribution of predicted bacterial phages sequences in cynomolgus macaques fed either a low-fat/high fiber diet or a high-fat/low fiber diet. Interestingly, the gut microbiota of the cynomolgus macaques fed the high-fat/low fiber diet became more similar to the gut microbiota of humans.

Keywords: *Macaca fascicularis*, gene catalog, gut microbiome, core genera of mammalian, high-fat/low fiber diet, low-fat/high fiber diet

Background

The intestine is home to trillions of bacteria, which in number equal or even outnumber the number of host cells[1]. Accumulating evidence points to a link between the gut microbiota and several common diseases, including obesity[2-4], diabetes[5, 6], Crohn's disease[7], ulcerative colitis[8], rheumatoid diseases[9], cardiovascular disease(CVD)[10, 11], and colorectal cancer[12]. Recent evidence also links changes in the gut microbiota to certain mental disorders[13, 14].

In order to establish causality between a given alteration of the gut microbiota and disease, rodent models are most frequently used. Previous studies have clearly demonstrated that the mouse gut microbiome is very different that of humans[15-17]. Non-human primates (NHPs) are seemingly more biologically relevant animal models for humans, but very little information on their microbiome is available. In captivity, *Macaca fascicularis*, the cynomolgus macaques, has been reported to have undergone a loss of native microbes, and the primary bacterial genera in gut were reported to be *Prevotella* and *Bacteroides*, similar to dominant genera in the human gut[18, 19]. Thus, detailed studies on the composition and functional capacity of the gut microbiota of the cynomolgus macaques are warranted in order to examine the potential of this model for biomedical research.

Previous studies have explored the gut microbiota of different monkey species using 16S rRNA gene amplicon sequencing providing little information on gene identity and function of the monkey gut microbiome [18-21]. In the present study, fecal samples from twenty cynomolgus macaques were used for metagenomics sequencing resulting in the generation of a catalog comprising 1.9M non-redundant bacterial genes. Comparison of the human, pig,

mouse and cynomolgus macaques gut microbiomes demonstrated that the cynomolgus macaques gut microbial is slightly more similar to human than pig and mouse at the gene level. We observed that the gut microbiota of cynomolgus macaques fed either a low-fat/high fiber diet or a high-fat/low fiber diet exhibited differences in composition and functional potential, which to a certain degree mimicked those observed in humans shifted between intake of a low-fat/high fiber diet and a high-fat/low fiber diet[22]. The present gut bacterial gene catalog and the functional characterization will serve as a valuable reference and resource for biomedical research using the cynomolgus macaques as a model.

Data Description

To establish a *Macaca fascicularis*, the cynomolgus macaques, gut microbial gene catalog, fecal samples from 20 cynomolgus macaques individuals were collected. The animals were divided into two groups and fed either a low-fat/high fiber diet or a high-fat/low fiber diet for three months. Further details are given in Methods. Total DNA was extracted from freshly collected fecal samples from all animals and used for sequencing on the Illumina HiSeq2000 platform as described previously[1]. In total, 140 gigabases (Gb) data were generated with an average of 7Gb per sample (additional file 1). The raw data were filtered with a quality control cutoff (adapter sequence <15bp, 'N' base <3bp, Q>20, final length >30) and host sequences were removed by alignment against the *M. fascicularis* genome (NCBI accession no. NC_022272.1 - NC_022292.1), resulting in 131 Gb clean data used for assembly and open reading frames (ORFs) prediction using SOAPdenovo[23] and Metagene2[24], respectively. Redundant ORFs from each sample were removed by CD-HIT[25], providing a

1.9M non-redundant cynomolgus macaques gut microbial gene catalog. The gene profiles were generated by mapping clean data to the gene catalog with soap2.22[26]. The genes in the catalog were aligned against the NCBI-NR, the Kyoto Encyclopedia of Genes and Genomes (KEGG)[27] and the carbohydrate-active enzymes (CAZy)[28] database to obtain taxonomic and functional annotation.

Analyses

Construction of cynomolgus macaques gut bacterial gene catalog

Denovo assembly, gene prediction, and elimination of redundant genes were performed as previously described[29] generating a non-redundant (NR) gene set comprising 1,991,169 open reading frames (ORFs) with an average length of 757 base pairs (bp).

A rarefaction analysis based on gene number revealed a curve approaching saturation with 15 samples, and incidence-based coverage estimator, Chao1 indices, further indicated that we captured 97.00% of the gut microbial genes in the samples (Fig.1a).

We could taxonomically classify 65.68% of the NR genes with CARMA3[30]. More than 99.99% of the annotated genes could be assigned to the bacteria super kingdom. Of these genes, 1,068,246 (53.65%) could be annotated to the phylum level. At the phylum level, 52.94% of the annotated genes could be annotated to Firmicutes and 21.25% of the genes could be

annotated to Bacteroidetes. At the genus and the species level, 276,920 (13.91%) and 20,262 (1.02%) of the macaque gut bacterial genes could be annotated to the genus and the species level, respectively (Fig 1b). At the genus level, most of the annotated genes (34.55%) belonged to *Prevotella,* followed by *Ruminococcus* (9.91%), *Clostridium* (6.73%), *Eubacterium* (6.12%) and *Bacteroides* (6.00%) (Fig.1b). We also mapped the cynomolgus macaques gene catalog to the Kyoto Encyclopedia of Genes and Genomes (KEGG) database[27]. We could map 1,057,148 (53.09%) genes to KEGG orthology (KO) levels of which 775,931 (38.97%) genes had pathway information. Pathways related to genetic information processing (replication and repair and translation), metabolism (carbohydrates, amino acids, energy and nucleotides) and environmental information processing (membrane transport) (additional file 2a) dominated. Additionally, we mapped the cynomolgus macaques gut bacterial gene catalog to the CAZy database. We were able to map 67,995 (3.41%) of the cynomolgus macaques gut bacterial genes to 248 CAZy families (additional file 2b).

The characteristics of cynomolgus macaques gut microbiome

Based on the taxonomical annotation, Bacteroidetes and Firmicutes were the two main phyla (Fig 2a) and *Prevotella* and *Bacteroides* were the dominant genera (Fig 2b) in the cynomolgus macaques gut microbiota. We found 80 core genera that were shared among all individuals with a lowest average abundance higher than 2.04e-07 (additional file 3). A co-abundance genera network analysis showed that the *Prevotella* genus correlated positively with the highly abundant *Bacteroides* genus and exhibited a negative correlation with the

Anaerococcus genus. Furthermore, the *Bacteroides* genus exhibited a negative correlation with the *Paenibacillus* genus (Fig 2c).

We identified three enterotypes-like clusters in these 20 individual cynomolgus macaques samples, primarily driven by the highly abundant genera *Prevotella*, *Lactobacillus and Ruminococcus* (additional file 4).

Comparison with the human, mouse and pig gut microbiomes

The cynomolgus macaques gut bacterial catalog was compared with the human[31], pig[32] and the mouse[15] catalog. The human gut gene catalog includes 9,879,896 genes, the pig gut gene catalog 7,685,872 genes and the mouse gut gene catalog 2,572,074 genes. In the cynomolgus macaques gut bacterial gene catalog, 39.49% of the genes are included in the human gut bacterial gene catalog, 25.45% of the genes are present in the pig gut bacterial gene catalog, whereas only 0.6% of the genes are found in the mouse gut gene catalog. Moreover, less than 0.4% of cynomolgus macaques gut genes are shared by these four species, underscoring the marked differences between the gut microbiomes of these mammalian species at the gene level (Fig 3a).

We randomly picked 1 million genes 10 times from the human, pig and mouse gene catalog, respectively, and then mapped the high quality reads generated from the cynomolgus macaques samples to these selections. The mapping rates to the human and pig microbial gene catalogs were 6.26% and 5.30%, respectively, whereas the mapping rate to the mouse

catalog was only 0.51% (additional file 5a, *P* value=5.07e-09 in human vs pig). Additionally, high quality reads from 20 samples of pig and mouse were also mapped to the 9.9M human gene catalog. More reads of cynomolgus macaques gut microbiome (39.23%) could be mapped to the human gene catalog compared to reads from the pig (26.98%) and mouse (16.01%) (additional file 5b).The pig gut microbiota exhibited a higher alpha diversity (additional file 6a) than human, cynomolgus macaques, and mouse microbiomes.

At the functional level, the similarity between the cynomolgus macaques, human, pig and mouse gut microbiotas is very high (Fig 3b). We identified 4,202 KOs involved in membrane transport and carbohydrate metabolism that are shared between the cynomolgus macaques, human, pig and mouse gut microbiomes. Although the percentage of common KOs (82.87%) shared between human and cynomolgus macaques is less than the percentage shared between human and pig (95.37%), a PCA showed that the cynomolgus macaques gut microbiome is closer to the human than the pig microbiome (Fig 3c). The distribution of CAZy classes was very similar between these four mammalian gut microbiomes (additional file 2b)

We also identified bacterial genera that occurred in all samples from each of these four mammals. We term these core genera and identified 80 such core bacterial genera in the cynomolgus macaques (20 samples), 44 in human (1267 samples)[31], 86 in pig (287 samples)[32], and 60 in mouse (184 samples)[15]. Comparing the core genera from the cynomolgus macaques, human, pig and mouse, we found 32 genera that were shared between all four mammals (additional file 7a), but we also noted that the abundance of these genera

differed between each host (additional file 7b). Amongst the 20 most abundant genera in each species, 10 genera were shared. These included *Prevotella*, *Bacteroides*, *Clostridium*, *Eubacterium*, *Parabacteroides*, *Ruminococcus*, *Faecalibacterium*, *Roseburia*, *Blautia*, and *Coprococcus* which may constitute a core mammalian gut microbiota (Fig 3d).

We compared the enterotype-like clusters of the cynomolgus macaques, the mouse and the pig to human. In the human gut microbiota enterotype-like clusters have been reported to be driven by *Bacteroides*, *Prevotella*, and *Ruminococcus*[12, 22, 33-35], and in some cases *Bifidobacterium*[5], *Alistipe*s and *Faecalibacterium*[36]. In the cynomolgus macaques we found that the enterotype-like clusters were driven by *Lactobacillus*, *Prevotella* and *Ruminococcus*. In the mouse, the enterotype-like clusters were driven by *Alistipes*, *Akkermansia* and *Clostridium*, and finally, in the pig we observed that enterotype-like clusters were driven by *Streptococcus*, *Prevotella* and *Lactobacillus* (additional file 4). Differences in enterotypes in humans have been linked to dietary patterns[22, 37]. To what extent the different patterns of enterotype-like clusters in these four species reflect differences in diets and/or genetics remains to be established. The only partial colonization of human microbiotas in germ free mice indicates that genetics plays an important role[38-40].

Diet-related changes in the cynomolgus macaques gut microbiota

Comparison of the cynomolgus macaques fed the low-fat/high fiber or the high-fat/low fiber diets for 3 months revealed that the latter group on average had slightly higher body mass

(Wilcoxon rank sum test, *P* value<0.05) and elevated fasting blood glucose (Wilcoxon rank sum test, *P* value<0.05) (additional file 8). Notably, the reads from cynomolgus macaques individuals that had consumed the high-fat diet/low fiber diet showed significantly higher mapping rate to the human and the pig gene sets (*P* value=2.06e-04 in human and *P* value=3.25e-04 in pig), but not to the mouse gene set(*P* value=0.14). In response to these diets, we observed changes of alpha and beta diversity. Intake of the high-fat/low fiber diet decreased alpha diversity significantly (additional file 6b) and individuals fed the high-fat/low fiber diet could also be clearly distinguished from the control group at the gene level (Fig 4a). In total, we found that 82,120 gene markers differed in abundance comparing the two groups (*P* value<0.01). Most of these marker genes were enriched in metabolism functions, including carbohydrate metabolism, amino acid metabolism, nucleotide metabolism, cofactor, and vitamin metabolism. KOs involved in carbohydrate metabolism, energy metabolism, membrane transport, and transcription were more abundant in individuals fed the high-fat/low fiber diet compared to the low-fat/high fiber diet (Fig 4b). At the module or pathway levels, the gut microbiota of high fat/low fiber diet fed cynomolgus macaques was functionally enriched in saccharide, polyol, and lipid transport systems, phosphate and amino acid transport systems and metabolic modules involved in branched-chain amino acid, carbohydrate, lipid, and methane metabolism, while the gut microbiota of cynomolgus macaques fed a low-fat/high fiber diet was functionally enriched in bacterial secretion system, protein export, purine metabolism and lipopolysaccharide biosynthesis (additional file 9 and additional file 10). Since the two diets differ both in fat and fiber content, the observed changes most likely reflect changes in both of these constituents. In one human study,

differences in the composition and functional potential of gut microbiota in response to a low-fat/high fiber diet or a high-fat/low fiber diet has been reported[22]. We observed that some of the KEGG pathways that differed in abundance in the human study in response to the different diet, including bacterial secretion system and protein export, also differed in response to the two diets in our study.

The distribution of predicted phage sequences in gut microbiome of cynomolgus macaques

In total 311,017 (15.62%) of the genes in the cynomolgus macaques gut gene catalog were predicted as bacterial phage sequences by Metafinder[41](ANI >1.7%). By comparing the distribution of these predicted phage genes between cynomolgus macaques fed the high-fat/low fiber diet and low-fat/high fiber diet, 56,800 genes were found to differ significantly in abundance between the two groups (Wilcoxon rank sum test, $P<0.05$) (additional file 11). Of these, 43,602 were enriched in control group while 13,198 genes were enriched in macaques fed the high-fat/low fiber diet. Additionally, the heat map clearly separated these genes between the two diet groups (additional file 12).

Discussion

Here we constructed a gut bacterial gene catalog of *Macaca fascicularis*, the cynomolgus macaques, comprising 1,991,169 non-redundant genes. This catalog represents the first gene

set generated from a NHP and provides a comprehensive reference resource for metagenomics-based research. The comparison with human, pig and mouse demonstrates that the overlap between different mammalians is very modest at the gene level, but high at the KO functional level. Jonathan *et al* reported that the gut microbiotas of captive NHPs have undergone humanization [18]. Our results also show that the cynomolgus macaques gut microbiome is more similar to the human gut microbiome than the other analyzed mammalian species. However, the degree of similarity is only slightly greater, and the comparisons rather emphasize the quite large differences at the gene levels between cynomolgus macaques, human, pig and mice. However, similarity at the functional level is high between all species. Thus, from a purely metagenomics point of view the use of the cynomolgus macaques for biomedical research needs more research. Based on the high genetic similarity between humans and the cynomolgus macaques it will be of interest to examine if colonization with human microbiotas will be more efficient in the cynomolgus macaques than in pig or mice.

We demonstrate that intake of diets with different content of fat and fiber elicited pronounced differences in the gut microbiota of the cynomolgus macaques, and that some of these differences recapitulated differences in humans ingesting a low-fat/high fiber diet or a high-fat/low fiber diet[22].

We were able to define a set of core gut bacterial genera based on the available data on the gut microbiomes established by shotgun sequencing of fecal samples from four mammalian species. *Prevotella*, *Bacteroides*, *Clostridium*, *Eubacterium*, *Parabacteroides*, *Ruminococcus*,

Faecalibacterium, *Roseburia*, *Blautia*, and *Coprococcus* were found to be the dominant bacterial genera present in gut microbiotas of human, cynomolgus macaques, pig, and mouse. However, the relative abundance of these genera varies profoundly between the four species.

A previous case-control comparison of enteric viromes in captive rhesus macaques showed several viruses associated with idiopathic chronic diarrhea[42]. We explored the presence of bacteria phages in the cynomolgus macaques gut microbiome. Interestingly, 15.6% of the genes in the current cynomolgus macaques gut gene catalog could be annotated as bacterial phages. Furthermore, the relative abundance of a subset of these phages differed significantly between cynomolgus macaques fed the low-fat/high fiber diet and the high-fat/low fiber diet underscoring that phages are abundant in the gut and may change in abundance in response to dietary intake. Thus, phages may play important role in gut homeostasis, but the difference in relative abundance in response to dietary intake may also simply reflect changes in the relative abundance of their bacterial hosts[11].

Methods

Animals, sample collection and transportation.

Fresh feces were sampled from twenty cynomolgus macaques (*Macaca fascicularis*), 11-14 years old. The animals were housed at room temperature with a 12 h light/dark cycle at the JinJieKang Biotechnology Company, Yunnan, China following guidelines approved by the

Association for Assessment and Accreditation of Laboratory Animal Care. The experimental protocol was approved by the Animal Care and Use Committee at the JinJieKang Biotechnology Company. The animals had *ad libitum* access to water and the animals were divided into two groups of ten animals. Ten males were fed a low-fat/high fiber diet (8 % of energy from fat, 131 g fiber/ kg) and nine males and one female were fed a high-fat diet/low fiber diet (39 % of energy from fat, 20 g fiber/ kg) for three months. After the three months of feeding the animal were weighted and blood was collected for blood glucose measurements at Kunming Jinyu Medical Laboratory Co., Ltd. Fresh feces was collected, immediately frozen and kept on dry ice during transportation to BGI Shenzhen for further processing.

DNA extractions and sequencing

DNA extraction was performed using 200 mg feces per sample following the method reported by Qin et al[29], except that cell lysis was performed by bead beating the samples twice for 30 s with an incubation of 2 min on ice between beatings. The concentration of fecal DNA was measured using Nanodrop. Following the manufacturer's instructions (Illumina), we constructed one DNA paired-end (PE) library with an insert size of 350 base pairs (bp) for each sample. Metagenomic sequencing was performed on the Illumina 2000 platform by a 100 bp paired-end strategy.

Construction of the gene catalog

Raw reads were filtered with a quality control cutoff (adapter sequence <15bp, 'N' base <3bp, Q>20, final length >30) and host genomic DNA (NCBI accession no. NC_022272.1 - NC_022292.1). An average of 3.49% of the raw reads, which were of low quality or mapped to the host genome DNA were removed. The remaining reads were considered as high-quality reads. In total, we obtained 131 Gb high-quality data with an average of 6.9 Gb per sample. To construct a cynomolgus macaques gut microbial gene catalog, we assembled the Illumina reads from each sample into longer contigs with the SOAPdenovo software[23, 29]. A total of 56.43% of the reads were assembled into 2.02 million contigs with a length exceeding 500 bases. Metagene2[24, 29] was used to predict open reading frames (ORFs) in contigs obtained for each sample, with an average 220,862 ORFs per sample. A non-redundant gene set comprising ~1.9 M genes was constructed by pairwise comparison of all genes in all samples, using CD-HIT[25] with identity of >95% and overlap of >90%. Taxonomic assignments were made with CARMA3[30] on the basis of BLASTP against the NCBI-NR database. Functional annotations were made by BLASTP search against eggnog[43] and KEGG v59[27] databases.

Quantification of Gene relative abundance

High-quality reads from each sample were aligned against the gene catalog by SOAP2.22[26] (with default parameters except for -r 2 -l 30 -M 4 -p 2 -v 10). Relative abundance of each gene in each samples was determined as previously described[5].

Quantification of genus and KO relative abundances

We used the phylogenetic assignment of each gene and summed the relative abundance of genes from the same genus to calculate the abundance of a particular genus. Using the same method, the relative abundance of each KO was calculated from the sum of the relative abundances of the corresponding genes.

Rarefaction curve

Rarefaction analysis was performed to assess the gene richness. For a given number of samples, we performed random sampling 100 times in the cohort with replacement and estimated the total number of genes present in these samples by the Chao1 richness estimator[44].

Enterotypes-like cluster

Genus relative abundances were used for analysis of PAM-based enterotypes-like clusters in cynomolgus macaques, pig and mouse samples[15, 32]. In this study, the R package "stats" was used to perform a Hierarchical clustering of samples using Jensen-Shannon distances followed by PCA using the R package "ade4".

Comparison with the human, mouse and pig gut gene catalog

The human[31], mouse[15] and pig[32] gut gene sets were compared to the cynomolgus macaques gene set. If two and more genes had > 95% identity and >90% overlap with the query, we considered the genes to be identical. For comparison at the functional level, shared KOs were identified and computed by unique KO ID.

Association between diets and metagenomic markers

To identify associations between metagenome profiles and the two different diets, a two-tailed Wilcoxon rank-sum test[5] implemented in R(R package stats) was used.

Phage genes identification and comparison between the two diet groups

Phage genes were identified from the cynomolgus macaques gut gene catalog using Metafinder[41](ANI >1.7%). Phage genes that differed in abundance between samples from cynomolgus macaques fed the low-fat/high fiber diet and the high-fat/low fiber diet were selected by wilcoxon rank sum test($P<0.05$).

Availability of supporting data and materials

The metagenomic shotgun sequencing data for all samples have been deposited in the EBI database under the accession code PRJEB22765. Supplemental data is available in the

GigaScience database, GigaDB.

Declarations

List of abbreviations

NHP: Nonhuman primates; KEGG: Kyoto Encyclopedia of Genes and Genomes; ORF: open reading frames; NR: Non-redundant; KO: KEGG orthology; Gb: gigabases; bp: base pairs; PE: paired-end

Competing interests

The authors declare that they have no competing interests.

Authors' contributions

X.L., and L.X. conceived and directed the project. H.L., and X.L. oversaw the sample collection and provided phenotypic information. X.L., S.L., Z.X., J.Q., and C.L. performed the bioinformatic analyses and prepared figures and texts for the manuscript. X.L., and S.L. wrote the first draft of the manuscript. L.X., H.J., J.L. L.M. and KK made substantial revision of the manuscript. L.X., S.L., and J.Q. participated in discussions. All authors contributed to the revision of the manuscript.

Acknowledgments

This research was supported by the National Natural Science Foundation of China (Grant No. 81670606, 81673850), the Shenzhen Municipal Government of China (JSGG20160229172752028, JCYJ20160229172757249). We gratefully acknowledge colleagues at BGI-Shenzhen for DNA extraction, library construction, sequencing, and discussions.

Reference

1. Sender R, Fuchs S and Milo R. Are We Really Vastly Outnumbered? Revisiting the Ratio of Bacterial to Host Cells in Humans. Cell. 2016;164 3:337-40. doi:10.1016/j.cell.2016.01.013.
2. Turnbaugh PJ, Ley RE, Mahowald MA, Magrini V, Mardis ER and Gordon JI. An obesity-associated gut microbiome with increased capacity for energy harvest. Nature. 2006;444 7122:1027-131. doi:10.1038/nature05414.
3. Cani PD, Bibiloni R, Knauf C, Waget A, Neyrinck AM, Delzenne NM, et al. Changes in gut microbiota control metabolic endotoxemia-induced inflammation in high-fat diet-induced obesity and diabetes in mice. Diabetes. 2008;57 6:1470-81. doi:10.2337/db07-1403.
4. Le Chatelier E, Nielsen T, Qin J, Prifti E, Hildebrand F, Falony G, et al. Richness of human gut microbiome correlates with metabolic markers. Nature. 2013;500 7464:541-6. doi:10.1038/nature12506.
5. Qin J, Li Y, Cai Z, Li S, Zhu J, Zhang F, et al. A metagenome-wide association study of gut microbiota in type 2 diabetes. Nature. 2012;490 7418:55-60. doi:10.1038/nature11450.
6. Karlsson FH, Tremaroli V, Nookaew I, Bergstrom G, Behre CJ, Fagerberg B, et al. Gut metagenome in European women with normal, impaired and diabetic glucose control. Nature. 2013;498 7452:99-103. doi:10.1038/nature12198.
7. Joossens M, Huys G, Cnockaert M, De Preter V, Verbeke K, Rutgeerts P, et al. Dysbiosis of the faecal microbiota in patients with Crohn's disease and their unaffected relatives. Gut. 2011;60 5:631-7. doi:10.1136/gut.2010.223263.
8. Huttenhower C, Kostic Aleksandar D and Xavier Ramnik J. Inflammatory Bowel Disease as a Model for Translating the Microbiome. Immunity. 2014;40 6:843-54. doi:10.1016/j.immuni.2014.05.013.

9. Zhang X, Zhang D, Jia H, Feng Q, Wang D, Liang D, et al. The oral and gut microbiomes are perturbed in rheumatoid arthritis and partly normalized after treatment. Nature medicine. 2015;21 8:895-905. doi:10.1038/nm.3914.

10. Karlsson FH, Fak F, Nookaew I, Tremaroli V, Fagerberg B, Petranovic D, et al. Symptomatic atherosclerosis is associated with an altered gut metagenome. Nature communications. 2012;3:1245. doi:10.1038/ncomms2266.

11. Jie Z, Xia H, Zhong SL, Feng Q, Li S, Liang S, et al. The gut microbiome in atherosclerotic cardiovascular disease. Nature communications. 2017;8 1:845. doi:10.1038/s41467-017-00900-1.

12. Feng Q, Liang S, Jia H, Stadlmayr A, Tang L, Lan Z, et al. Gut microbiome development along the colorectal adenoma-carcinoma sequence. Nature communications. 2015;6:6528. doi:10.1038/ncomms7528.

13. Foster JA and McVey Neufeld KA. Gut-brain axis: how the microbiome influences anxiety and depression. Trends in neurosciences. 2013;36 5:305-12. doi:10.1016/j.tins.2013.01.005.

14. Finegold SM, Dowd SE, Gontcharova V, Liu C, Henley KE, Wolcott RD, et al. Pyrosequencing study of fecal microflora of autistic and control children. Anaerobe. 2010;16 4:444-53. doi:10.1016/j.anaerobe.2010.06.008.

15. Xiao L, Feng Q, Liang S, Sonne SB, Xia Z, Qiu X, et al. A catalog of the mouse gut metagenome. Nature biotechnology. 2015;33 10:1103-8. doi:10.1038/nbt.3353.

16. Lagkouvardos I, Pukall R, Abt B, Foesel BU, Meier-Kolthoff JP, Kumar N, et al. The Mouse Intestinal Bacterial Collection (miBC) provides host-specific insight into cultured diversity and functional potential of the gut microbiota. Nature microbiology. 2016;1 10:16131. doi:10.1038/nmicrobiol.2016.131.

17. Nguyen TL, Vieira-Silva S, Liston A and Raes J. How informative is the mouse for human gut microbiota research? Disease models & mechanisms. 2015;8 1:1-16. doi:10.1242/dmm.017400.

18. Clayton JB, Vangay P, Huang H, Ward T, Hillmann BM, Al-Ghalith GA, et al. Captivity humanizes the primate microbiome. Proc Natl Acad Sci U S A. 2016;113 37:10376-81. doi:10.1073/pnas.1521835113.

19. Angelakis E, Yasir M, Bachar D, Azhar EI, Lagier JC, Bibi F, et al. Gut microbiome and dietary patterns in different Saudi populations and monkeys. Scientific reports. 2016;6:32191. doi:10.1038/srep32191.

20. He X, Slupsky CM, Dekker JW, Haggarty NW and Lönnerdal B. Integrated Role of Bifidobacterium animalis subsp. lactis Supplementation in Gut Microbiota, Immunity, and Metabolism of Infant Rhesus Monkeys. mSystems. 2016;1 doi:10.1128/mSystems.00128-16.

21. Hale VL, Tan CL, Niu K, Yang Y, Knight R, Zhang Q, et al. Diet Versus Phylogeny: a Comparison of Gut Microbiota in Captive Colobine Monkey Species. Microbial ecology. 2017; doi:10.1007/s00248-017-1041-8.

22. Wu GD, Chen J, Hoffmann C, Bittinger K, Chen YY, Keilbaugh SA, et al. Linking long-term dietary patterns with gut microbial enterotypes. Science. 2011;334 6052:105-8. doi:10.1126/science.1208344.

23. R L, B L, Y X, Z L, W H and J Y. SOAPdenovo2 an empirically improved memory-efficient short-read de novo assembler. GigaScience. 2012; doi:10.1186/2047-217X-1-18.

24. Noguchi H, Park J and Takagi T. MetaGene: prokaryotic gene finding from environmental genome shotgun sequences. Nucleic Acids Res. 2006;34 19:5623-30. doi:10.1093/nar/gkl723.
25. Li W and Godzik A. Cd-hit: a fast program for clustering and comparing large sets of protein or nucleotide sequences. Bioinformatics. 2006;22 13:1658-9. doi:10.1093/bioinformatics/btl158.
26. Li R, Yu C, Li Y, Lam TW, Yiu SM, Kristiansen K, et al. SOAP2: an improved ultrafast tool for short read alignment. Bioinformatics. 2009;25 15:1966-7. doi:10.1093/bioinformatics/btp336.
27. Kanehisa M, Sato Y, Kawashima M, Furumichi M and Tanabe M. KEGG as a reference resource for gene and protein annotation. Nucleic Acids Res. 2016;44 D1:D457-62. doi:10.1093/nar/gkv1070.
28. Cantarel BL, Coutinho PM, Rancurel C, Bernard T, Lombard V and Henrissat B. The Carbohydrate-Active EnZymes database (CAZy): an expert resource for Glycogenomics. Nucleic Acids Res. 2009;37 Database issue:D233-8. doi:10.1093/nar/gkn663.
29. Qin J, Li R, Raes J, Arumugam M, Burgdorf KS, Manichanh C, et al. A human gut microbial gene catalogue established by metagenomic sequencing. Nature. 2010;464 7285:59-65. doi:10.1038/nature08821.
30. Gerlach W and Stoye J. Taxonomic classification of metagenomic shotgun sequences with CARMA3. Nucleic Acids Res. 2011;39 14:e91. doi:10.1093/nar/gkr225.
31. Li J, Jia H, Cai X, Zhong H, Feng Q, Sunagawa S, et al. An integrated catalog of reference genes in the human gut microbiome. Nature biotechnology. 2014;32 8:834-41. doi:10.1038/nbt.2942.
32. Xiao L, Estelle J, Kiilerich P, Ramayo-Caldas Y, Xia Z, Feng Q, et al. A reference gene catalogue of the pig gut microbiome. Nat Microbiol. 2016:16161. doi:10.1038/nmicrobiol.2016.161.
33. Koren O, Knights D, Gonzalez A, Waldron L, Segata N, Knight R, et al. A guide to enterotypes across the human body: meta-analysis of microbial community structures in human microbiome datasets. PLoS computational biology. 2013;9 1:e1002863. doi:10.1371/journal.pcbi.1002863.
34. Arumugam M, Raes J, Pelletier E, Le Paslier D, Yamada T, Mende DR, et al. Enterotypes of the human gut microbiome. Nature. 2011;473 7346:174-80. doi:10.1038/nature09944.
35. Zhu L, Baker SS, Gill C, Liu W, Alkhouri R, Baker RD, et al. Characterization of gut microbiomes in nonalcoholic steatohepatitis (NASH) patients: a connection between endogenous alcohol and NASH. Hepatology. 2013;57 2:601-9. doi:10.1002/hep.26093.
36. Ding T and Schloss PD. Dynamics and associations of microbial community types across the human body. Nature. 2014;509 7500:357-60. doi:10.1038/nature13178.
37. Madsen L, Myrmel LS, Fjære E, Liaset B and Kristiansen K. Links between Dietary Protein Sources, the Gut Microbiota, and Obesity. Front Physiol. 2017;8 1047 doi:10.3389/fphys.2017.01047.
38. Wos-Oxley M, Bleich A, Oxley AP, Kahl S, Janus LM, Smoczek A, et al. Comparative evaluation of establishing a human gut microbial community within rodent models. Gut microbes. 2012;3 3:234-49. doi:10.4161/gmic.19934.
39. Turnbaugh PJ, Ridaura VK, Faith JJ, Rey FE, Knight R and Gordon JI. The effect of diet on the human gut microbiome: a metagenomic analysis in humanized gnotobiotic mice. Science translational medicine. 2009;1 6:6ra14. doi:10.1126/scitranslmed.3000322.

40. Zhang L, Bahl MI, Roager HM, Fonvig CE, Hellgren LI, Frandsen HL, et al. Environmental spread of microbes impacts the development of metabolic phenotypes in mice transplanted with microbial communities from humans. The ISME journal. 2017;11 3:676-90. doi:10.1038/ismej.2016.151.
41. Jurtz VI, Villarroel J, Lund O, Voldby Larsen M and Nielsen M. MetaPhinder-Identifying Bacteriophage Sequences in Metagenomic Data Sets. PloS one. 2016;11 9:e0163111. doi:10.1371/journal.pone.0163111.
42. Kapusinszky B, Ardeshir A, Mulvaney U, Deng X and Delwart E. Case-Control Comparison of Enteric Viromes in Captive Rhesus Macaques with Acute or Idiopathic Chronic Diarrhea. J Virol. 2017;91 18 doi:10.1128/JVI.00952-17.
43. Powell S, Forslund K, Szklarczyk D, Trachana K, Roth A, Huerta-Cepas J, et al. eggNOG v4.0: nested orthology inference across 3686 organisms. Nucleic Acids Res. 2014;42 Database issue:D231-9. doi:10.1093/nar/gkt1253.
44. Chao A. Estimating the population size for capture-recapture data with unequal catchability. Biometrics. 1987;43 4:783-91.

Figure legend

Figure 1. Rarefaction curve based on gene numbers and taxonomic annotation of the cynomolgus macaques gene catalog.

a. Rarefaction curve based on the gene numbers of all cynomolgus macaques samples and the individual subgroups.

b. Taxonomic annotation of 1.9M cynomolgus macaques gene catalog. More than 65% of the genes from cynomolgus macaques gene catalog could be annotated to the bacterial superkingdom. 13.91% of the genes could be annotated to the genus level.

Figure 2. Characteristic of the cynomolgus macaques gut microbiota.

a. The top 10 phyla in cynomolgus macaques gut microbiota. *Bacteroidetes* and *Firmicutes* are the main two phyla in the cynomolgus macaques gut microbiota.

b. The top 20 genera in the cynomolgus macaques gut microbiota. *Prevotella* is the main genus in the cynomolgus macaques gut microbiota.

c. Genus network analysis of core genera in the cynomolgus macaques gut microbiota reveals significant interactions. The size of the node is proportional to the genus abundance. Node color corresponds to phylum taxonomic classification. Edge color represents positive (green) and negative (pink) correlations, and the edge thickness is equivalent to the correlation values.

Figure 3. Comparison with the human, mouse and pig gut microbiomes.

a. Unique non-redundant genes in the cynomolgus macaques, human, pig and mouse gut gene catalog. Less than 0.4% genes overlapped between all the four species, which emphasizes the marked differences between the cynomolgus macaques, human, pig and mouse gut microbiome at the gene level.

b. Comparison of the cynomolgus macaques, human, pig and mouse microbiotas based on KEGG annotation, which emphasizes the functional similarity between the cynomolgus macaques, human, pig and mouse gut microbiota despite the marked differences at the gene level shown in a.

c. PCA analysis based on overlapping KOs of the cynomolgus macaques, human, mouse, and pig gut microbiota.

d. The top 20 core genera in the cynomolgus macaques, human, pig and mouse gut microbiota. The 10 shared genera are marked in red.

Figure 4. Diet-related differences in the cynomolgus macaques gut microbiota.

a. PCA analysis of cynomolgus macaques samples based on gene profiles.

b. KEGG functional classification of the 82,120 gene makers. The black bars represent the total percentage in the 1.9M gene catalog. The gray bars represent gene markers enriched in high-fat/low fiber diet group. The white bars represent gene marker rate enriched in the low-fat/high fiber control group.

Additional files

Additional file 1: Data production from cynomolgus macaques gut samples.

Additional file 2: KEGG pathway classification and CAZy classification.

a. KEGG pathway classification. 53.09% of the cynomolgus macaques gene catalog could be annotated to the KO level.

b. CAZy classification. 3.41% of the cynomolgus macaques gene catalog could be annotated in the CAZy database.

Additional file 3: The average abundance of the 80 core genera shared among all cynomolgus macaques individuals.

Additional file 4: The enterotype-like cluster in the cynomolgus macaques, mouse and pig samples.

a. Enterotype-like clusters in the cynomolgus macaques samples.

b. Abundances of the main contributors to each enterotype-like cluster in the cynomolgus macaques samples.

c. Enterotype-like clusters in the mouse samples.

d. Abundances of the main contributors to each enterotype-like cluster in mouse samples.

e. Enterotype-like clusters in the pig samples.

f. Abundances of the main contributors to each enterotype-like cluster in the pig samples.

Additional file 5: Mapping ratio of cynomolgus macaques, human, pig and mouse.

a. Average mapping ratio of cynomolgus macaques sample reads to 1 million genes randomly selected (10 times) from the cynomolgus macaques, human, pig and mouse gene catalogs.

b. Average mapping ratio of 20 samples from mouse, pig, cynomolgus macaques, and human mapped to 9.9M human gut gene catalogs.

Additional file 6: Alpha diversity

a. The alpha diversity of cynomolgus macaques gut microbiota comparing to human pig and mouse gut microbiota. The alpha diversity of pig is highest compared to the gut microbiota of the other three species, and human is lowest.

b. The alpha diversity of cynomolgus macaques gut microbiome in samples form animals fed the low-fat/high fiber diet or the high-fat/low fiber diet, with the latter showing the lowest alpha diversity.

Additional file 7: Core genera in cynomolgus macaques, pig, human and mouse.

a. Venn diagram of core genera in the cynomolgus macaques, pig, human and mouse; b: heatmap of the 32 mammalian core genera.

Additional file 8: Phenotypic information of all cynomolgus macaques individuals.

Additional file 9: KEGG module enrichment in the gut microbiotas of animal fed the low-fat/high fiber diet and the high-fat low fiber diet.

Additional file 10: KEGG pathway enrichment in cynomolgus macaques fed the high-fat/low fiber or the low-fat/high fiber diets.

Additional file 11: List of predicted phage genes that differ significantly in abundance between the high-fat/low fiber diet and low-fat/high fiber diet fed cynomolgus macaques groups.

Additional file 12: Heatmap of the abundance of predicted phage genes that differ significantly in abundance between the high-fat/low fiber diet and low-fat/high fiber diet fed cynomolgus macaques groups.

We selected phage genes with zero abundance in all the low-fat/high fiber diet fed cynomolgus macaques individuals and exhibited non-zero abundance in all the high-fat/low fiber diet fed cynomolgus macaques individuals and vice versa, i.e. zero abundance in all the high-fat/low fiber diet fed cynomolgus macaques individuals and non-zero abundance in all

the low-fat/high fiber diet fed cynomolgus macaques individuals

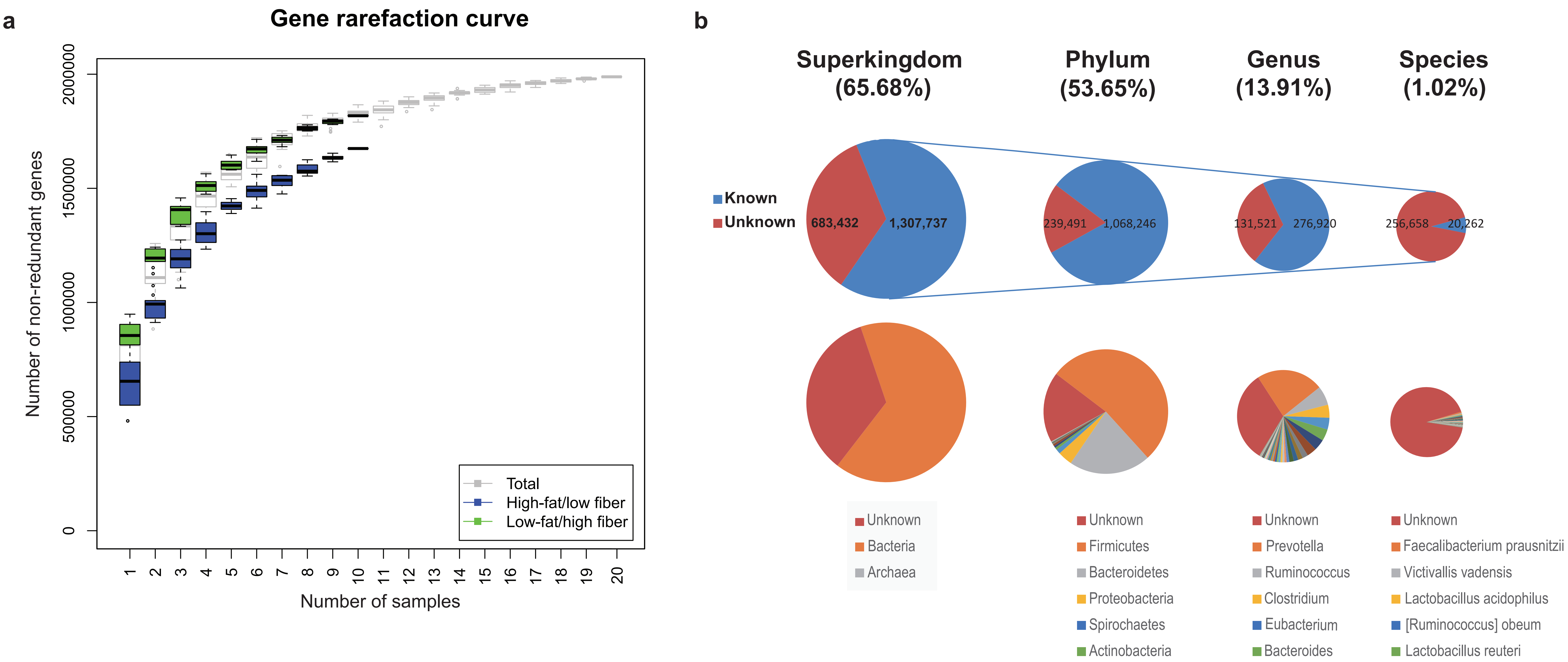
a
Gene rarefaction curve
Number of non-redundant genes
0
500000
1000000
1500000
2000000
Number of samples
1
2
3
4
5
6
7
8
9
10
11
12
13
14
15
16
17
18
19
20
Total
High-fat/low fiber
Low-fat/high fiber
b
Superkingdom (65.68%)
Phylum (53.65%)
Genus (13.91%)
Species (1.02%)
Known
Unknown
683,432
1,307,737
239,491
1,068,246
131,521
276,920
256,658
20,262
Unknown
Bacteria
Archaea
Unknown
Firmicutes
Bacteroidetes
Proteobacteria
Spirochaetes
Actinobacteria
Unknown
Prevotella
Ruminococcus
Clostridium
Eubacterium
Bacteroides
Unknown
Faecalibacterium prausnitzii
Victivallis vadensis
Lactobacillus acidophilus
[Ruminococcus] obeum
Lactobacillus reuteri

Figure
Click here to download Figure Figure 2.pdf.pdf

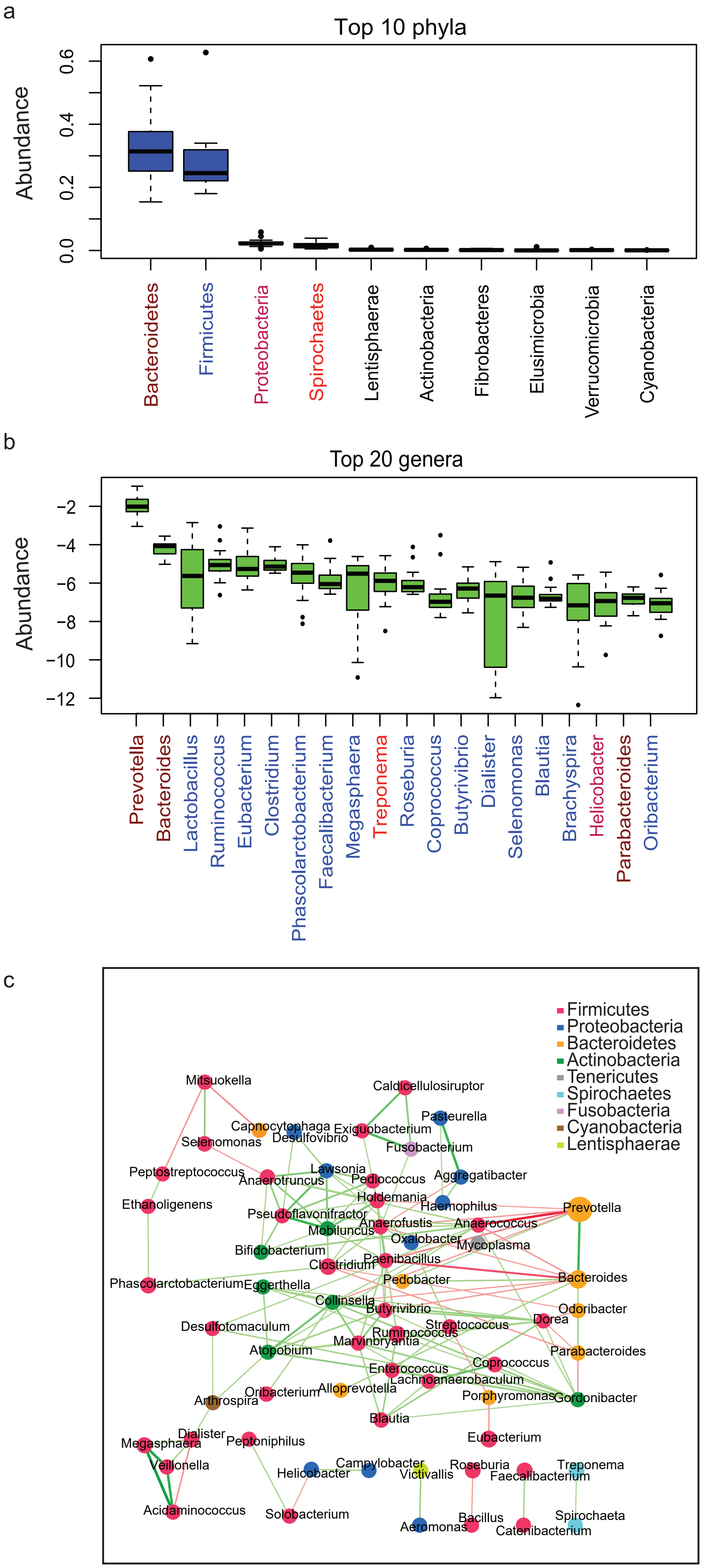

Figure

Click here to download Figure Figure 3pdf.pdf

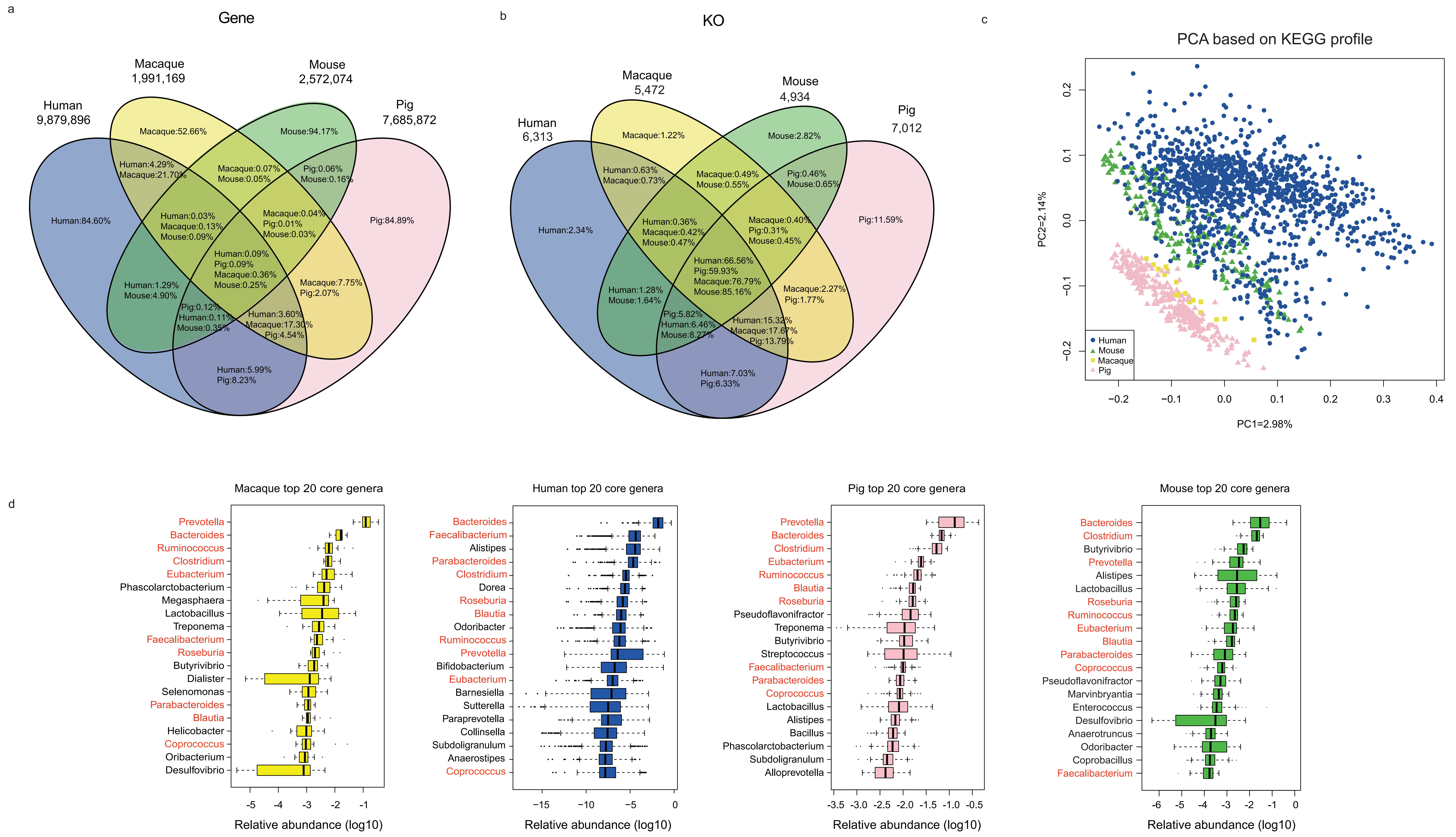

Figure

Click here to download Figure Figure 4pdf.pdf

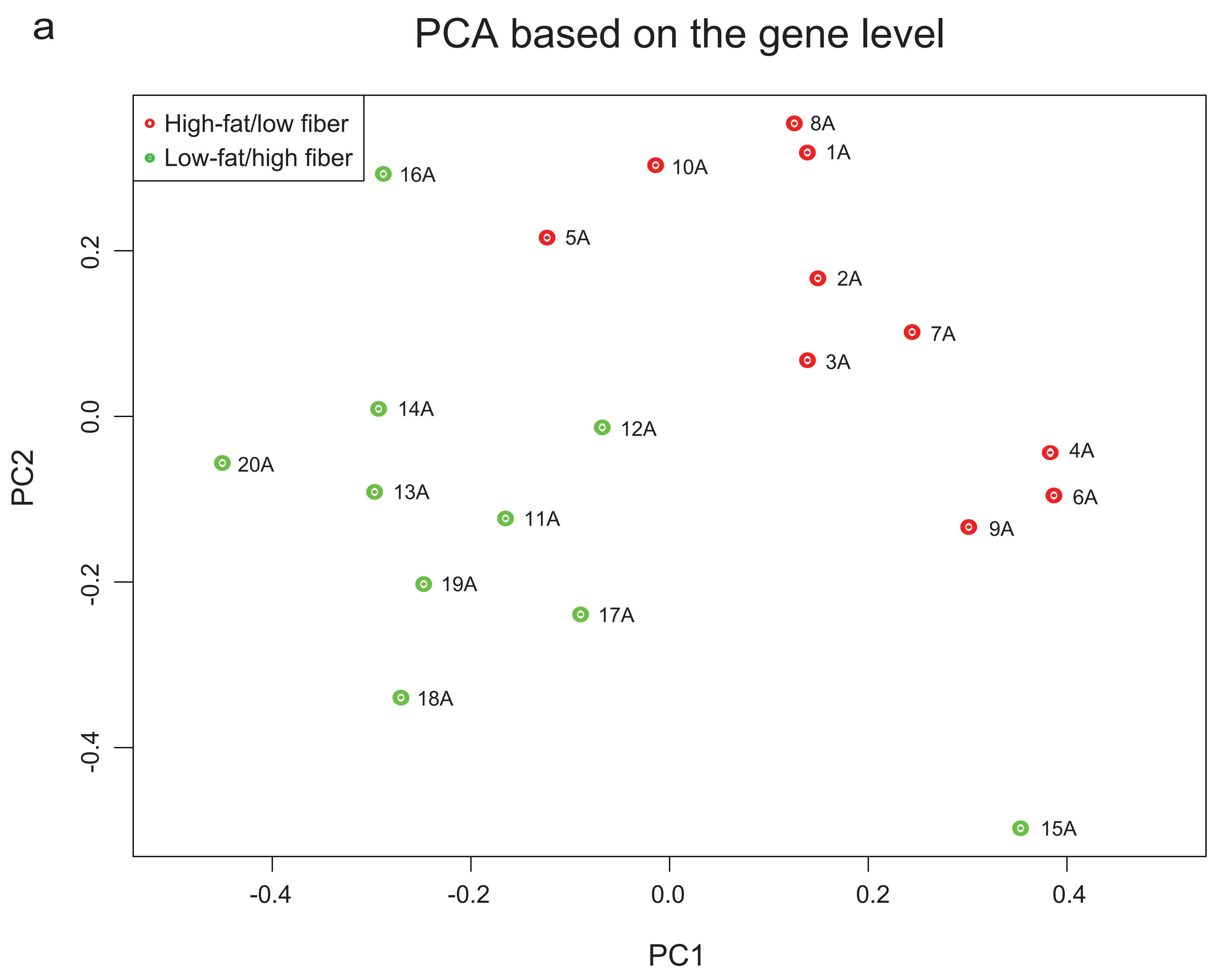

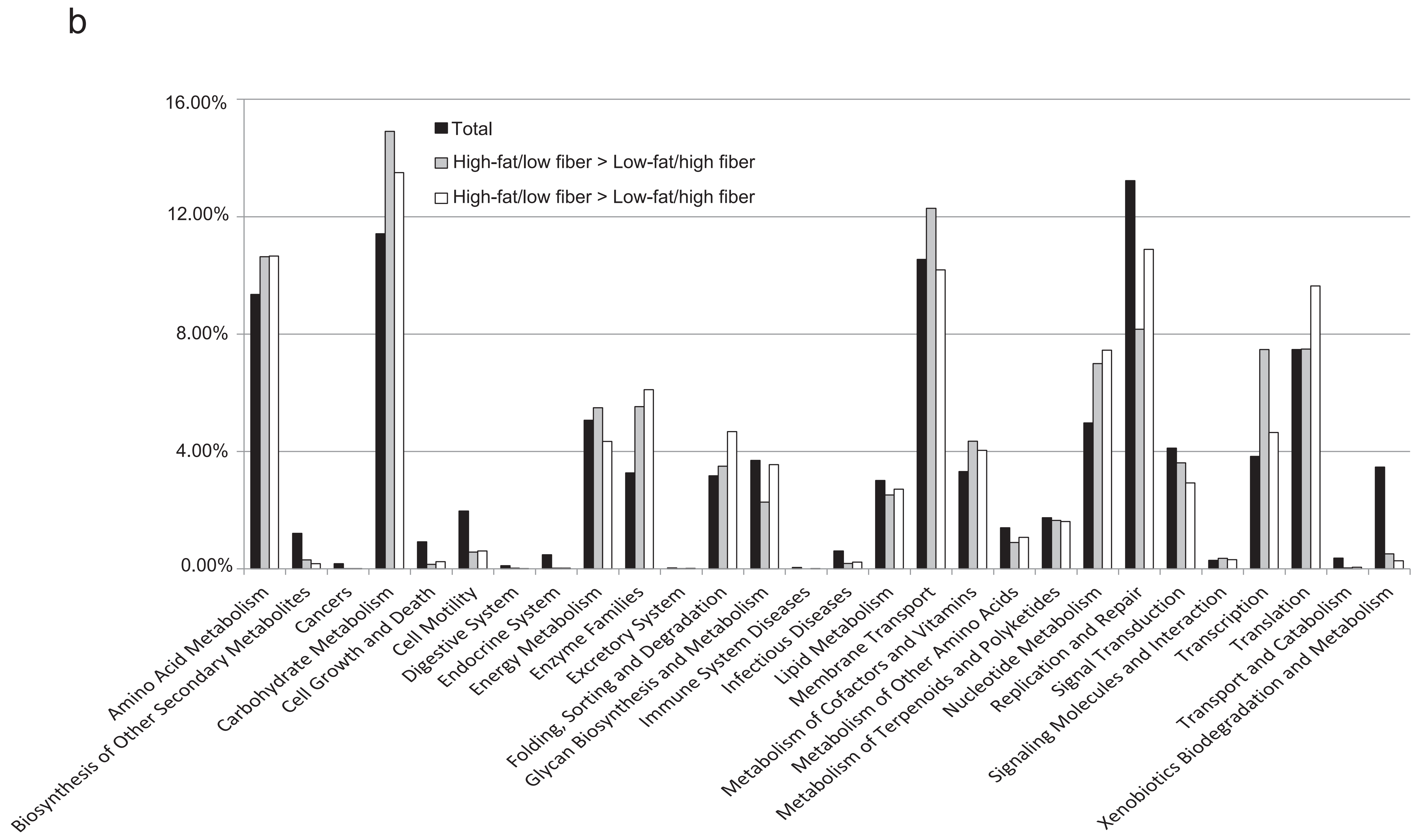

Click here to access/download
Supplementary Material
Additional file 1.xlsx.xlsx

Click here to access/download
Supplementary Material
Additional file 2.pdf.pdf

Click here to access/download
Supplementary Material
Additional file 3.xlsx.xlsx

Click here to access/download
Supplementary Material
Additional file 4.pdf.pdf

Click here to access/download
Supplementary Material
Additional file 5.pdf.pdf

Click here to access/download
Supplementary Material
Additional file 6.pdf.pdf

Click here to access/download
Supplementary Material
Additional file 7.pdf.pdf

Click here to access/download
Supplementary Material
Additional file 8.xlsx.xlsx

Click here to access/download
Supplementary Material
Additional file 9.xlsx.xlsx

Click here to access/download
Supplementary Material
Additional file 10.xlsx.xlsx

Click here to access/download
Supplementary Material
Additional file 11.xls.xlsx

Click here to access/download
Supplementary Material
Additional file 12.pdf.pdf

Personal Cover Click here to download Personal Cover Cover letter-Final.docx

Dear Editor:

We hereby submit a manuscript entitled "Establishment of a *Macaca fascicularis* gut microbiome gene catalog and comparison with the human, pig and mouse gut microbiomes" by Li et al., which we hope will be considered for publication as a Research paper in GigaScience.

In this study, we established the gut microbial gene catalog of *Macaca fascicularis*, the cynomolgus macaques, a species of considerable importance for biomedical research and drug development. This is the first gut microbial gene catalogue of a nonhuman primate (NHP), and will serve as a reference resource for further metagenomic studies. We also compared the gut microbial gene catalog of the cynomolgus macaques with those of human, pig and mouse, showing that, the cynomolgus macaques gut microbiome is more similar to the human microbiome. A set of core gut bacterial genera was defined based on these four mammalian species. Besides, the significant differences of cynomolgus macaque gut microbiome between individuals under high-fat/low fiber diet and low-fat/high fiber diet were explored at the gene and functional levels.

Our report focuses on the following topics:

1) Construction of a gene catalog and features of the cynomolgus macaques gut microbiome: fresh feces were sampled from 20 cynomolgus macaques fed either high-fat/low fiber or low-fat/high fiber diets. By metagenomic sequencing we constructed a gut microbial gene catalog comprising 1,991,169 non-redundant genes.

2) Comparison of the cynomolgus macaques, pig, human and mouse gut gene catalogs: the overlap between the cynomolgus macaques, pig, human and mouse gut microbiome gene catalogs is less than 0.4% at the gene level but more than 76% at the KO functional level. The reads from cynomolgus macaque individuals showed significantly higher mapping rate to the human gut gene catalog than the pig and mouse gene set. This shows that the gut microbiome of cynomolgus macaques is more similar to the human gut microbiome than to those of other analyzed mammalian species. Indeed, our results provide gut microbiome data to assess biological similarities between the cynomolgus macaques and human, and will contribute to further evaluation of the cynomolgus macaques microbiome as a model for its human counterpart.

3) Differences of the cynomolgus macaques gut microbiome under high-fat/low fiber diet fed and low-fat/high fiber diet fed conditions: The genes and KOs differed significantly in abundance between the high-fat/low fiber diet and low-fat/high fiber diet fed groups. Interestingly, a subset of predicted bacterial phage genes differed in abundance between the two groups. These results demonstrated that dietary intake with different content of fat and fiber elicited pronounced differences in the gut microbiome of the cynomolgus macaques.

We hope that you find our manuscript of sufficient quality and general interest to be considered for publication in GigaScience.

On behalf of the authors

Yours sincerely,

Liang Xiao
