# Supplementary material for: Establishment of a Macaca fascicularis gut microbiome gene catalog and comparison with the human, pig, and mouse gut microbiomes
Source: Gigascience. 2018 Aug 18;7(9):giy100. doi: 10.1093/gigascience/giy100 (PMC6137240; doi:10.1093/gigascience/giy100)
Supplement: GIGA-D-17-00351_Revsion_2.pdf [file giy100_giga-d-17-00351_revsion_2.pdf]

GigaScience

Establishment of a Macaca fascicularis gut microbiome gene catalog and comparison with the human, pig and mouse gut microbiomes

--Manuscript Draft--

**Manuscript Number:**	GIGA-D-17-00351R2
**Full Title:**	Establishment of a Macaca fascicularis gut microbiome gene catalog and comparison with the human, pig and mouse gut microbiomes
**Article Type:**	Research
**Funding Information:**	Shenzhen Municipal Government of China (JSGG20160229172752028, JCYJ20160229172757249) — Not applicable<br>National Natural Science Foundation of China (81670606, 81673850) — Not applicable
**Abstract:**	Macaca fascicularis, the cynomolgus macaque, is a widely used model in biomedical research and drug development as its genetics and physiology are close to humans. Detailed information on the cynomolgus macaque gut microbiota, the functional interplay between the gut microbiota and host physiology, and possible similarities to humans and other mammalians is very limited. The aim of this study was to construct the first cynomolgus macaque gut microbial gene catalog and compare this catalog to the human, pig, and mouse gut microbial gene catalogs. We performed metagenomic sequencing on fecal samples from twenty cynomolgus macaques and identified in total 1.9 million non-redundant bacterial genes of which 39.49% and 25.45% are present in the human and pig gut bacterial gene catalogs, respectively, whereas only 0.6% of the genes are present in the mouse gut bacterial gene catalog. By contrast, at the functional levels, more than 76% KOs are shared between the gut microbiota of all four mammalians. Thirty-two highly abundant bacterial genera could be defined as core genera of these mammalians. We demonstrated significant differences in the composition and functional potential of the gut microbiota as well as in the distribution of predicted bacterial phages sequences in cynomolgus macaques fed either a low-fat/high fiber diet or a high-fat/low fiber diet. Interestingly, the gut microbiota of cynomolgus macaques fed the high-fat/low fiber diet became more similar to the gut microbiota of humans.
**Corresponding Author:**	Liang Xiao<br><br>CHINA
**Corresponding Author Secondary Information:**	
**Corresponding Author's Institution:**	
**Corresponding Author's Secondary Institution:**	
**First Author:**	Xiaoping Li
**First Author Secondary Information:**	
**Order of Authors:**	Xiaoping Li
	Suisha Liang
	Zhongkui Xia
	Jing Qu
	Huan Liu
	Chuan Liu
	Huanming Yang
	Jian Wang

Powered by Editorial Manager® and ProduXion Manager® from Aries Systems Corporation

	Lise Madsen
	Yong Hou
	Junhua Li
	Huijue Jia
	Karsten Kristiansen
	Liang Xiao
**Order of Authors Secondary Information:**	
**Response to Reviewers:**	We are very pleased by the positive comments from the reviewers and the acceptance of our manuscript for publication in GigaScience. We have made minor corrections of grammar and further adjusted the text as required by reviewer 2. See the supplemental file for a more detailed point-by-point response.
**Additional Information:**	
**Question**	**Response**
Are you submitting this manuscript to a special series or article collection?	No
**Experimental design and statistics**<br><br>Full details of the experimental design and statistical methods used should be given in the Methods section, as detailed in our Minimum Standards Reporting Checklist. Information essential to interpreting the data presented should be made available in the figure legends.<br><br>Have you included all the information requested in your manuscript?	Yes
**Resources**<br><br>A description of all resources used, including antibodies, cell lines, animals and software tools, with enough information to allow them to be uniquely identified, should be included in the Methods section. Authors are strongly encouraged to cite Research Resource Identifiers (RRIDs) for antibodies, model organisms and tools, where possible.<br><br>Have you included the information requested as detailed in our Minimum Standards Reporting Checklist?	Yes

Powered by Editorial Manager® and ProduXion Manager® from Aries Systems Corporation

**Availability of data and materials**<br><br>All datasets and code on which the conclusions of the paper rely must be either included in your submission or deposited in publicly available repositories (where available and ethically appropriate), referencing such data using a unique identifier in the references and in the "Availability of Data and Materials" section of your manuscript.<br><br>Have you have met the above requirement as detailed in our Minimum Standards Reporting Checklist?	Yes

Powered by Editorial Manager® and ProduXion Manager® from Aries Systems Corporation

Manuscript
Click here to view linked References
Click here to access/download;Manuscript;Revised Manuscript-clean-20180705(1).docx

Establishment of a *Macaca fascicularis* gut microbiome gene catalog and comparison with the human, pig and mouse gut microbiomes

Xiaoping Li[1,2,3,#], Suisha Liang[1,2,3,#], Zhongkui Xia[1,2,3], Jing Qu[1,2,6], Huan Liu[1,2], Chuan Liu[1,2,3], Huanming Yang[1,2,4], Jian Wang[1,2,4], Lise Madsen[1,7,8], Yong Hou[1,2], Junhua Li[1,2,3,5], Huijue Jia[1,2,3], Karsten Kristiansen[1,2,7*], Liang Xiao[1,2*]

1 BGI-Shenzhen, Shenzhen 518083, China,

2 China National GeneBank, BGI-Shenzhen, Shenzhen 518120, China,

3 Shenzhen Key Laboratory of Human Commensal Microorganisms and Health Research, BGI-Shenzhen, Shenzhen 518083, China,

4 James D. Watson Institute of Genome Sciences, Hangzhou 310058, China,

5 School of Bioscience and Biotechnology, South China University of Technology, Guangzhou, 510006, China

6 BGI Innovation College of QingDao University, Qingdao, 266071, China

7 Laboratory of Genomics and Molecular Biomedicine, Department of Biology, University of Copenhagen, 2100 Copenhagen Ø, Denmark

8 Institute of Marine Research (IMR), Postboks 1870, Nordnes, N-5817, Bergen, Norway.

ORCID IDs: Xiaoping Li: 0000-0002-6339-0507; Zhongkui Xia: 0000-0003-3786-9394; Jing Qu: 0000-0002-3724-9737; Huan Liu: 0000-0003-3909-0931; Lise Madsen: 0000-0003-4468-1947; Junhua Li: 0000-0001-6784-1873; Huijue Jia: 0000-0002-3592-126X

# These authors contributed equally to this work

* Corresponding authors

Abstract

Macaca fascicularis, the cynomolgus macaque, is a widely used model in biomedical research and drug development as its genetics and physiology are close to humans. Detailed information on the cynomolgus macaque gut microbiota, the functional interplay between the gut microbiota and host physiology, and possible similarities to humans and other mammalians is very limited. The aim of this study was to construct the first cynomolgus macaque gut microbial gene catalog and compare this catalog to the human, pig, and mouse gut microbial gene catalogs. We performed metagenomic sequencing on fecal samples from twenty cynomolgus macaques and identified in total 1.9 million non-redundant bacterial genes of which 39.49% and 25.45% are present in the human and pig gut bacterial gene catalogs, respectively, whereas only 0.6% of the genes are present in the mouse gut bacterial gene catalog. By contrast, at the functional levels, more than 76% KOs are shared between the gut microbiota of all four mammalians. Thirty-two highly abundant bacterial genera could be defined as core genera of these mammalians. We demonstrated significant differences in the composition and functional potential of the gut microbiota as well as in the distribution of predicted bacterial phages sequences in cynomolgus macaques fed either a low-fat/high fiber diet or a high-fat/low fiber diet. Interestingly, the gut microbiota of cynomolgus macaques fed the high-fat/low fiber diet became more similar to the gut microbiota of humans.

Keywords: *Macaca fascicularis*, gut microbiota gene catalog, gut microbiome, core genera, high-fat/low fiber diet, low-fat/high fiber diet, metagenomics

Background

The intestine is home to trillions of bacteria, which in number equal or even outnumber the number of host cells [1]. Accumulating evidence points to a link between the gut microbiota and several common diseases, including obesity [2-4], diabetes [5, 6], Crohn's disease [7], ulcerative colitis [8], rheumatoid diseases [9], cardiovascular disease (CVD) [10, 11], and colorectal cancer [12]. Recent evidence also links changes in the gut microbiota to certain mental disorders [13, 14].

In order to establish causality between a given alteration of the gut microbiota and disease, rodent models are most frequently used. Previous studies have clearly demonstrated that the mouse gut microbiome is very different that of humans [15-17]. Non-human primates (NHPs) are seemingly more biologically relevant animal models for humans, but very little information on their microbiomes is available. In captivity, *Macaca fascicularis*, the cynomolgus macaque, has been reported to have undergone a loss of native microbes, and the primary bacterial genera in gut were reported to be *Prevotella* and *Bacteroides*, similar to dominant genera in the human gut [18, 19]. Thus, detailed studies on the composition and functional capacity of the gut microbiota of the cynomolgus macaque are warranted in order to examine the potential of this model for biomedical research.

Previous studies have explored the gut microbiota of different monkey species using 16S rRNA gene amplicon sequencing providing little information on gene identity and function of the monkey gut microbiome [18-21]. In the present study, fecal samples from twenty cynomolgus macaques were used for metagenomics sequencing resulting in the generation of

a catalog comprising 1.9M non-redundant bacterial genes. Comparison of the human, pig, mouse and cynomolgus macaque gut microbiomes demonstrated that the cynomolgus macaque gut microbiome is more similar to that of human than those of pig and mouse at the gene level. We observed that the gut microbiota of cynomolgus macaques fed either a low-fat/high fiber diet or a high-fat/low fiber diet exhibited differences in composition and functional potential, which to a certain degree mimicked those observed in humans shifted between intake of a low-fat/high fiber diet and a high-fat/low fiber diet [22]. We envisage that the present gut bacterial gene catalog and the functional characterization will serve as a valuable reference and resource for biomedical research using the cynomolgus macaque as a model.

Data Description

To establish a *Macaca fascicularis*, the cynomolgus macaque, gut microbial gene catalog, fecal samples from 20 cynomolgus macaque individuals were collected. The animals were divided into two groups and fed either a low-fat/high fiber diet or a high-fat/low fiber diet for three months. Further details are given in Methods. Total DNA was extracted from freshly collected fecal samples from all animals and used for sequencing on the Illumina HiSeq2000 platform as described previously [1]. In total, 140 gigabases (Gb) data were generated with an average of 7Gb per sample (additional file 1). The raw data were filtered with a quality control cutoff (adapter sequence <15bp, 'N' base <3bp, Q>20, final length >30) and host sequences were removed by alignment against the *M. fascicularis* genome (NCBI accession no. NC_022272.1 - NC_022292.1), resulting in 131 Gb clean data used for assembly and

open reading frames (ORFs) prediction using SOAPdenovo [23] and Metagene2 [24], respectively. Redundant ORFs from each sample were removed by CD-HIT [25], providing a 1.9M non-redundant cynomolgus macaque gut microbial gene catalog. The gene profiles were generated by mapping clean data to the gene catalog with soap2.22 [26]. The genes in the catalog were aligned against the NCBI-NR, the Kyoto Encyclopedia of Genes and Genomes (KEGG) [27] and the carbohydrate-active enzymes (CAZy) [28] database to obtain taxonomic and functional annotation.

Analyses

Construction of cynomolgus macaque gut bacterial gene catalog

De novo assembly, gene prediction, and elimination of redundant genes were performed as previously described [29] generating a non-redundant (NR) gene set comprising 1,991,169 open reading frames (ORFs) with an average length of 757 base pairs (bp).

A rarefaction analysis based on gene number revealed a curve approaching saturation with 15 samples, and incidence-based coverage estimator, Chao1 indices, further indicated that we captured 97.00% of the gut microbial genes in the samples (Fig 1a).

We could taxonomically classify 65.68% of the NR genes with CARMA3 [30]. More than 99.99% of the annotated genes could be assigned to the bacteria super kingdom. Of these

genes, 1,068,246 (53.65%) could be annotated to the phylum level. At the phylum level, 52.94% of the annotated genes could be annotated to Firmicutes and 21.25% of the genes could be annotated to Bacteroidetes. At the genus and the species level, 276,920 (13.91%) and 20,262 (1.02%) of the macaque gut bacterial genes could be annotated to the genus and the species level, respectively (Fig 1b). At the genus level, most of the annotated genes (34.55%) belonged to *Prevotella,* followed by *Ruminococcus* (9.91%), *Clostridium* (6.73%), *Eubacterium* (6.12%) and *Bacteroides* (6.00%) (Fig.1b). We also mapped the cynomolgus macaque gene catalog to the Kyoto Encyclopedia of Genes and Genomes (KEGG) database [27]. We could map 1,057,148 (53.09%) genes to KEGG orthology (KO) levels of which 775,931 (38.97%) genes had pathway information. Pathways related to genetic information processing (replication and repair and translation), metabolism (carbohydrates, amino acids, energy and nucleotides) and environmental information processing (membrane transport) (additional file 2a) dominated. Additionally, we mapped the cynomolgus macaque gut bacterial gene catalog to the CAZy database. We were able to map 67,995 (3.41%) of the cynomolgus macaque gut bacterial genes to 248 CAZy families (additional file 2b).

The characteristics of cynomolgus macaque gut microbiome

Based on the taxonomical annotation, Bacteroidetes and Firmicutes were the two main phyla (Fig 2a) and *Prevotella* and *Bacteroides* were the dominant genera (Fig 2b) in the cynomolgus macaque gut microbiota. We found 80 core genera that were shared among all individuals with a lowest average abundance higher than 2.04e-07 (additional file 3).

We identified three enterotypes-like clusters in these 20 individual cynomolgus macaque samples, primarily driven by the highly abundant genera *Prevotella, Lactobacillus and Ruminococcus* (additional file 4a and 4b).

Comparison with the human, mouse and pig gut microbiomes

The cynomolgus macaque gut bacterial catalog was compared with the human [31], pig [32] and the mouse [15] catalog. The human gut gene catalog includes 9,879,896 genes, the pig gut gene catalog 7,685,872 genes and the mouse gut gene catalog 2,572,074 genes (additional file 5). In the cynomolgus macaque gut bacterial gene catalog, 39.49% of the genes are included in the human gut bacterial gene catalog, 25.45% of the genes are present in the pig gut bacterial gene catalog, whereas only 0.6% of the genes are found in the mouse gut gene catalog. Moreover, less than 0.4% of cynomolgus macaque gut genes are shared by these four species, underscoring the marked differences between the gut microbiomes of these mammalian species at the gene level (Fig 3a).

We randomly picked 1 million genes 10 times from the human, pig and mouse gene catalog, respectively, and then mapped the high quality reads generated from the cynomolgus macaque samples to these selections. The mapping rates to the human and pig microbial gene catalogs were 6.26% and 5.30%, respectively, whereas the mapping rate to the mouse catalog was only 0.51% (additional file 6a, *P* value=5.07e-09 in human vs pig). Additionally, high quality reads from 20 samples of pig and mouse were also mapped to the 9.9M human gene catalog. More

reads of cynomolgus macaque gut microbiome (39.23%) could be mapped to the human gene catalog compared to reads from the pig (26.98%) and mouse (16.01%) (additional file 6b). The pig gut microbiota exhibited a higher alpha diversity (additional file 7a) than human, cynomolgus macaque, and mouse microbiomes.

At the functional level, 53.09% of the macaque and 48.77% of the mouse gut genes can be assigned to KOs, 42.10% of the human gut genes can be assigned to KOs, whereas about 35.79% of the pig gut genes can be assigned to KOs. The similarity of annotated KOs between the cynomolgus macaque, human, pig and mouse gut microbiotas is very high (Fig 3b). We identified 4,202 KOs involved in membrane transport and carbohydrate metabolism that are shared between the cynomolgus macaque, human, pig and mouse gut microbiomes. Although the percentage of common KOs (82.87%) shared between human and cynomolgus macaque is less than the percentage shared between human and pig (95.37%), a PCA (Principal component analysis) showed that the cynomolgus macaque gut microbiome is closer to the human than the pig microbiome (Fig 3c). The distribution of CAZy classes was very similar between these four mammalian gut microbiomes (additional file 2b)

We also identified bacterial genera that occurred in all samples from each of these four mammals. We term these core genera and identified 80 such core bacterial genera in the cynomolgus macaque (20 samples), 44 in human (1267 samples) [31], 86 in pig (287 samples) [32], and 60 in mouse (184 samples) [15]. Comparing the core genera from the cynomolgus macaque, human, pig and mouse, we found 32 genera that are shared between all four

mammals (additional file 8a), but we also noted that the abundance of these genera differed between each host (additional file 8b). Amongst the 20 most abundant genera in each species, 10 genera are shared. These included *Prevotella*, *Bacteroides*, *Clostridium*, *Eubacterium*, *Parabacteroides*, *Ruminococcus*, *Faecalibacterium*, *Roseburia*, *Blautia,* and *Coprococcus* which may constitute a core mammalian gut microbiota (Fig 3d).

We compared the enterotype-like clusters of the cynomolgus macaque, the mouse and the pig to human. In the human gut microbiota enterotype-like clusters have been reported to be driven by *Bacteroides*, *Prevotella*, and *Ruminococcus* [12, 22, 33-35], and in some cases *Bifidobacterium* [5], *Alistipe*s and *Faecalibacterium* [36]. In the cynomolgus macaque we found that the enterotype-like clusters were driven by *Lactobacillus*, *Prevotella* and *Ruminococcus*. In the mouse, the enterotype-like clusters were driven by *Alistipes*, *Akkermansia* and *Clostridium*, and finally, in the pig we observed that enterotype-like clusters were driven by *Streptococcus*, *Prevotella* and *Lactobacillus* (additional file 4). Based on the networks of the 32 core genera of these four mammals (additional file 9 and additional file 10), we also analyzed the relationship of these enterotype-representative genera with other genera. We found that *Prevotella* correlated negatively with *Bacteroides* in human gut microbiota, but in cynomolgus macaque and pig microbiotas, *Prevotella* correlated positively with *Bacteroides*. Additionally, in the human and cynomolgus macaque gut microbiotas, *Ruminococcus* correlated positively with both *Blautia* and *Dorea.* Differences in enterotypes in humans have been linked to dietary patterns [22, 37]. However, to what extent the different patterns of enterotype-like clusters in these four species reflect differences in diets and/or

genetics remains to be established. The finding that colonization by human microbiotas in germ free mice is only partial indicates that genetics may play a role [38-40].

Diet-related changes in the cynomolgus macaque gut microbiota

Comparison of cynomolgus macaques fed the low-fat/high fiber or the high-fat/low fiber diets for 3 months revealed that the latter group on average had slightly higher body mass (Wilcoxon rank sum test, P value<0.05) and elevated fasting blood glucose (Wilcoxon rank sum test, P value<0.05) (additional file 11). Notably, the reads from cynomolgus macaque individuals that had consumed the high-fat diet/low fiber diet showed significantly higher mapping rate to the human and the pig gene sets (P value=2.06e-04 in human and P value=3.25e-04 in pig), but not to the mouse gene sets (P value=0.14). In response to these diets, we observed changes of alpha diversity. Intake of the high-fat/low fiber diet tended to decrease alpha diversity, but the difference did not reach statistical significance (P value=0.14) (additional file 7b). However, individuals fed the high-fat/low fiber diet could be clearly distinguished from the control group at the gene level (Fig 4a). In total, we found that 82,120 gene markers differed in abundance comparing the two groups (P value<0.01). Most of these marker genes are involved in metabolism of carbohydrates, amino acids, nucleotides and vitamins. Analysis of genera that differed significantly in abundance between the two groups of cynomolgus macaques was performed (Wilcoxon rank sum test, P value<0.05). We found five genera including *Parabacteroides* and *Succinatimonas* being enriched in individuals fed the high-fat/low fiber diet, whereas in the gut microbiota of individuals fed the low-fat/high

fiber diet, 11 genera including *Ruminococcus, Roseburia, Eubacterium* were enriched (additional file 12). KOs involved in carbohydrate metabolism, energy metabolism, membrane transport, and transcription were more abundant in individuals fed the high-fat/low fiber diet compared to the low-fat/high fiber diet (Fig 4b). At the module or pathway levels, the gut microbiota of high fat/low fiber diet fed cynomolgus macaques was functionally enriched in saccharide, polyol, and lipid transport systems, phosphate and amino acid transport systems and metabolic modules involved in branched-chain amino acid, carbohydrate, lipid, and methane metabolism. The gut microbiota of cynomolgus macaques fed a low-fat/high fiber diet was functionally enriched in bacterial secretion system, protein export, purine metabolism and lipopolysaccharide biosynthesis (additional file 13 and additional file 14). Since the two diets differ both in fat and fiber content, the observed changes most likely reflect changes in both of these constituents. Differences in the composition and functional potential of the gut microbiota in response to a low-fat/high fiber diet or a high-fat/low fiber diet have also been reported in a human study [22]. We observed that some of the KEGG pathways that differed in abundance in the human study in response to the different diet, including bacterial secretion system and protein export, also differed in response to the two diets in cynomolgus macaques.

The distribution of predicted phage sequences in gut microbiome of cynomolgus macaques

In total 311,017 (15.62%) of the genes in the cynomolgus macaque gut gene catalog were

predicted as bacterial phage sequences by Metafinder [41] (ANI >1.7%). Similar ratios of phage genes in human, mouse and pig gut gene catalog were also predicted using the same pipeline (additional file 15). By comparing the distribution of these predicted phage genes between cynomolgus macaques fed the high-fat/low fiber diet and low-fat/high fiber diet, 56,800 gene were found to differ significantly in abundance between the two groups (Wilcoxon rank sum test, $P<0.05$) (additional file 16). Of these, 43,602 were enriched in the control group while 13,198 genes were enriched in macaques fed the high-fat/low fiber diet. Additionally, the heat map clearly separated these genes between the two diet groups (additional file 17).

Discussion

Here we constructed a gut bacterial gene catalog of *M. fascicularis*, the cynomolgus macaque, comprising 1,991,169 non-redundant genes and compared it with the human, mouse and pig gut bacterial gene catalogs. This catalog represents the first gene set generated from a NHP and provides a comprehensive reference resource for metagenomics-based research. The comparison with human, pig and mouse demonstrates that the overlap between different mammalians is very modest at the gene level, but high at the KO functional level. Jonathan *et al* reported that the gut microbiotas of captive NHPs have undergone humanization [18]. Our results also show that the cynomolgus macaque gut microbiome is more similar to the human gut microbiome than the other analyzed mammalian species. However, the degree of similarity is only slightly greater, and the comparisons rather emphasize the quite large

differences at the gene levels between the cynomolgus macaque, human, pig and mouse. However, similarity at the functional level is high between all species. Thus, from a purely metagenomics point of view the use of cynomolgus macaques for biomedical research needs more research. Based on the high genetic similarity between human and cynomolgus macaque it will be of interest to examine if colonization with human microbiotas will be more efficient in cynomolgus macaque than in pig or mouse. We demonstrate that intake of diets with different content of fat and fiber elicited pronounced differences in the gut microbiota of cynomolgus macaques, and that some of these differences recapitulated differences in humans ingesting a low-fat/high fiber diet or a high-fat/low fiber diet [22].

We were able to define a set of core gut bacterial genera based on the available data on the gut microbiomes established by shotgun sequencing of fecal samples from four mammalian species. *Prevotella*, *Bacteroides*, *Clostridium*, *Eubacterium*, *Parabacteroides*, *Ruminococcus*, *Faecalibacterium*, *Roseburia*, *Blautia,* and *Coprococcus* were found to be the dominant bacterial genera present in gut microbiotas of human, cynomolgus macaque, pig, and mouse. However, the relative abundance of these genera varies profoundly between the four species.

A previous case-control comparison of enteric viromes in captive rhesus macaques showed several viruses associated with idiopathic chronic diarrhea [42]. We explored the presence of bacteria phages in the cynomolgus macaque gut microbiome. Interestingly, 15.6% of the genes in the current cynomolgus macaque gut gene catalog could be annotated as bacterial phages. Furthermore, the relative abundance of a subset of these phages differed significantly

between cynomolgus macaques fed the low-fat/high fiber diet and the high-fat/low fiber diet underscoring that phages are abundant in the gut and may change in abundance in response to dietary intake. Thus, phages may play important role in gut homeostasis, but the difference in relative abundance in response to dietary intake may also simply reflect changes in the relative abundance of their bacterial hosts [11].

Methods

Animals, sample collection and transportation.

Fresh feces were sampled from twenty cynomolgus macaques (*Macaca fascicularis*), 13-16 years old. The animals were housed at room temperature with a 12 h light/dark cycle at the JinJieKang Biotechnology Company, Yunnan, China following guidelines approved by the Association for Assessment and Accreditation of Laboratory Animal Care. The experimental protocol was approved by the Animal Care and Use Committee at the JinJieKang Biotechnology Company. The animals had *ad libitum* access to water and the animals were divided into two groups of ten animals. Ten males were fed a low-fat/high fiber diet (8 % of energy from fat, 131 g fiber/ kg) and nine males and one female were fed a high-fat diet/low fiber diet (39 % of energy from fat, 20 g fiber/ kg) for three months. After the three months of feeding the animal were weighted and blood was collected for blood glucose measurements at Kunming Jinyu Medical Laboratory Co., Ltd. Fresh feces was collected, immediately frozen and kept on dry ice during transportation to BGI Shenzhen for further processing.

DNA extractions and sequencing

DNA extraction was performed using 200 mg feces per sample following the method reported by Qin et al [29], except that cell lysis was performed by bead beating the samples twice for 30 s with an incubation of 2 min on ice between beatings. The concentration of fecal DNA was measured using Nanodrop. Following the manufacturer's instructions (Illumina), we constructed one DNA paired-end (PE) library with an insert size of 350 base pairs (bp) for each sample. Metagenomic sequencing was performed on the Illumina 2000 platform by a 100 bp paired-end strategy.

Construction of the gene catalog

Raw reads were filtered with a quality control cutoff (adapter sequence <15bp, 'N' base <3bp, Q>20, final length >30) and host genomic DNA (NCBI accession no. NC_022272.1 - NC_022292.1). An average of 3.49% of the raw reads, which were of low quality or mapped to the host genome DNA, were removed. The remaining reads were considered as high-quality reads. In total, we obtained 131 Gb high-quality data with an average of 6.55 Gb per sample. To construct a cynomolgus macaque gut microbial gene catalog, we assembled the Illumina reads from each sample into longer contigs with the SOAPdenovo2 software (SOAPdenovo2 , RRI:SCR_014986)[23, 29]. A total of 56.43% of the reads were assembled into 2.02 million contigs with a length exceeding 500 bases. Metagene2 [24, 29] was used to

predict open reading frames (ORFs) in contigs obtained for each sample, with an average 220,862 ORFs per sample. A non-redundant gene set comprising ~1.9 M genes was constructed by pairwise comparison of all genes in all samples, using CD-HIT (CD-HIT, RRID:SCR_007105)[25] with identity of >95% and overlap of >90%. Taxonomic assignments (taxonomic database: version March 2012) were made using CARMA3 [30] on the basis of BLASTP against the NCBI-NR database (version September 2013, the same version used for the mouse and pig gut microbiome catalogs).

Functional annotation of gene catalog

We translated the nucleotide sequences of gene catalog into amino acid sequences, then aligned against the proteins or domains in eggNOG v3 (eggNOG, RRID:SCR_002456)[43] and KEGG v59 (KEGG , RRID:SCR_012773)[27] databases using BLASTP (v2.2.24, default parameter except that -F:F). KEGG annotation was performed using an in-house pipeline, where each protein was assigned to a KO when the highest-scoring annotated hit(s) contained at least one alignment over 60 hits.

Quantification of Gene relative abundance

High-quality reads from each sample were aligned against the gene catalog by SOAP2.22 (SOAPaligner/soap2 , RRID:SCR_005503)[26] (with default parameters except for -r 2 -l 30 -M 4 -p 2 -v 10). The relative abundance of each gene in each sample was determined as previously described [5].

Quantification of genus and KO relative abundances

For the relative abundance profile at the genus level, we used the phylogenetic assignment of each gene and summed the relative abundance of genes from the same genus to calculate the abundance of a particular genus. The relative abundance of each genus in a sample constituted the genus profile of that sample. Using the same method, the relative abundance of each KO was calculated from the sum of the relative abundances of the corresponding genes.

KEGG module and pathway enrichment analysis

One-tailed Wilcoxon rank-sum test was performed for all the KOs that occurred in more than five samples and adjusted for multiple testing using the Benjamin-Hochberg procedure. The Z-score for each KO could then be calculated:

$$\boldsymbol{Z_{KO_i}} = \theta^{-1}\,(1-\boldsymbol{P_{KO_i}})$$

where θ^{-1} is the inverse normal cumulative distribution, $\boldsymbol{P_{KO_i}}$ is the adjusted P value for that KO. The aggregated Z-score for a KEGG pathway (or module) is then:

$$Z_{\text{pathway}} = \frac{\mathbf{1}}{\sqrt{\boldsymbol{k}}}\sum \boldsymbol{Z_{KO_i}}$$

where k is the number of KOs involved in the pathway (or module).

We corrected the background distribution of Z_{pathway} by subtracting the mean (μ_k) and dividing by the s.d. (σ_k) of the aggregated Z-scores of 1,000 sets of k KO, chosen randomly from the whole metabolic KO network:

$Z_{adjustedpathway} = \frac{Z_{pathway} - \mu_k}{\sigma_k}$. The $Z_{adjustedpathway}$ was used as the final reporter score for evaluating the enrichment of specific pathways or modules. A reporter score of ≥1.6 (90% confidence according to normal distribution) could be used as a detection threshold for significantly differentiating pathways. This is the same procedure as previously described [44, 45].

Rarefaction curve analysis

Rarefaction analysis was performed to assess the gene richness. For a given number of samples, we performed random sampling 100 times in the cohort with replacement and estimated the total number of genes present in these samples by the Chao1 richness estimator [46].

Enterotypes-like cluster

Genus relative abundances were used for analysis of PAM-based enterotypes-like clusters in cynomolgus macaque, pig, and mouse samples [15, 32]. In this study, the R package “stats” was used to perform a hierarchical clustering of samples using Jensen-Shannon distances followed by PCA using the R package “ade4”.

Comparison with the human, mouse and pig gut gene catalog

The human [31], mouse [15] and pig [32] gut gene sets were compared to the cynomolgus macaque gene set. If two and more genes had > 95% identity and >90% overlap with the query, we considered the genes to be identical. For comparison at the functional level, shared KOs were identified and computed by unique KO ID.

Differences in taxonomic abundance between diets

We analyzed differences in abundance at the phylum, genus and species level using Wilcoxon rank sum test.(P <0.05)

Association between diets and metagenomic markers

To identify associations between metagenome profiles and the two different diets, a two-tailed Wilcoxon rank-sum test [5] implemented in R (R package stats) was used.

Phage genes identification and comparison between the two diet groups

Phage genes were identified from the human, mouse, pig and cynomolgus macaque gut gene catalog using Metafinder [41] (ANI >1.7%). Phage genes that differed in abundance between samples from cynomolgus macaques fed the low-fat/high fiber diet and the high-fat/low fiber diet were selected by Wilcoxon rank sum test (P<0.05).

Availability of supporting data and materials

The metagenomic shotgun sequencing data for all samples have been deposited in the EBI database under the accession code PRJEB22765. Supplemental data is available in the *GigaScience* database, GigaDB [47].

Declarations

List of abbreviations

bp: base pairs; CAZy: carbohydrate-active enzymes; Gb: gigabases; KEGG: Kyoto Encyclopedia of Genes and Genomes; KO: KEGG orthology; NHP: Nonhuman primates; NR: Non-redundant; ORF: open reading frames; PCA: Principal component analysis; PE: paired-end.

Competing interests

The authors declare that they have no competing interests.

Authors' contributions

X.L., and L.X. conceived and directed the project. H.L., and X.L. oversaw the sample

collection and provided phenotypic information. X.L., S.L., Z.X., J.Q., and C.L. performed the bioinformatic analyses and prepared figures and texts for the manuscript. X.L., and S.L. wrote the first draft of the manuscript. L.X., H.J., J.L. L.M. and K.K. made substantial revision of the manuscript. L.X., S.L., L.M., K.K. and J.Q. participated in discussions. All authors contributed to the revision of the manuscript.

Acknowledgments

This research was supported by the National Natural Science Foundation of China (Grant No. 81670606, 81673850), the Shenzhen Municipal Government of China (JSGG20160229172752028, JCYJ20160229172757249). We gratefully acknowledge colleagues at BGI-Shenzhen for DNA extraction, library construction, sequencing, and discussions.

Reference

1. Sender R, Fuchs S and Milo R. Are We Really Vastly Outnumbered? Revisiting the Ratio of Bacterial to Host Cells in Humans. Cell. 2016;164 3:337-40. doi:10.1016/j.cell.2016.01.013.
2. Turnbaugh PJ, Ley RE, Mahowald MA, Magrini V, Mardis ER and Gordon JI. An obesity-associated gut microbiome with increased capacity for energy harvest. Nature. 2006;444 7122:1027-131. doi:10.1038/nature05414.
3. Cani PD, Bibiloni R, Knauf C, Waget A, Neyrinck AM, Delzenne NM, et al. Changes in gut microbiota control metabolic endotoxemia-induced inflammation in high-fat diet-induced obesity and diabetes in mice. Diabetes. 2008;57 6:1470-81. doi:10.2337/db07-1403.
4. Le Chatelier E, Nielsen T, Qin J, Prifti E, Hildebrand F, Falony G, et al. Richness of human gut microbiome correlates with metabolic markers. Nature. 2013;500 7464:541-6. doi:10.1038/nature12506.
5. Qin J, Li Y, Cai Z, Li S, Zhu J, Zhang F, et al. A metagenome-wide association study of gut microbiota in type 2 diabetes. Nature. 2012;490 7418:55-60. doi:10.1038/nature11450.
6. Karlsson FH, Tremaroli V, Nookaew I, Bergstrom G, Behre CJ, Fagerberg B, et al. Gut

metagenome in European women with normal, impaired and diabetic glucose control. Nature. 2013;498 7452:99-103. doi:10.1038/nature12198.

7. Joossens M, Huys G, Cnockaert M, De Preter V, Verbeke K, Rutgeerts P, et al. Dysbiosis of the faecal microbiota in patients with Crohn's disease and their unaffected relatives. Gut. 2011;60 5:631-7. doi:10.1136/gut.2010.223263.

8. Huttenhower C, Kostic Aleksandar D and Xavier Ramnik J. Inflammatory Bowel Disease as a Model for Translating the Microbiome. Immunity. 2014;40 6:843-54. doi:10.1016/j.immuni.2014.05.013.

9. Zhang X, Zhang D, Jia H, Feng Q, Wang D, Liang D, et al. The oral and gut microbiomes are perturbed in rheumatoid arthritis and partly normalized after treatment. Nature medicine. 2015;21 8:895-905. doi:10.1038/nm.3914.

10. Karlsson FH, Fak F, Nookaew I, Tremaroli V, Fagerberg B, Petranovic D, et al. Symptomatic atherosclerosis is associated with an altered gut metagenome. Nature communications. 2012;3:1245. doi:10.1038/ncomms2266.

11. Jie Z, Xia H, Zhong SL, Feng Q, Li S, Liang S, et al. The gut microbiome in atherosclerotic cardiovascular disease. Nature communications. 2017;8 1:845. doi:10.1038/s41467-017-00900-1.

12. Feng Q, Liang S, Jia H, Stadlmayr A, Tang L, Lan Z, et al. Gut microbiome development along the colorectal adenoma-carcinoma sequence. Nature communications. 2015;6:6528. doi:10.1038/ncomms7528.

13. Foster JA and McVey Neufeld KA. Gut-brain axis: how the microbiome influences anxiety and depression. Trends in neurosciences. 2013;36 5:305-12. doi:10.1016/j.tins.2013.01.005.

14. Finegold SM, Dowd SE, Gontcharova V, Liu C, Henley KE, Wolcott RD, et al. Pyrosequencing study of fecal microflora of autistic and control children. Anaerobe. 2010;16 4:444-53. doi:10.1016/j.anaerobe.2010.06.008.

15. Xiao L, Feng Q, Liang S, Sonne SB, Xia Z, Qiu X, et al. A catalog of the mouse gut metagenome. Nature biotechnology. 2015;33 10:1103-8. doi:10.1038/nbt.3353.

16. Lagkouvardos I, Pukall R, Abt B, Foesel BU, Meier-Kolthoff JP, Kumar N, et al. The Mouse Intestinal Bacterial Collection (miBC) provides host-specific insight into cultured diversity and functional potential of the gut microbiota. Nature microbiology. 2016;1 10:16131. doi:10.1038/nmicrobiol.2016.131.

17. Nguyen TL, Vieira-Silva S, Liston A and Raes J. How informative is the mouse for human gut microbiota research? Disease models & mechanisms. 2015;8 1:1-16. doi:10.1242/dmm.017400.

18. Clayton JB, Vangay P, Huang H, Ward T, Hillmann BM, Al-Ghalith GA, et al. Captivity humanizes the primate microbiome. Proc Natl Acad Sci U S A. 2016;113 37:10376-81. doi:10.1073/pnas.1521835113.

19. Angelakis E, Yasir M, Bachar D, Azhar EI, Lagier JC, Bibi F, et al. Gut microbiome and dietary patterns in different Saudi populations and monkeys. Scientific reports. 2016;6:32191. doi:10.1038/srep32191.

20. He X, Slupsky CM, Dekker JW, Haggarty NW and Lönnerdal B. Integrated Role of Bifidobacterium animalis subsp. lactis Supplementation in Gut Microbiota, Immunity, and Metabolism of Infant Rhesus Monkeys. mSystems. 2016;1 doi:10.1128/mSystems.00128-16.

21. Hale VL, Tan CL, Niu K, Yang Y, Knight R, Zhang Q, et al. Diet Versus Phylogeny: a Comparison of Gut Microbiota in Captive Colobine Monkey Species. Microbial ecology. 2017; doi:10.1007/s00248-017-1041-8.
22. Wu GD, Chen J, Hoffmann C, Bittinger K, Chen YY, Keilbaugh SA, et al. Linking long-term dietary patterns with gut microbial enterotypes. Science. 2011;334 6052:105-8. doi:10.1126/science.1208344.
23. Luo R, Liu B, Xie Y, Li Z, Huang W, Yuan J, He G, Chen Y, Pan Q, Liu Y, Tang J, Wu G, Zhang H, Shi Y, Liu Y, Yu C, Wang B, Lu Y, Han C, Cheung DW, Yiu SM, Peng S, Xiaoqian Z, Liu G, Liao X, Li Y, Yang H, Wang J, Lam TW, Wang J. SOAPdenovo2: an empirically improved memory-efficient short-read de novo assembler. Gigascience. 2012 Dec 27;1(1):18. doi: 10.1186/2047-217X-1-18.
24. Noguchi H, Park J and Takagi T. MetaGene: prokaryotic gene finding from environmental genome shotgun sequences. Nucleic Acids Res. 2006;34 19:5623-30. doi:10.1093/nar/gkl723.
25. Li W and Godzik A. Cd-hit: a fast program for clustering and comparing large sets of protein or nucleotide sequences. Bioinformatics. 2006;22 13:1658-9. doi:10.1093/bioinformatics/btl158.
26. Li R, Yu C, Li Y, Lam TW, Yiu SM, Kristiansen K, et al. SOAP2: an improved ultrafast tool for short read alignment. Bioinformatics. 2009;25 15:1966-7. doi:10.1093/bioinformatics/btp336.
27. Kanehisa M, Sato Y, Kawashima M, Furumichi M and Tanabe M. KEGG as a reference resource for gene and protein annotation. Nucleic Acids Res. 2016;44 D1:D457-62. doi:10.1093/nar/gkv1070.
28. Cantarel BL, Coutinho PM, Rancurel C, Bernard T, Lombard V and Henrissat B. The Carbohydrate-Active EnZymes database (CAZy): an expert resource for Glycogenomics. Nucleic Acids Res. 2009;37 Database issue:D233-8. doi:10.1093/nar/gkn663.
29. Qin J, Li R, Raes J, Arumugam M, Burgdorf KS, Manichanh C, et al. A human gut microbial gene catalogue established by metagenomic sequencing. Nature. 2010;464 7285:59-65. doi:10.1038/nature08821.
30. Gerlach W and Stoye J. Taxonomic classification of metagenomic shotgun sequences with CARMA3. Nucleic Acids Res. 2011;39 14:e91. doi:10.1093/nar/gkr225.
31. Li J, Jia H, Cai X, Zhong H, Feng Q, Sunagawa S, et al. An integrated catalog of reference genes in the human gut microbiome. Nature biotechnology. 2014;32 8:834-41. doi:10.1038/nbt.2942.
32. Xiao L, Estelle J, Kiilerich P, Ramayo-Caldas Y, Xia Z, Feng Q, et al. A reference gene catalogue of the pig gut microbiome. Nat Microbiol. 2016:16161. doi:10.1038/nmicrobiol.2016.161.
33. Koren O, Knights D, Gonzalez A, Waldron L, Segata N, Knight R, et al. A guide to enterotypes across the human body: meta-analysis of microbial community structures in human microbiome datasets. PLoS computational biology. 2013;9 1:e1002863. doi:10.1371/journal.pcbi.1002863.
34. Arumugam M, Raes J, Pelletier E, Le Paslier D, Yamada T, Mende DR, et al. Enterotypes of the human gut microbiome. Nature. 2011;473 7346:174-80. doi:10.1038/nature09944.
35. Zhu L, Baker SS, Gill C, Liu W, Alkhouri R, Baker RD, et al. Characterization of gut microbiomes in nonalcoholic steatohepatitis (NASH) patients: a connection between endogenous alcohol and NASH. Hepatology. 2013;57 2:601-9. doi:10.1002/hep.26093.

36. Ding T and Schloss PD. Dynamics and associations of microbial community types across the human body. Nature. 2014;509 7500:357-60. doi:10.1038/nature13178.

37. Madsen L, Myrmel LS, Fjære E, Liaset B and Kristiansen K. Links between Dietary Protein Sources, the Gut Microbiota, and Obesity. Front Physiol. 2017;8 1047 doi:10.3389/fphys.2017.01047.

38. Wos-Oxley M, Bleich A, Oxley AP, Kahl S, Janus LM, Smoczek A, et al. Comparative evaluation of establishing a human gut microbial community within rodent models. Gut microbes. 2012;3 3:234-49. doi:10.4161/gmic.19934.

39. Turnbaugh PJ, Ridaura VK, Faith JJ, Rey FE, Knight R and Gordon JI. The effect of diet on the human gut microbiome: a metagenomic analysis in humanized gnotobiotic mice. Science translational medicine. 2009;1 6:6ra14. doi:10.1126/scitranslmed.3000322.

40. Zhang L, Bahl MI, Roager HM, Fonvig CE, Hellgren LI, Frandsen HL, et al. Environmental spread of microbes impacts the development of metabolic phenotypes in mice transplanted with microbial communities from humans. The ISME journal. 2017;11 3:676-90. doi:10.1038/ismej.2016.151.

41. Jurtz VI, Villarroel J, Lund O, Voldby Larsen M and Nielsen M. MetaPhinder-Identifying Bacteriophage Sequences in Metagenomic Data Sets. PloS one. 2016;11 9:e0163111. doi:10.1371/journal.pone.0163111.

42. Kapusinszky B, Ardeshir A, Mulvaney U, Deng X and Delwart E. Case-Control Comparison of Enteric Viromes in Captive Rhesus Macaques with Acute or Idiopathic Chronic Diarrhea. J Virol. 2017;91 18 doi:10.1128/JVI.00952-17.

43. Powell S, Szklarczyk D, Trachana K, Roth A, Kuhn M, Muller J, et al. eggNOG v3.0: orthologous groups covering 1133 organisms at 41 different taxonomic ranges. Nucleic acids research. 2012;40 Database issue:D284-9. doi:10.1093/nar/gkr1060.

44. Patil KR and Nielsen J. Uncovering transcriptional regulation of metabolism by using metabolic network topology. Proceedings of the National Academy of Sciences of the United States of America. 2005;102 8:2685-9. doi:10.1073/pnas.0406811102.

45. Oliveira AP, Patil KR and Nielsen J. Architecture of transcriptional regulatory circuits is knitted over the topology of bio-molecular interaction networks. BMC systems biology. 2008;2:17. doi:10.1186/1752-0509-2-17.

46. Chao A. Estimating the population size for capture-recapture data with unequal catchability. Biometrics. 1987;43 4:783-91.

47. Li X; Liang S; Xia Z; Qu J; Liu H; Liu C; Yang H; Wang J; Madsen L; Hou Y; Li J; Jia H; Kristiansen K; Xiao L (2018): Supporting data for "Establishment of a Macaca fascicularis gut microbiome gene catalog and comparison with the human, pig and mouse gut microbiomes" GigaScience Database. http://dx.doi.org/10.5524/100470

Figure legend

Figure 1. Rarefaction curve based on gene numbers and taxonomic annotation of the cynomolgus macaque gut bacterial gene catalog.

a. Rarefaction curve based on the gene numbers of all cynomolgus macaque samples and the individual subgroups.

b. Taxonomic annotation of 1.9M cynomolgus macaque gut bacterial gene catalog. More than 65% of the genes from cynomolgus macaque gut bacterial gene catalog could be annotated to the bacterial superkingdom. 13.91% of the genes could be annotated to the genus level.

Figure 2. Characteristic of the cynomolgus macaque gut microbiota.

a. The top 10 phyla in the cynomolgus macaque gut microbiota. *Bacteroidetes* and *Firmicutes* are the main two phyla in the cynomolgus macaque gut microbiota.

b. The top 20 genera in the cynomolgus macaque gut microbiota. *Prevotella* is the main genus in the cynomolgus macaque gut microbiota.

Figure 3. Comparison with the human, mouse and pig gut microbiomes.

a. Unique non-redundant genes in the cynomolgus macaque, human, pig and mouse gut bacterial gene catalogs. Less than 0.4% genes overlapped between all the four species, which emphasizes the marked differences between the cynomolgus macaque, human, pig and mouse gut microbiome at the gene level.

b. Comparison of the cynomolgus macaque, human, pig and mouse microbiotas based on KEGG annotation, which emphasizes the functional similarity between the cynomolgus macaque, human, pig and mouse gut microbiota despite the marked differences at the gene

level shown in a.

c. PCA based on overlapping KOs of the cynomolgus macaque, human, mouse, and pig gut microbiota.

d. The top 20 core genera in the cynomolgus macaque, human, pig and mouse gut microbiota. The 10 shared genera are marked in red.

Figure 4. Diet-related differences in the cynomolgus macaque gut microbiota.

a. PCA of cynomolgus macaque samples based on gene profiles.

b. KEGG functional classification of the 82,120 gene makers. The black bars represent the total percentage in the 1.9M gene catalog. The gray bars represent gene markers enriched in high-fat/low fiber diet group. The white bars represent gene marker rate enriched in the low-fat/high fiber control group.

Additional files

Additional file 1: Data production from cynomolgus macaque fecal samples.

Additional file 2: KEGG pathway classification and CAZy classification.

a. KEGG pathway classification. 53.09% of the cynomolgus macaque gene catalog could be annotated to the KO level.

b. CAZy classification. 3.41% of the cynomolgus macaque gene catalog could be annotated in the CAZy database.

Additional file 3: The average abundance of the 80 core genera shared among all cynomolgus macaque individuals.

Additional file 4: The enterotype-like cluster in the cynomolgus macaque, mouse, and pig samples.

a. Enterotype-like clusters in the cynomolgus macaque samples.

b. Abundances of the main contributors to each enterotype-like cluster in the cynomolgus macaque samples.

c. Enterotype-like clusters in the mouse samples.

d. Abundances of the main contributors to each enterotype-like cluster in mouse samples.

e. Enterotype-like clusters in the pig samples.

f. Abundances of the main contributors to each enterotype-like cluster in the pig samples.

Additional file 5: The general features of the human, macaque, mouse, and pig gut bacterial gene catalogs.

Additional file 6: Mapping ratio of cynomolgus macaque, human, pig and mouse samples.

a. Average mapping ratio of cynomolgus macaque sample reads to 1 million genes randomly selected (10 times) from the cynomolgus macaque, human, pig and mouse gene catalogs.

b. Average mapping ratio of 20 samples from mouse, pig, cynomolgus macaque, and human

mapped to 9.9M human gut gene catalogs.

Additional file 7: Alpha diversity

a. Alpha diversity calculated as Shannon effective of the cynomolgus macaque gut microbiota compared to the human, pig, and mouse gut microbiota. The alpha diversity of pig gut microbiota is highest compared to the gut microbiota of the other three species, and the alpha diversity of the human gut microbiota is lowest.

b. Alpha diversity calculated as Shannon effective of the cynomolgus macaque gut microbiota in samples from animals fed the low-fat/high fiber diet or the high-fat/low fiber diet, with the latter tending to exhibit lower alpha diversity.

Additional file 8: Core genera in the gut microbiota of the cynomolgus macaque, pig, human, and mouse.

a. Venn diagram of core genera in the cynomolgus macaque, pig, human, and mouse;

b: heatmap of the 32 mammalian core genera.

Additional file 9: Genera networks of 32 mammalian core genera in each mammalian gut microbiota.

a. Genera network of 32 mammalian core genera in the human gut microbiota.
b. Genera network of 32 mammalian core genera in the cynomolgus macaque gut microbiota.
c. Genera network of 32 mammalian core genera in the mouse gut microbiota.

d. Genera network of 32 mammalian core genera in the pig gut microbiota.

The size of the node is proportional to the genus abundance. Node color corresponds to phylum taxonomic classification. Edge color represents positive (red) and negative (green) correlations, and the edge thickness is equivalent to the absolute values of Spearman correlation coefficient. (q-value < 0.05)

Additional file 10: Correlative relationships of 32 mammalian core genera showed in additional file 9.

Additional file 11: Phenotypic information of all cynomolgus macaque individuals.

Additional file 12: Analysis of differences in abundance at the phylum, genus and species level.

Additional file 13: Enrichment of KEGG modules in the gut microbiotas of animal fed the low-fat/high fiber diet and the high-fat low fiber diet.

Additional file 14: Enrichment of KEGG pathways in cynomolgus macaques fed the high-fat/low fiber or the low-fat/high fiber diets.

Additional file 15: Summary of the phage genes identified in the cynomolgus macaque, human, pig, and mouse gut microbiome gene catalogs.

Additional file 16: List of predicted phage genes that differ significantly in abundance between the high-fat/low fiber diet and low-fat/high fiber diet fed cynomolgus macaque groups.

Additional file 17: Heatmap of the abundance of predicted phage genes that differ significantly in abundance between the high-fat/low fiber diet and low-fat/high fiber diet fed cynomolgus macaque groups.

We selected phage genes with zero abundance in all the low-fat/high fiber diet fed cynomolgus macaque individuals and exhibited non-zero abundance in all the high-fat/low fiber diet fed cynomolgus macaque individuals and vice versa, i.e. zero abundance in all the high-fat/low fiber diet fed cynomolgus macaque individuals and non-zero abundance in all the low-fat/high fiber diet fed cynomolgus macaque individuals

Additional file 18: Selected scripts used for bioinformatics analyses.

Click here to access/download;Figure;Figure 1.pdf.pdf

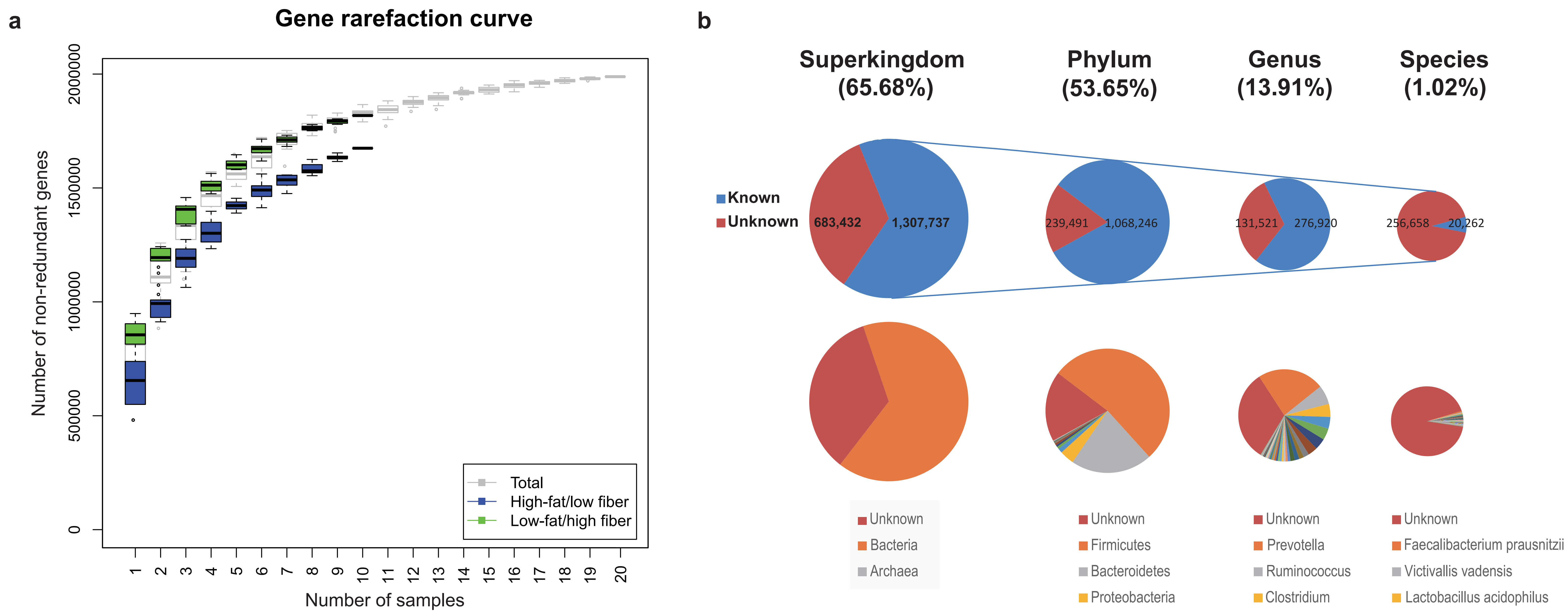

Click here to access/download;Figure;Figure 2.pdf.pdf

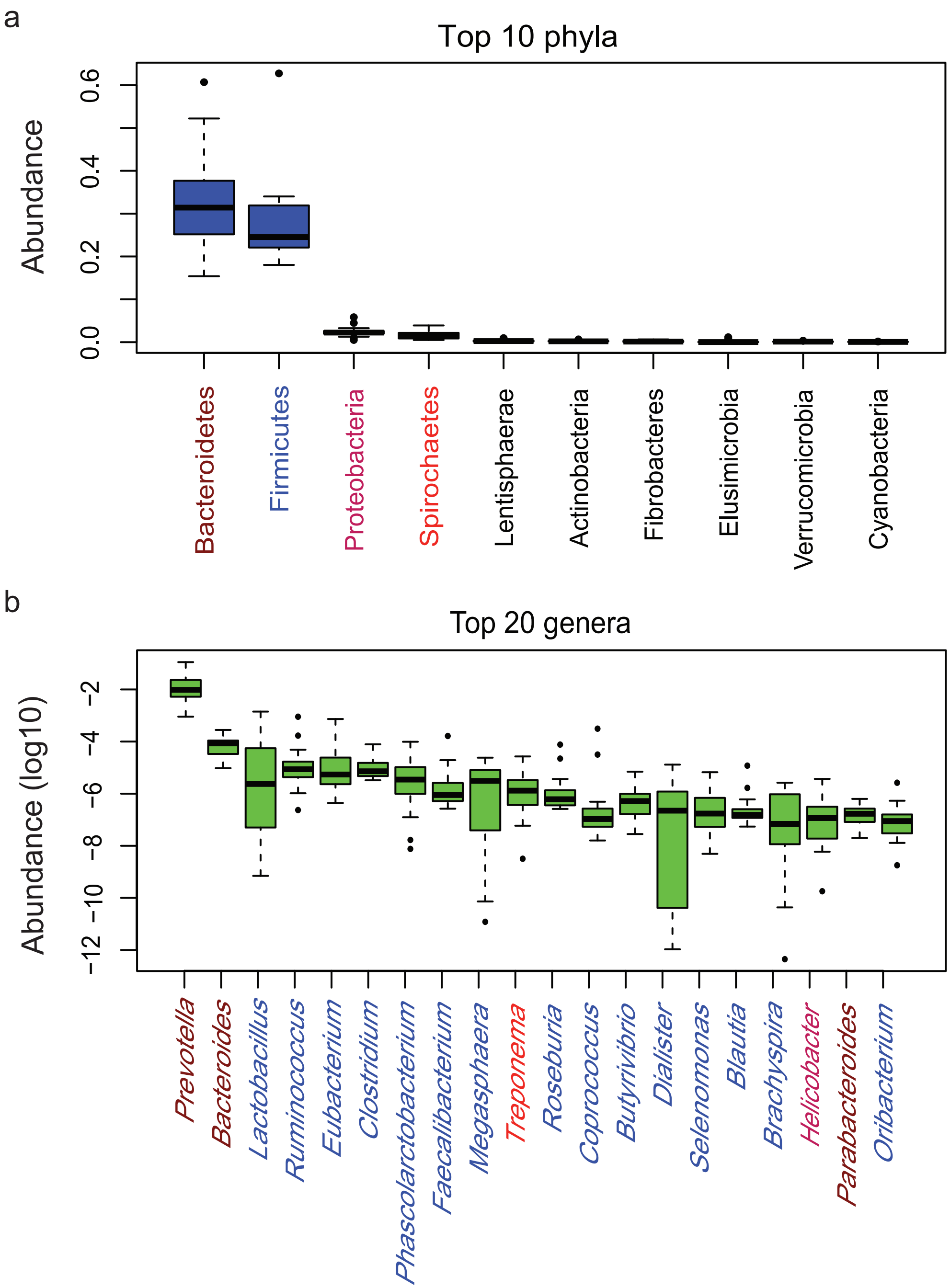

Click here to access/download;Figure;Figure 3pdf.pdf

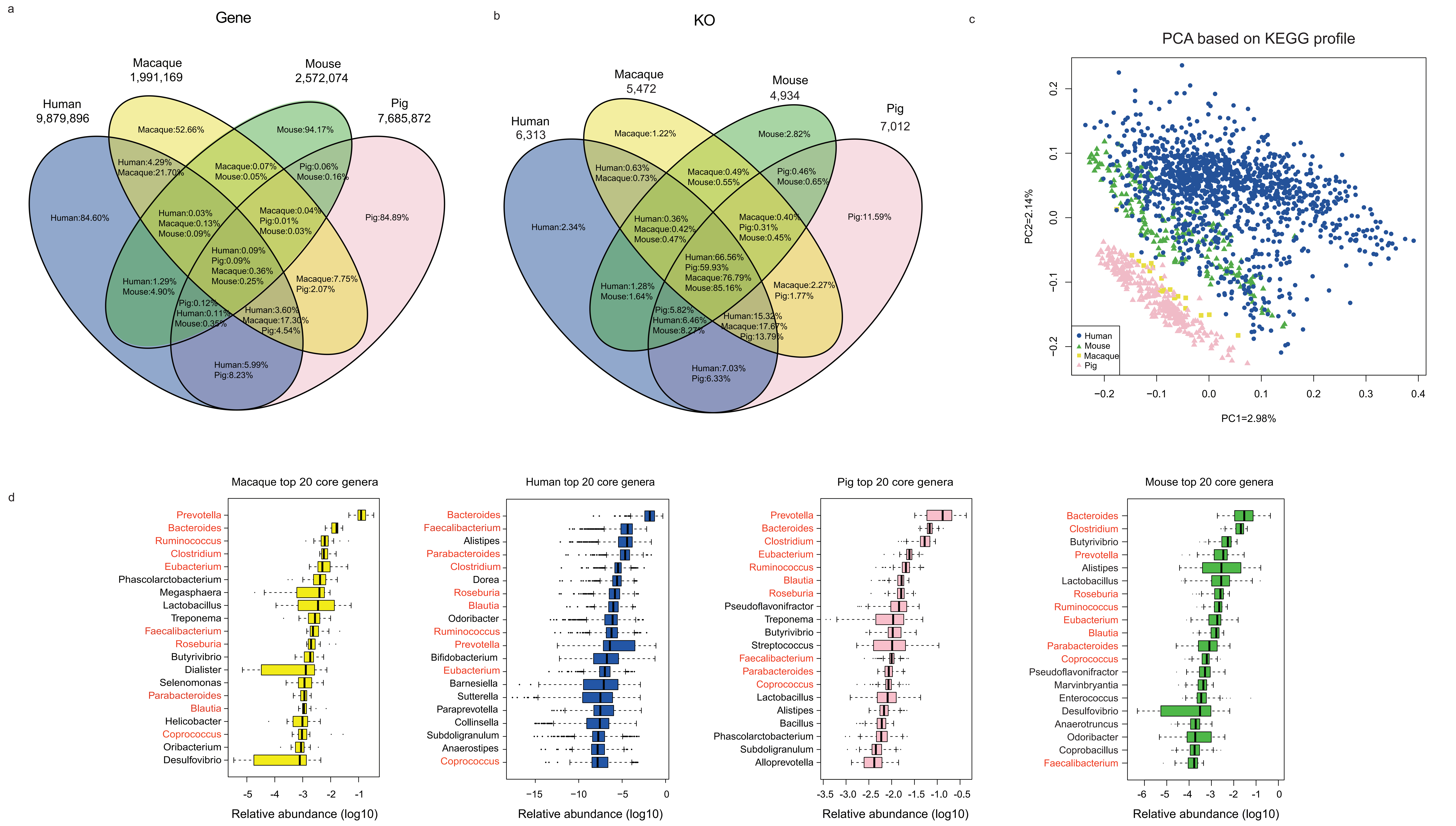

Click here to access/download;Figure;Figure 4pdf.pdf

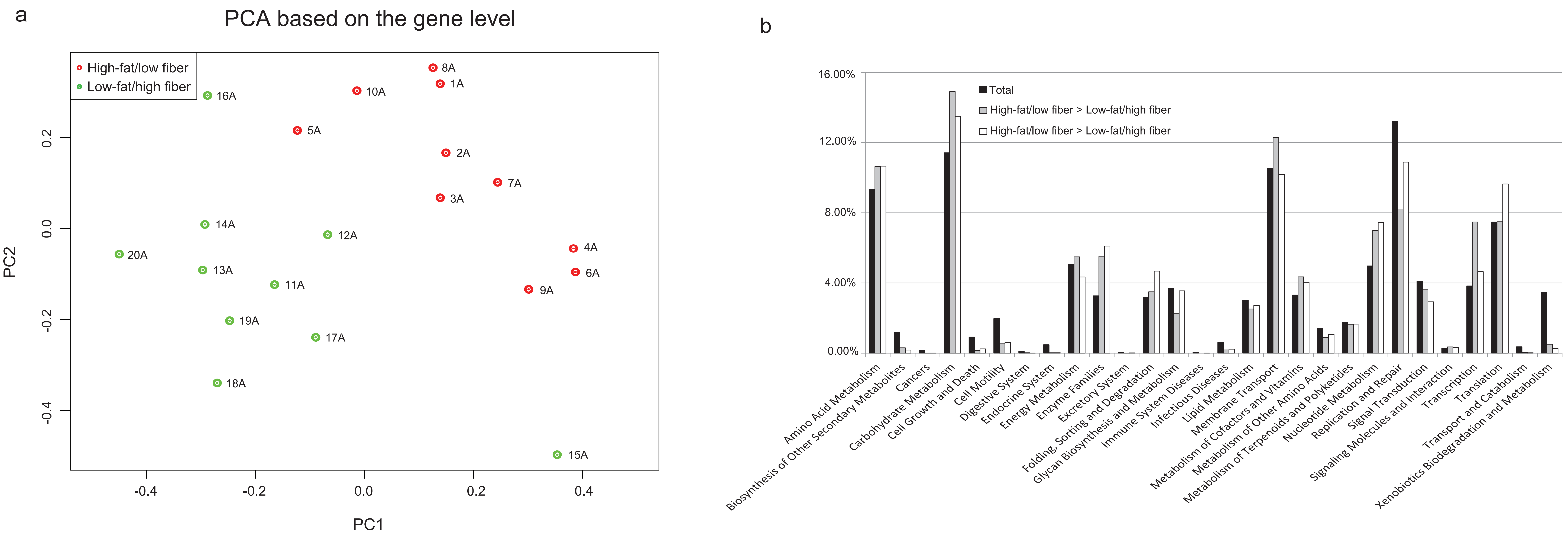

Click here to access/download
Supplementary Material
Additional file 1.xlsx

Click here to access/download
Supplementary Material
Additional file 2.pdf

Click here to access/download
Supplementary Material
Additional file 3.xlsx

Click here to access/download
Supplementary Material
Additional file 4.pdf

Click here to access/download
Supplementary Material
Additional file 5.xlsx

Click here to access/download
Supplementary Material
Additional file 6.pdf

Click here to access/download
Supplementary Material
Additional file 7.pdf

Click here to access/download
Supplementary Material
Additional file 8.pdf

Click here to access/download
Supplementary Material
Additional file 9.pdf

Click here to access/download
Supplementary Material
Additional file 10.xlsx

Click here to access/download
Supplementary Material
Additional file 11.xlsx

Click here to access/download
Supplementary Material
Additional file 12.xlsx

Click here to access/download
Supplementary Material
Additional file 13.xlsx

Click here to access/download
Supplementary Material
Additional file 14.xlsx

Click here to access/download
Supplementary Material
Additional file 15.xlsx

Click here to access/download
Supplementary Material
Additional file 16.xlsx

Click here to access/download
Supplementary Material
Additional file 17.pdf

Click here to access/download
Supplementary Material
Additional file 18.pdf

Click here to access/download
Supplementary Material
Revised Manuscript_tracked change.docx

Click here to access/download
Supplementary Material
Response_to_Reviewer_Comments_Original_Submission.pdf

Click here to access/download
Supplementary Material
Response_to_Reviewer_Comments_Revision_1.pdf
