# Supplementary material for: Establishment of a Macaca fascicularis gut microbiome gene catalog and comparison with the human, pig, and mouse gut microbiomes
Source: Gigascience. 2018 Aug 18;7(9):giy100. doi: 10.1093/gigascience/giy100 (PMC6137240; doi:10.1093/gigascience/giy100)
Supplement: Additional Files [file giy100_supplemental_files.zip › Additional file 18.pdf]

Script for enterotypes-like cluster (additional file 4)

```
argv <- commandArgs(T)
library(fpc)
library(ade4)
####JSD distance function####
kld <- function(x,y){
	x <- x+0.000000001
	y <- y+0.000000001
	kld <- sum(x*log(x/y))
	kld
}
jsd <- function(x){
	ds <- dim(x)
	jsd <- matrix(0, ds[2], ds[2])
	for (i in 1:ds[2]) {
		for (j in 1:ds[2]) {
			m <- (x[,i]+x[,j])/2
			jsd[i,j] <- kld(x[,i],m)/2+kld(x[,j],m)/2
			diag(jsd) <- 0
		}
	}
		d <- sqrt(jsd)
		d
}
dat=read.table(argv[1],head=T,sep="\t",row.names=1,check.name=F)
dat=as.matrix(dat)
dat=dat[rowSums(dat)!=0,]
id=read.table(argv[2],head=F,check.name=F)
cn <- colnames(dat)
head(cn)
gid <- pmatch(cn , id[,1])
which(is.na(gid))
dat <- dat[,!is.na(gid)]
id <- id[gid[!is.na(gid)],]
dat.scale=sweep(dat,2,apply(dat,2,sum),"/")
######
hc=hclust(as.dist(jsd(dat.scale)),method="ward.D")
cu=cutree(hc,k=3)
names(cu)=colnames(dat)
names(cu)=colnames(dat)
cu=as.matrix(cu)
colnames(cu)="Enterotype"
write.table(cu,"JSD.hclust.3.Enterotype.txt",sep="\t", quote=F)
######s.calss
pdf("pca.pdf")
factor=factor(cu[,1])
dat.dudi=dudi.pca(t(sqrt(dat.scale)),scale=F,scannf=F,nf=2,center=T)
li=dat.dudi$li[,1:2]
xli=range(li[,1])
yli=range(li[,2])
```

```
maxx=max(xli,yli)
minx=min(xli,yli)
pch=as.numeric(id[,2])+15
s.class(dat.dudi$li,factor,col=2:4,grid=F,pch=pch,cpoint=1)
legend(maxx*0.4,maxx*0.5,legend=levels(id[,2]),pch=unique(pch),col=1)
bb=dat.dudi$c1[order(sqrt(dat.dudi$c1[,1]^2+dat.dudi$c1[,2]^2),decreasing=T),][1:3,]
cutoff <- 0.3
  for(i in 1:dim(bb)[1]){
        text(bb[i,1]*cutoff,bb[i,2]*cutoff,labels=rownames(bb)[i],font=2,cex=1.1)
        }
dev.off()
pdf("High.abundance.genus.distribution.pdf",10,3)
dat.max=dat[pmatch(rownames(bb),rownames(dat)),]
par(mfcol=c(1,3))
for(i in 1:dim(dat.max)[1]){
        boxplot(dat.max[i,]~factor,col=2:4,ylab="abundance",xlab="Enterotype",main=rownames(dat.max)[i])
            }
dev.off()
```

Script for correlative relationships of 32 mammalian core genera in human, macaque, mouse and pig gut gene catalogs (additional file 10)

```
argv <- commandArgs(T)
if(length(argv) != 2 ){stop("Rscript    correlate.v2.r      [ profile ] [ cor.txt]\n")}
dat <- read.table(argv[1],head = T,check.names=F,sep="\t",row.names=1)
dat <- as.matrix(dat)
out=matrix(NA,nrow=nrow(dat)*nrow(dat)/2,ncol=6)
colnames(out)=c("ID","ID","corr","corr.pvalue","corr.qvalue","+/-")
name <- paste(argv[2], "corr.txt",sep = ".")
num=0
for (i in 1:(nrow(dat)-1)) {
      for (j in (i+1):nrow(dat)){
             num=num+1
             out[num,1]=rownames(dat)[j]
             out[num,2]=rownames(dat)[i]
             out[num,3]=cor(as.numeric(dat[j,]),as.numeric(dat[i,]),method= "spearman")
             out[num,4]=cor.test(as.numeric(dat[j,]),as.numeric(dat[i,]),method= "spearman")$p.v
             if(out[num,3] > 0){
                  out[num,6] = "+"
                  }
             else{
                  out[num,6]= "-"
                  }
              }
}
              x=out[,4]
              x.a <- p.adjust(x,method = "BH")
              y <- cbind(x,x.a)
              r <- order(x)
              y <- y[r,]
```

```
		fdr <- y[max(which(y[,1] < 0.05)),]
		names(fdr) <- NULL
		cat("False discovery rate:",fdr[2],"\n")
		out[,5]=x.a
out=out[out[,5]<0.05,]
write.table(out,name,row.names=F,col.names=T,sep="\t",quote=F)
```

Script for PCA based on KEGG profile (Figure 2c)

```
argv <- commandArgs(T)
if(length(argv) < 4){
		stop("Usage Rscript svd_pca.r [input profile file]	[uniformization T or F] [sqrt T or F] [prefix] [loading columns numbs to output (Default 2)]")
}
data=read.table(file=argv[1],sep="\t",check.names=F,head=T,row.names=1)
dat=data[,c(1:length(colnames(data)))]
rownames(dat)=rownames(data)
dat <- as.matrix(dat)
rs <- rowSums(dat)
dat <- dat[rs!=0,]
cn <- colnames(dat)
rn <- rownames(dat)
rm(rs)
gc()	####
uniformization <- argv[2]
sq <- argv[3]
if(uniformization == "T") {
		dat <- sweep(dat,2,apply(dat,2,sum),"/")	###	uniformization
		print("uniformization is done")
}
if(sq == "T") {
		dat <- sqrt(dat)	### sqrt
		print("sqrt is done")
}
dat <- t(dat)
dat <- scale(dat, center = T , scale = F)
s <- svd(dat)
attach(s)
eig <- (d^2)/dim(dat)[1]		### eigen values
scs <- u %*% diag(d)			### scores
pfx <- argv[4]
write.table(d,paste(pfx,"singular_values.txt",sep="_"),quote=F,sep="\t",col.names=F,row.names = T)		### Singular Values
write.table(eig,paste(pfx,"eigen_values.txt",sep="_"),quote=F,sep="\t",col.names=F,row.names = T)
write.table(scs,paste(pfx,"scores.txt",sep="_"),quote=F,sep="\t",col.names=paste("PC",1:dim(scs)[2],sep = ""), row.names = cn)
cores (Pc)
nc <- argv[5]
if(is.na(nc)){
	write.table(v[,1:2],paste(pfx,"eigen_vectors.txt",sep="_"),quote=F,sep="\t",col.names=paste("PC",1:2,sep=""),
```

```
        row.names = rn)
}else{
        nc <-as.numeric(nc)
        write.table(v[,1:nc],paste(pfx,"eigen_vectors.txt",sep="_"),quote=F,sep="\t",col.names=paste("PC",1:nc,sep=""),
        row.names = rn)
}
detach(s)
```

Quantification of genus and KO relative abundances

After getting the taxonomy annotation of each gene and the gene relative abundance profile, we calculated the genus relative abundance by summing the relative abundances of genes that belong to the same genus. The KO relative abundances are also calculated in the same way.

Related script:

```
#! /usr/bin/perl
use strict;
use warnings;
use FindBin qw($Bin $Script);
use File::Basename qw(basename dirname);
use Getopt::Long;
use Data::Dumper;
use Pod::Text;
use threads;
use threads::shared;

##initialize some parameters fot GetOptions
our ($parallelism, $gene_profile, $outdir, $anno_profile);
our (%parallelism_anno_hash, @line_ary, @sample_ary);
our (%anno_profile_hash, @anno_profile_ary, $i, $each);
GetOptions(
                "g=s"=>\$parallelism,
                "f=s"=>\$gene_profile,
                "o=s"=>\$outdir,
);
##get the introduction information
die `pod2text $0` if ( !$parallelism || !$gene_profile);
$outdir ||= ".";
$outdir =~ s/\/$//;
my $pwd = $ENV{'PWD'};
$outdir = "$pwd/$outdir" if($outdir !~ /^\//);
$parallelism = "$pwd/$parallelism" if($parallelism !~ /^\//);
$gene_profile = "$pwd/$gene_profile" if($gene_profile !~ /^\//);
unless(-e $outdir){
                `mkdir -p $outdir`;
}
#profile result file
$anno_profile = $outdir.'/anno.profile';
open GS, $parallelism or die "can't read $parallelism:$!\n";
while(<GS>){
                chomp;
                @line_ary = split /\t/;
```

```
		$parallelism_anno_hash{$line_ary[0]} = $line_ary[1];
}
close GS;
open GP, $gene_profile or die "can't read $gene_profile:$!\n";
$_=<GP>;
chomp;
@sample_ary = split(/\t/, $_);
while(<GP>){
		chomp;
		@line_ary = split /\t/;
		for($i = 1; $i<=$#line_ary; ++$i){
				if($parallelism_anno_hash{$line_ary[0]}){
						$anno_profile_hash{$parallelism_anno_hash{$line_ary[0]}}{$sample_ary[$i]} += $line_ary[$i];
				}else{
						#$anno_profile_hash{"other"}[$sample_ary[$i]] += $line_ary[$i];
				}
		}
}
close GP;
open annoP, ">$anno_profile" or die "can't write $anno_profile:$!\n";
for($i=0; $i<=$#sample_ary; ++$i){
		print annoP $sample_ary[$i];
		if($i != $#sample_ary){
				print annoP "\t";
		}
}
print annoP "\n";
shift @sample_ary;
foreach my $anno (sort keys %anno_profile_hash){
		my $anno_nospace = $anno;
		$anno_nospace =~ s/\s+/\_/g;
		print annoP $anno_nospace."\t";
		for( $i=0; $i<=$#sample_ary; ++$i){
				print annoP $anno_profile_hash{"$anno"}{$sample_ary[$i]};
				if($i != $#sample_ary){
						print annoP "\t";
				}
		}
		print annoP "\n";
}
close annoP;
```
