# Supplementary figures and images for: Establishment of a Macaca fascicularis gut microbiome gene catalog and comparison with the human, pig, and mouse gut microbiomes
Source: Gigascience. 2018 Aug 18;7(9):giy100. doi: 10.1093/gigascience/giy100 (PMC6137240; doi:10.1093/gigascience/giy100)

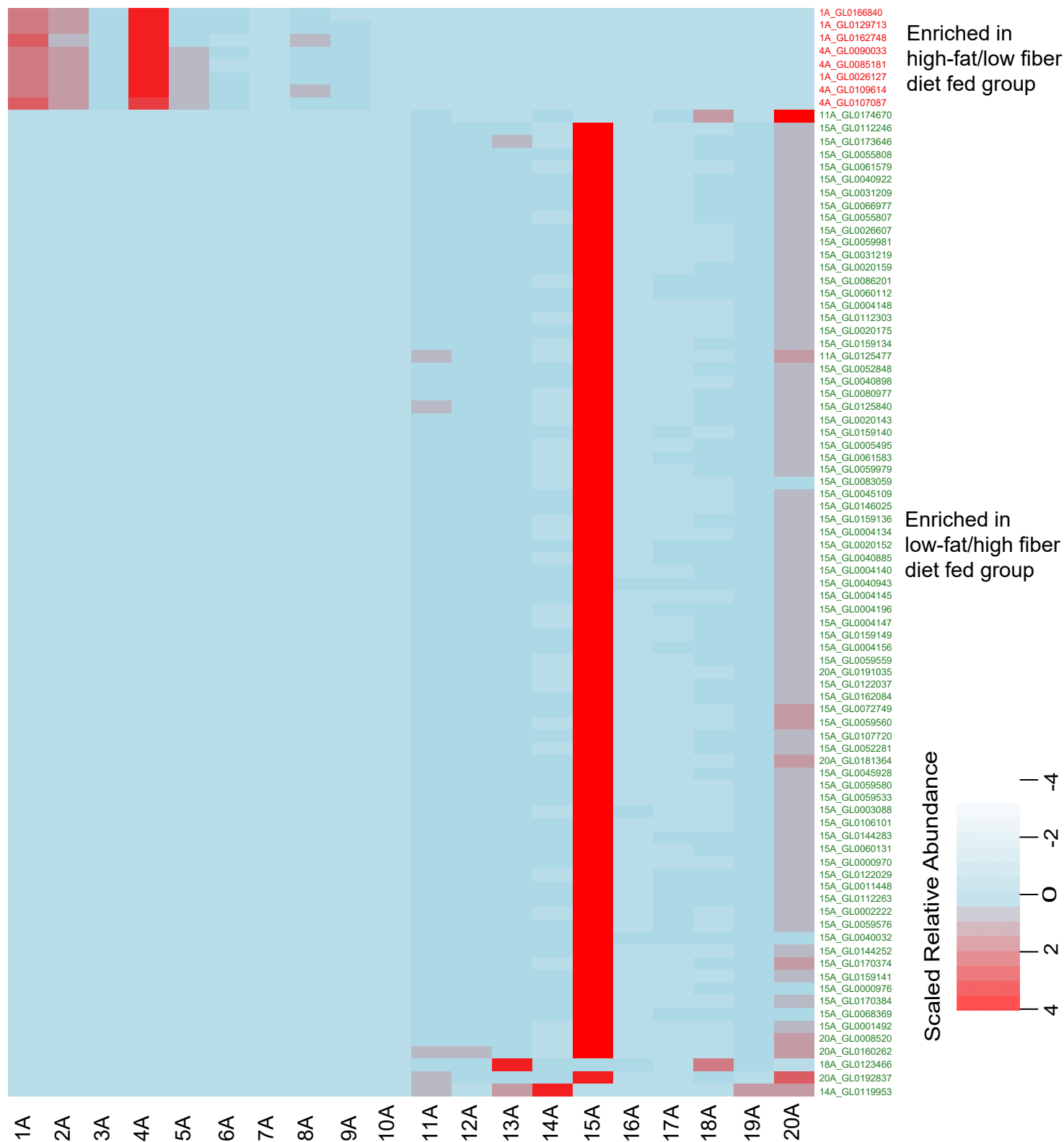

Supplement: Additional Files [file giy100_supplemental_files.zip › Additional file 17.pdf]

a

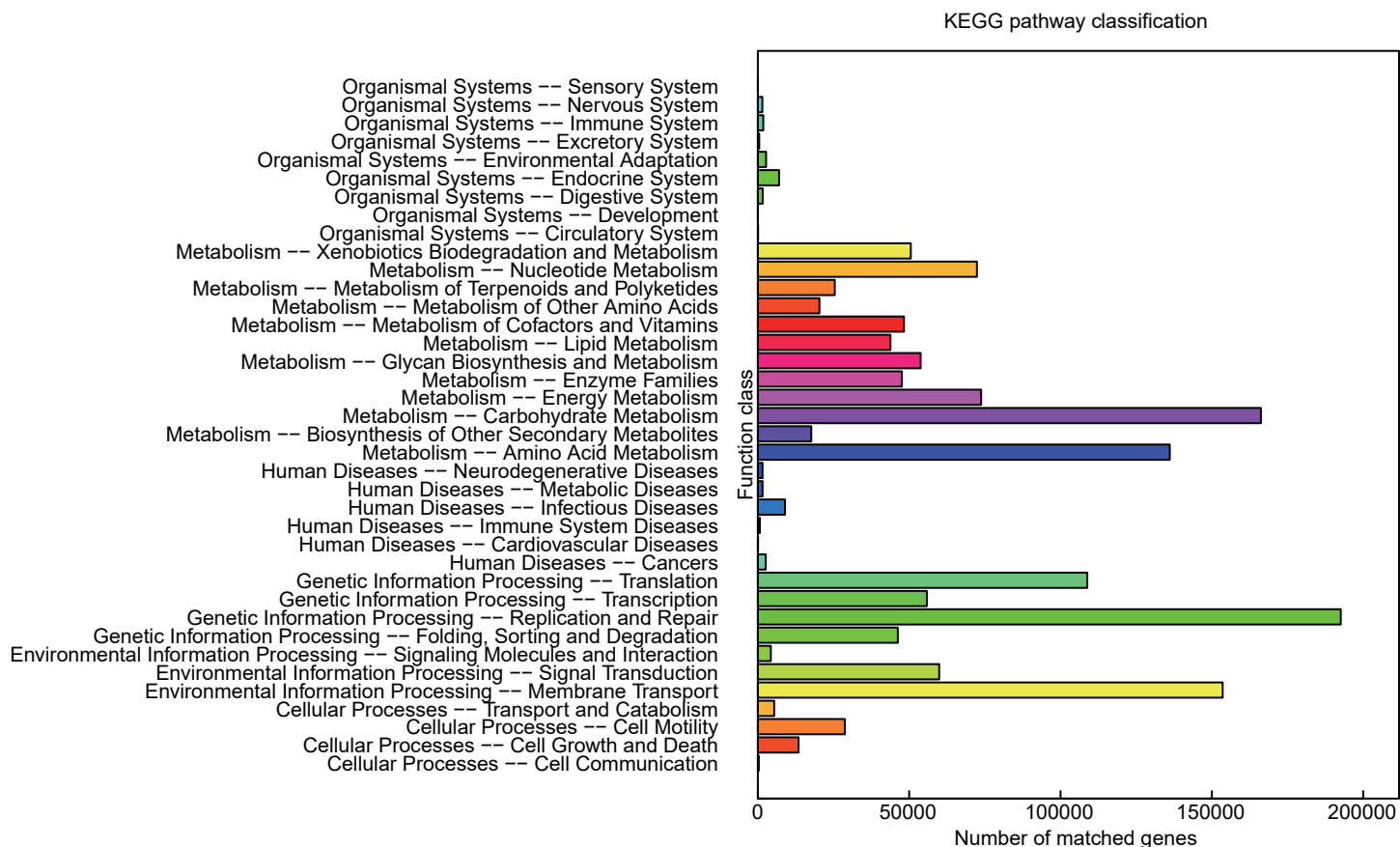

b

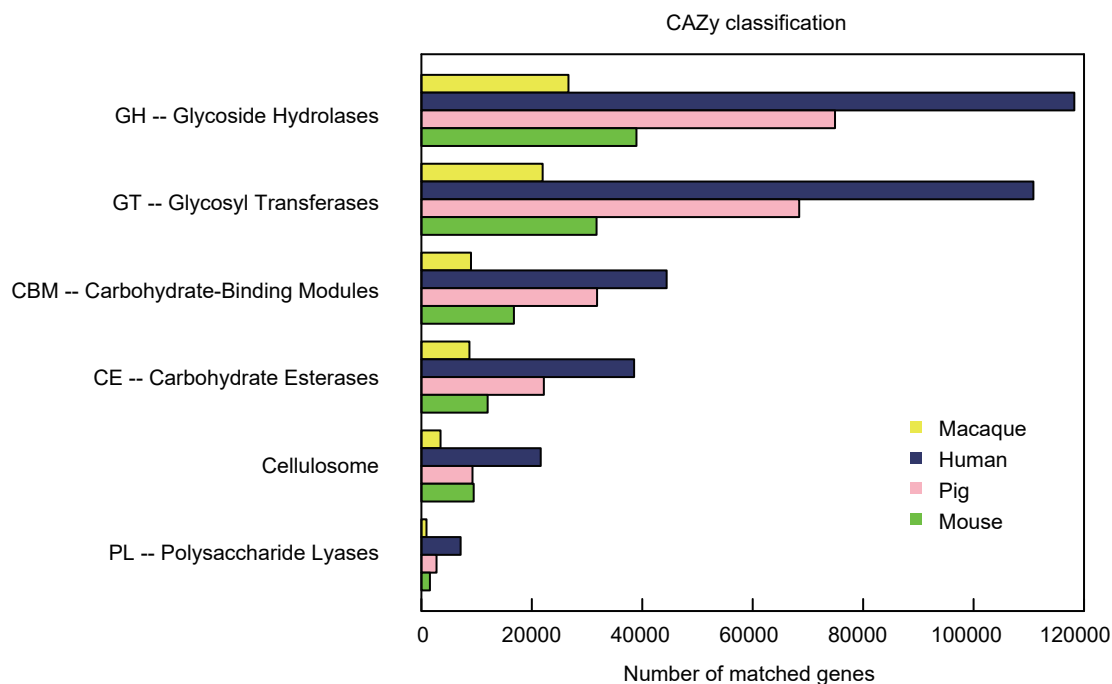

Supplement: Additional Files [file giy100_supplemental_files.zip › Additional file 2.pdf]

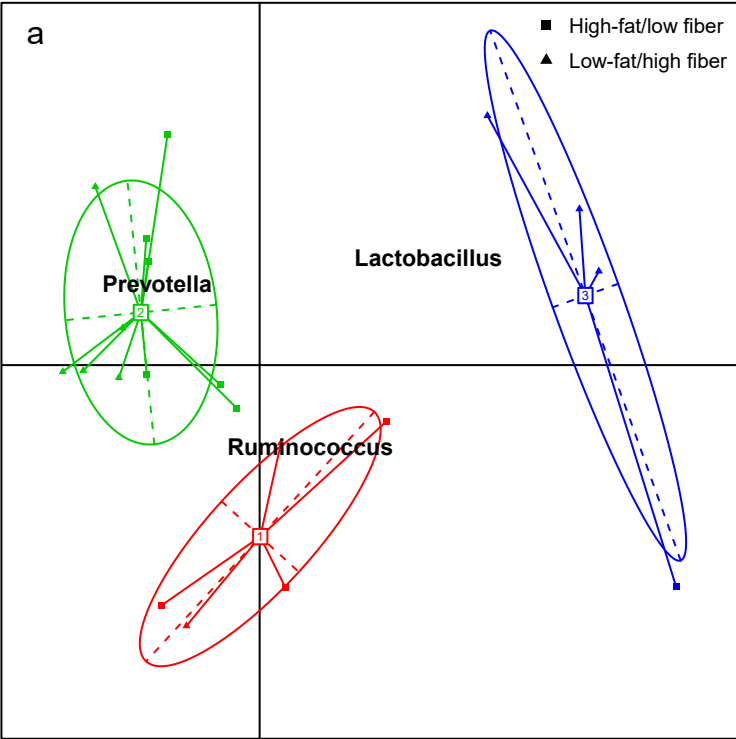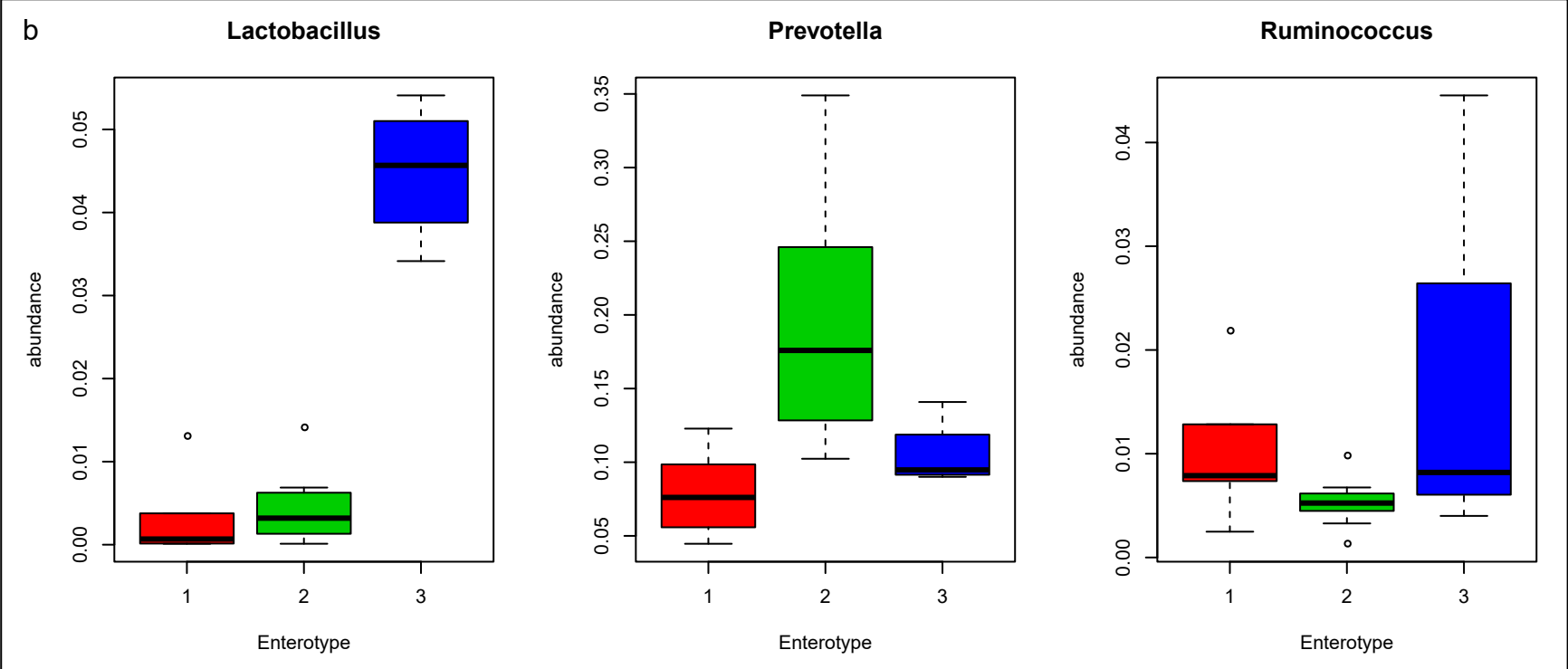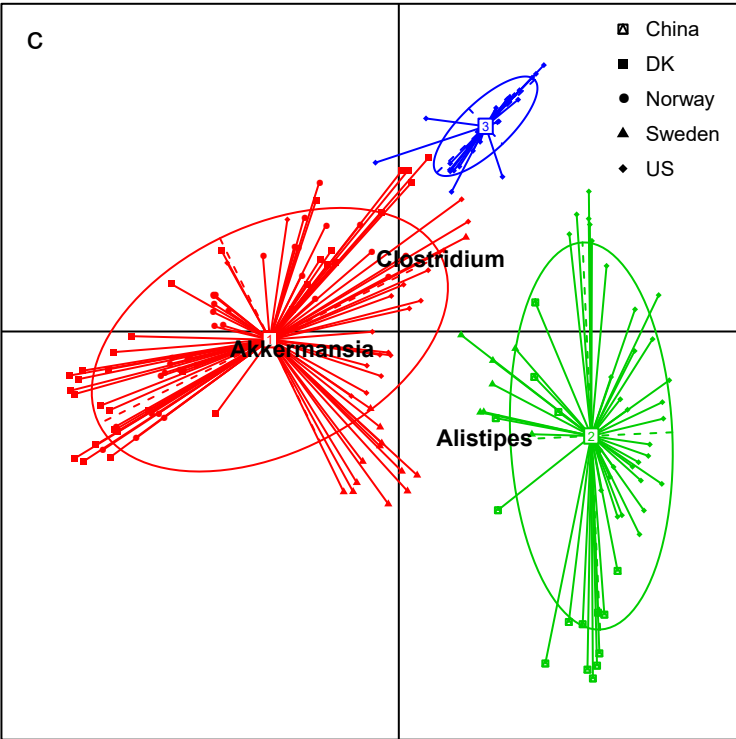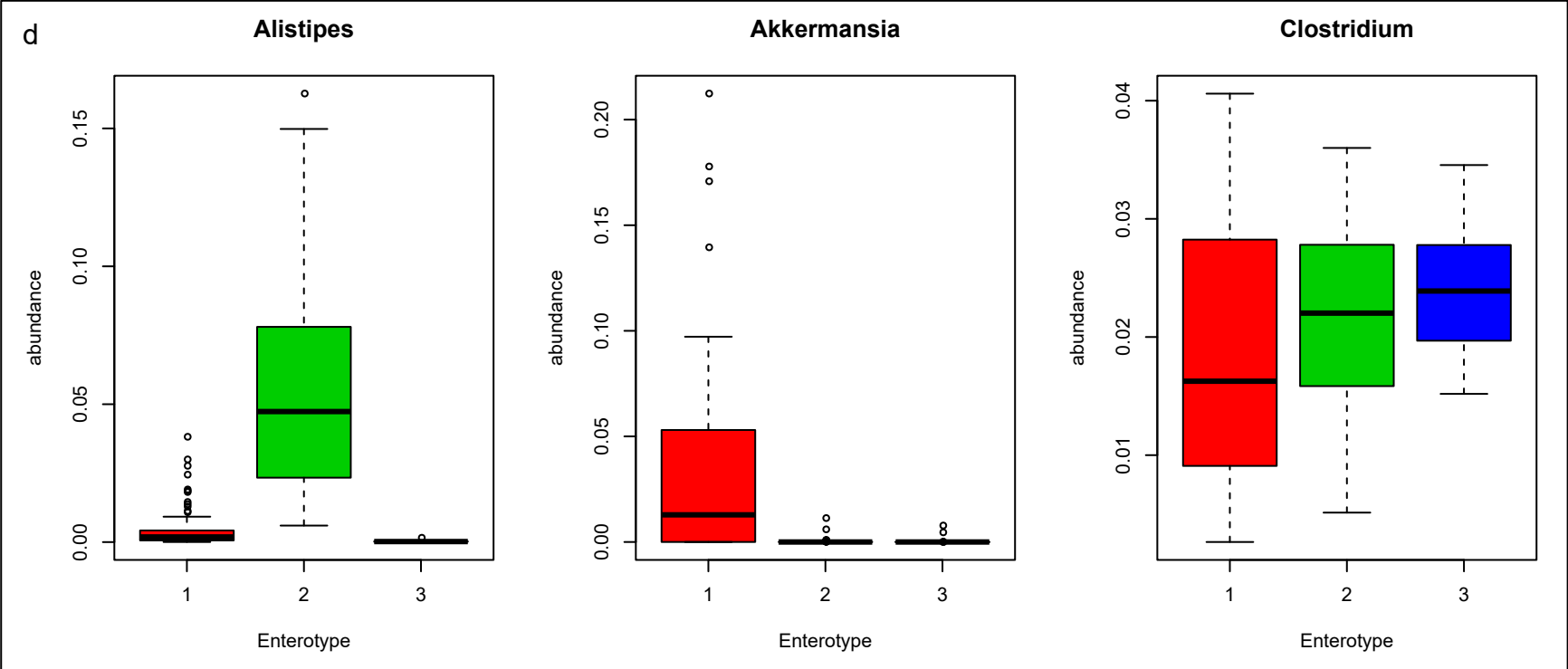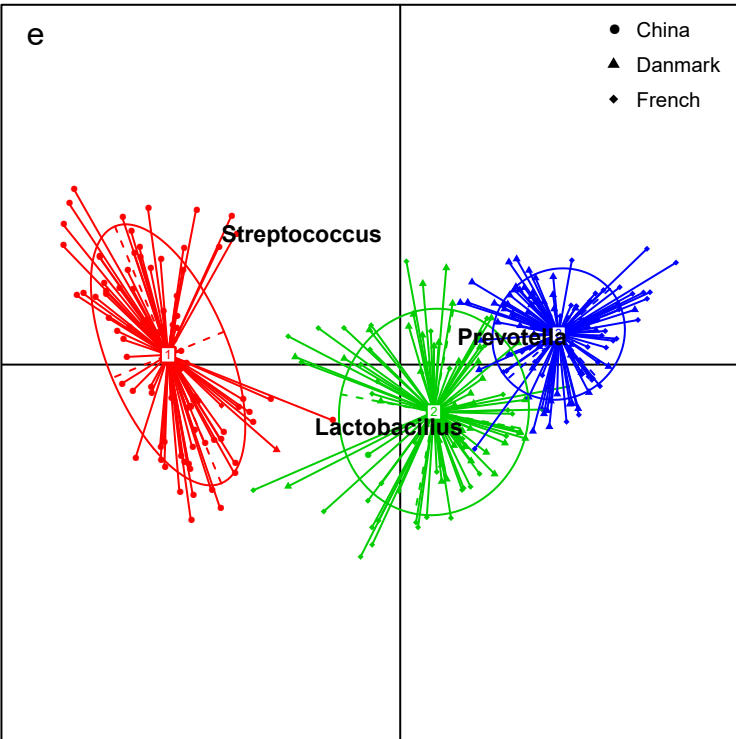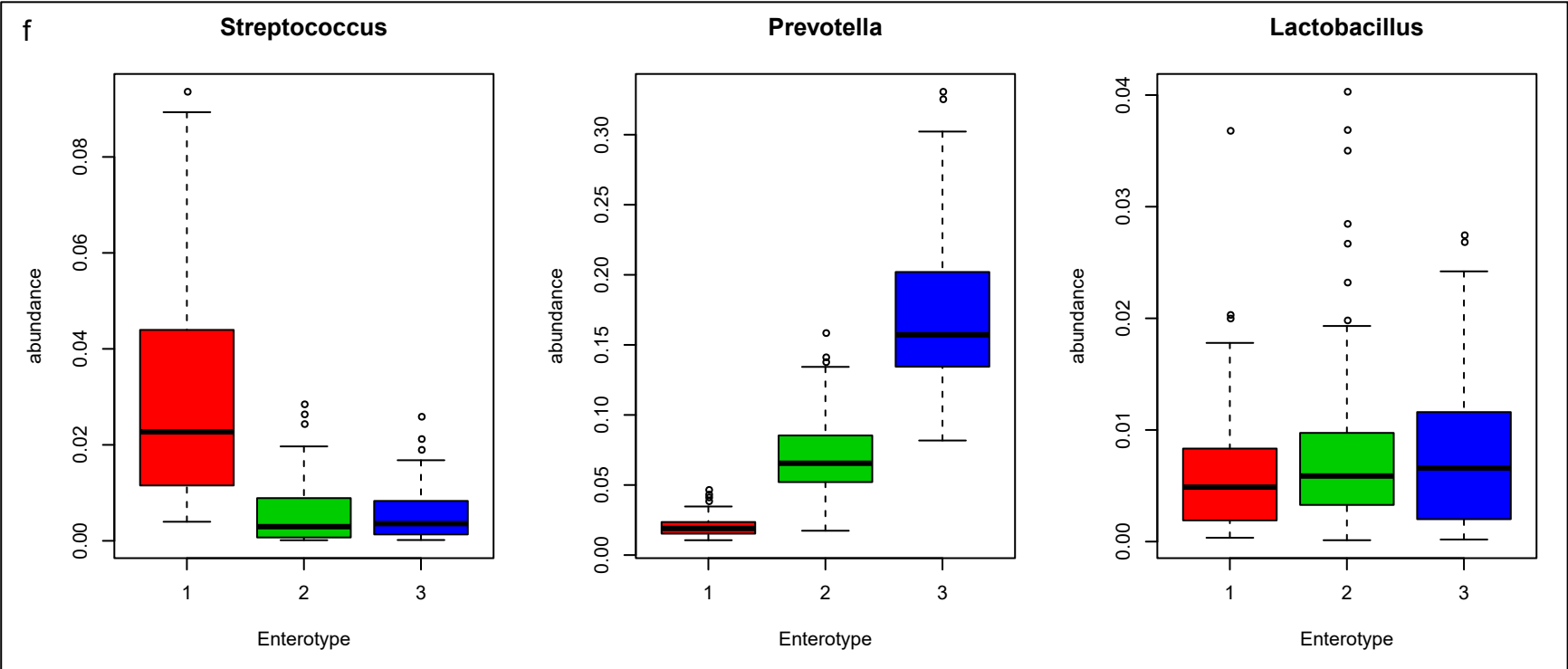

Supplement: Additional Files [file giy100_supplemental_files.zip › Additional file 4.pdf]

a

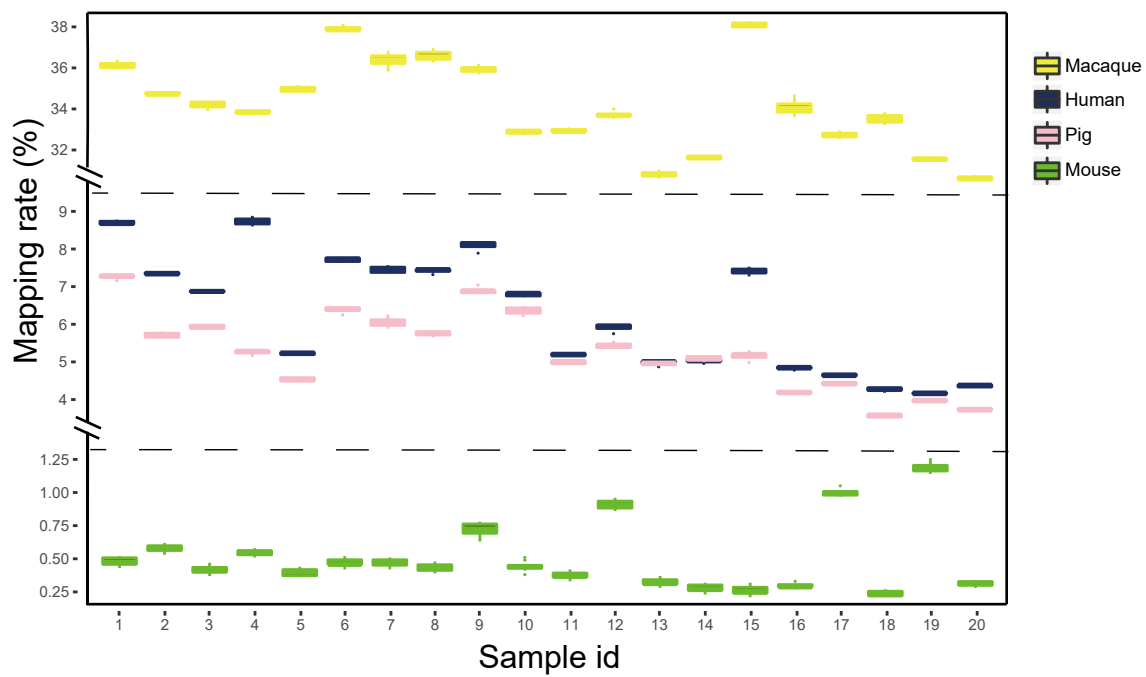

b

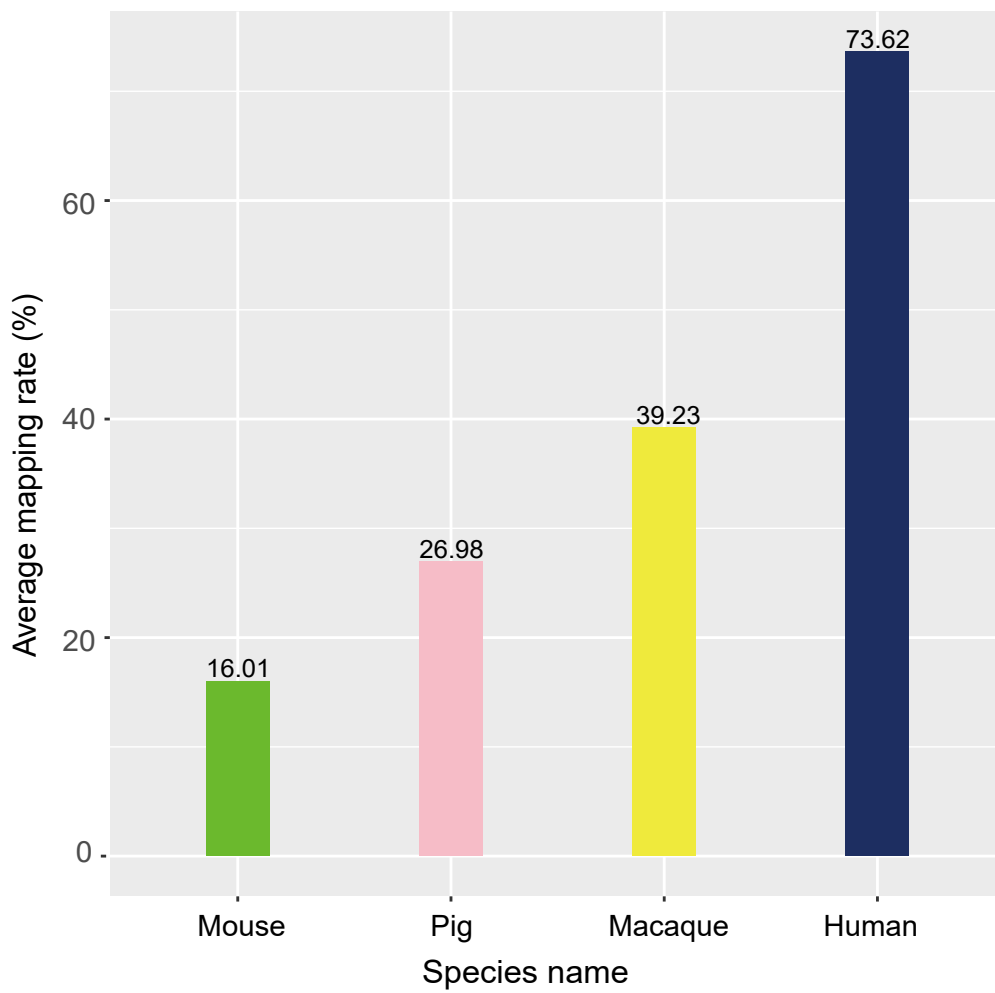

Supplement: Additional Files [file giy100_supplemental_files.zip › Additional file 6.pdf]

a

## Alpha diversity(shannon effective)

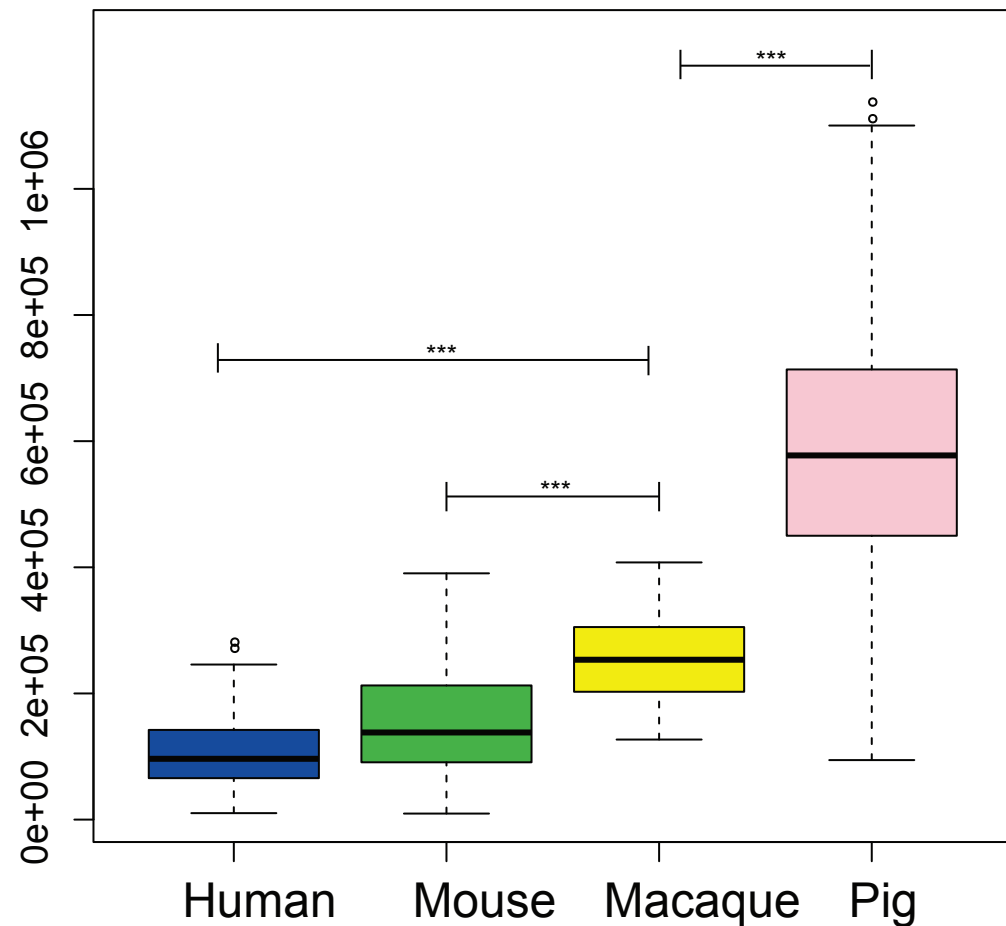

b

## Alpha diversity(shannon effective)

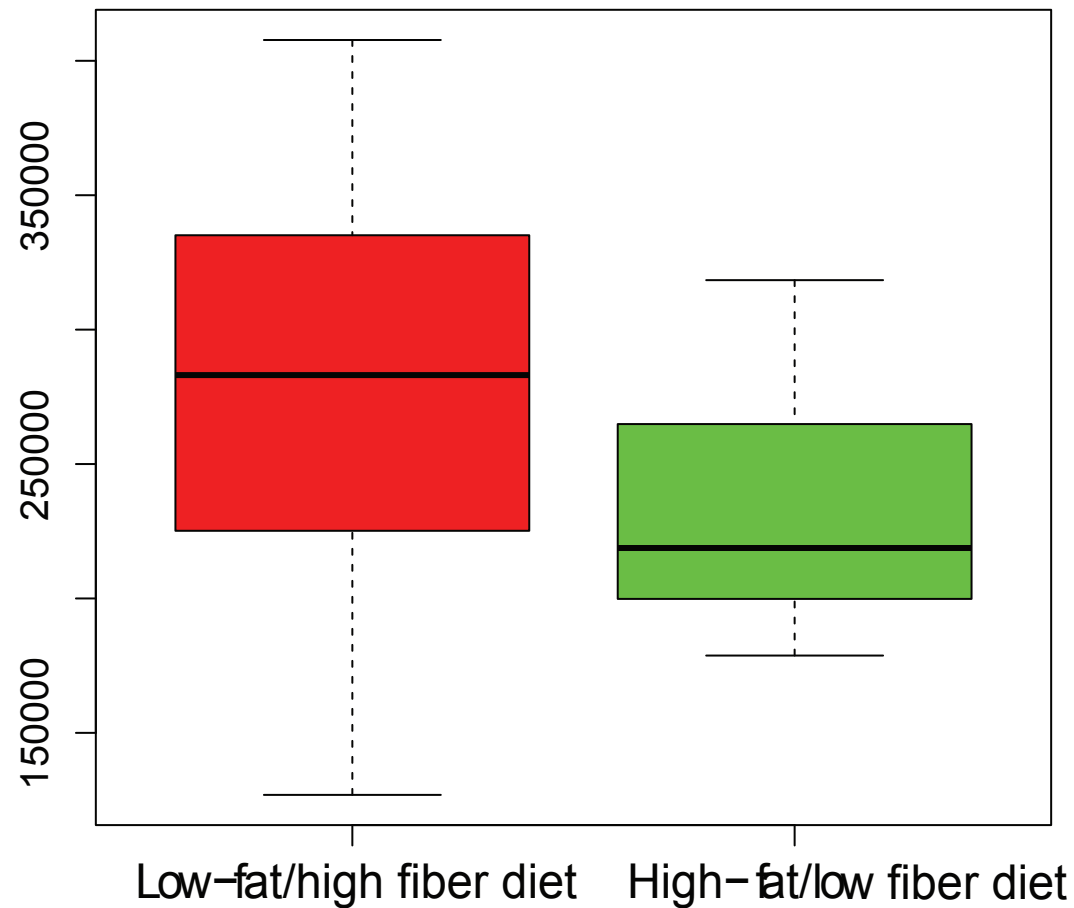

Supplement: Additional Files [file giy100_supplemental_files.zip › Additional file 7.pdf]

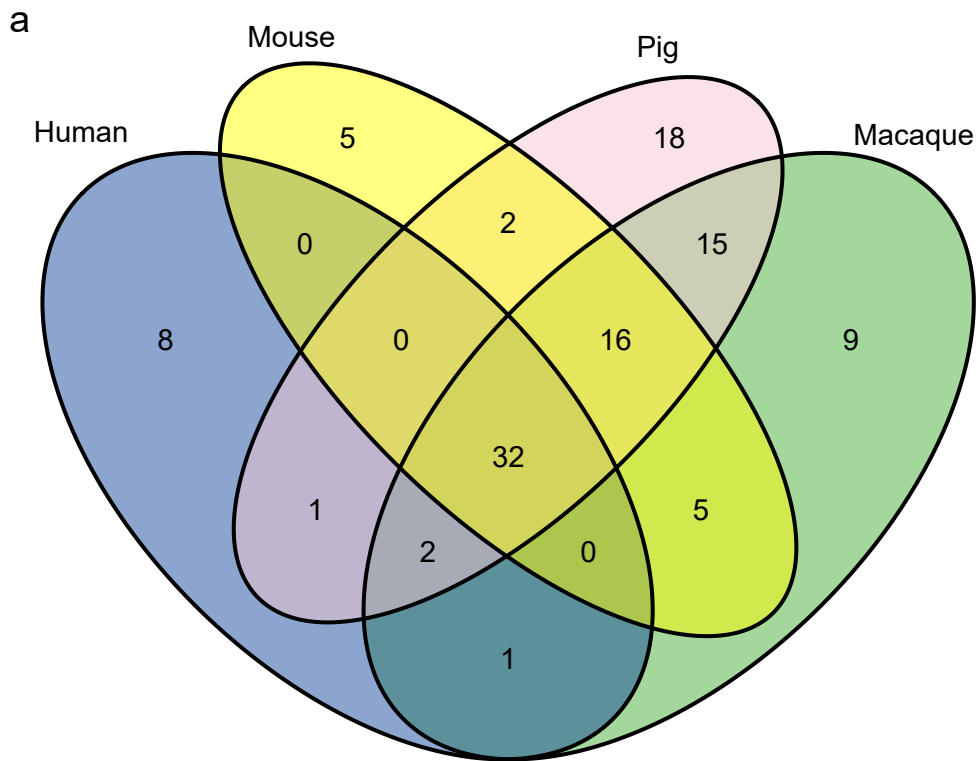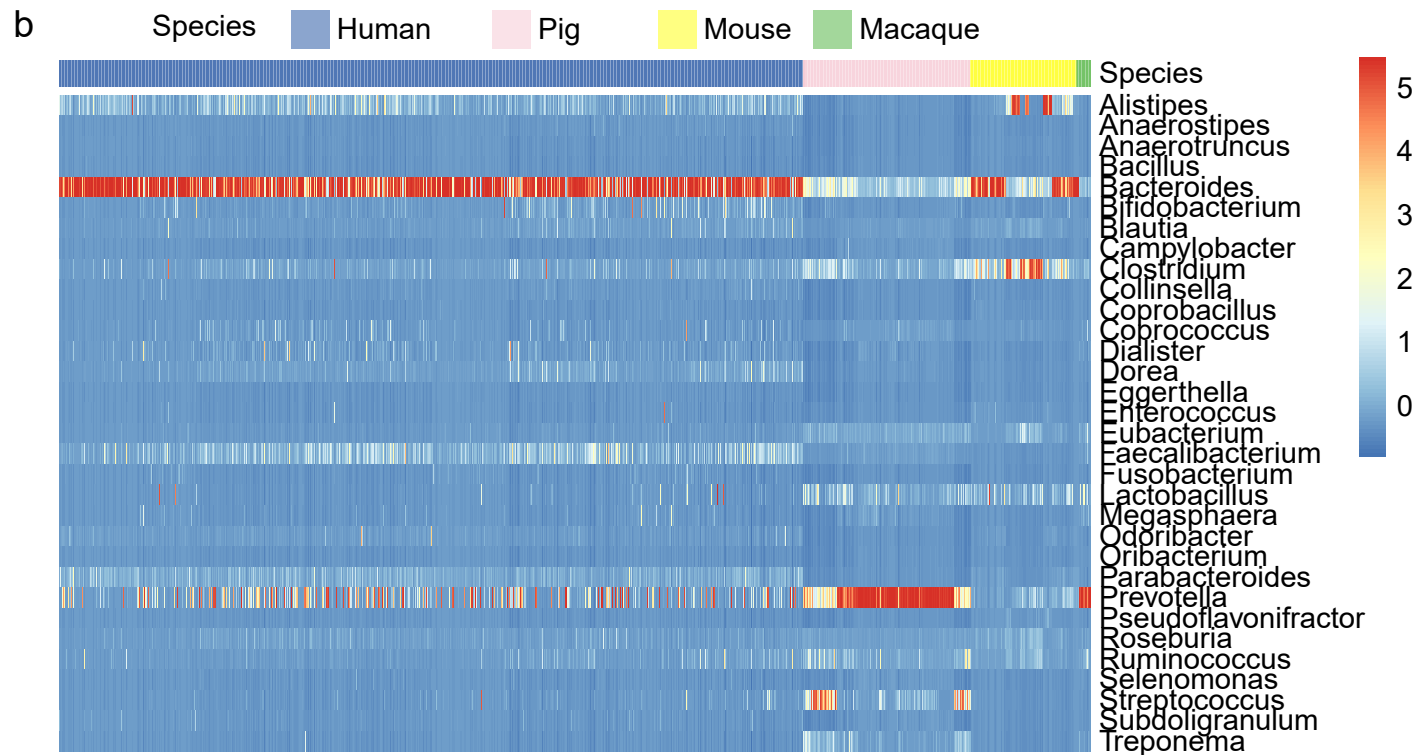

Supplement: Additional Files [file giy100_supplemental_files.zip › Additional file 8.pdf]

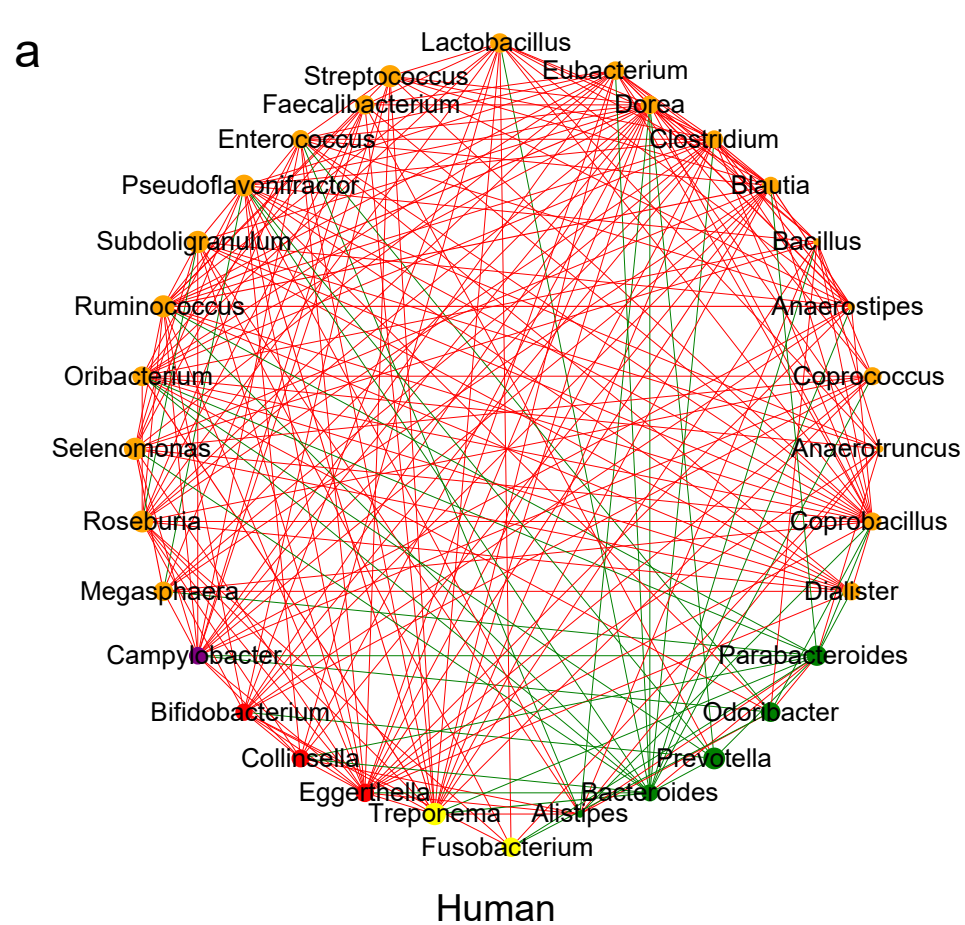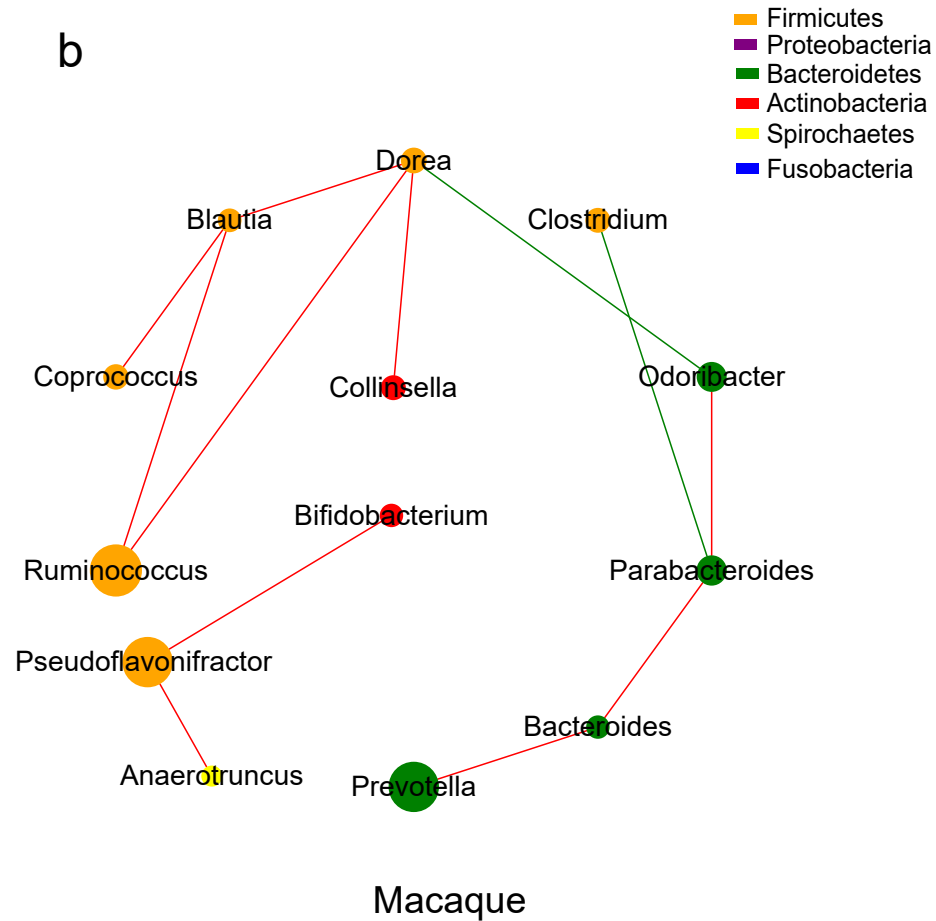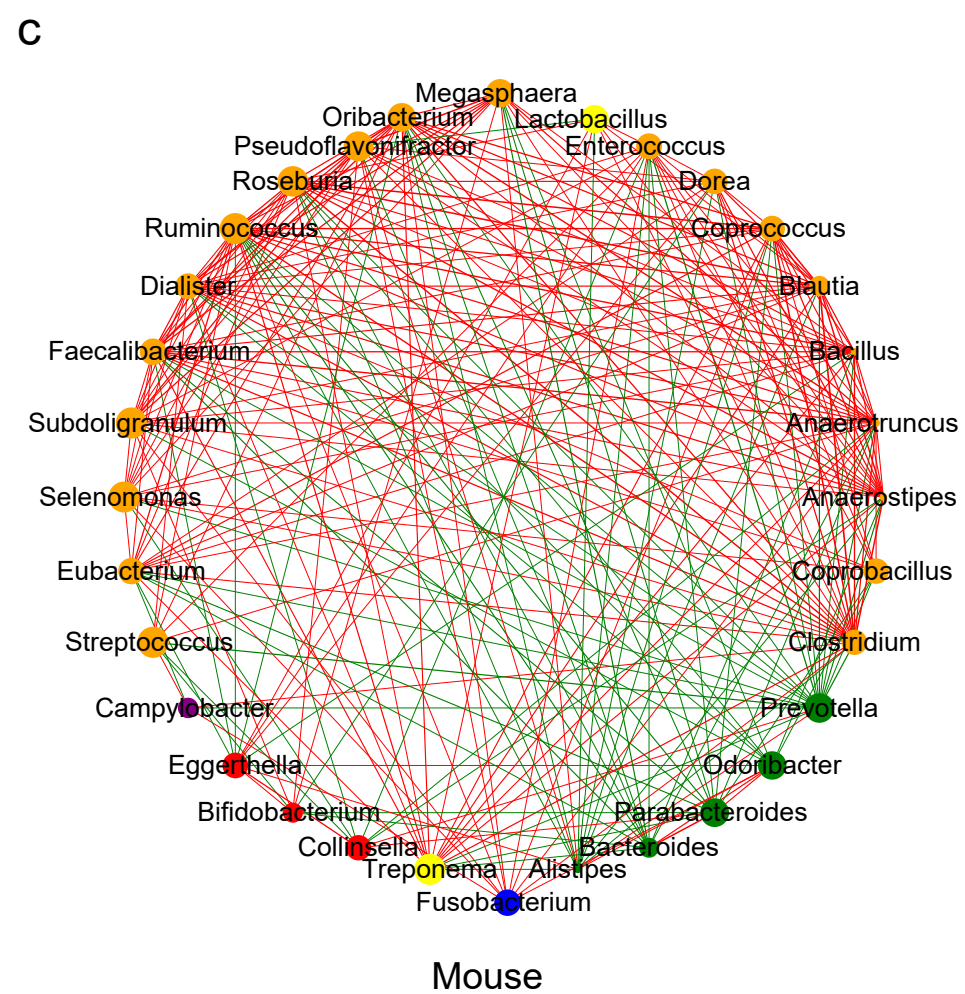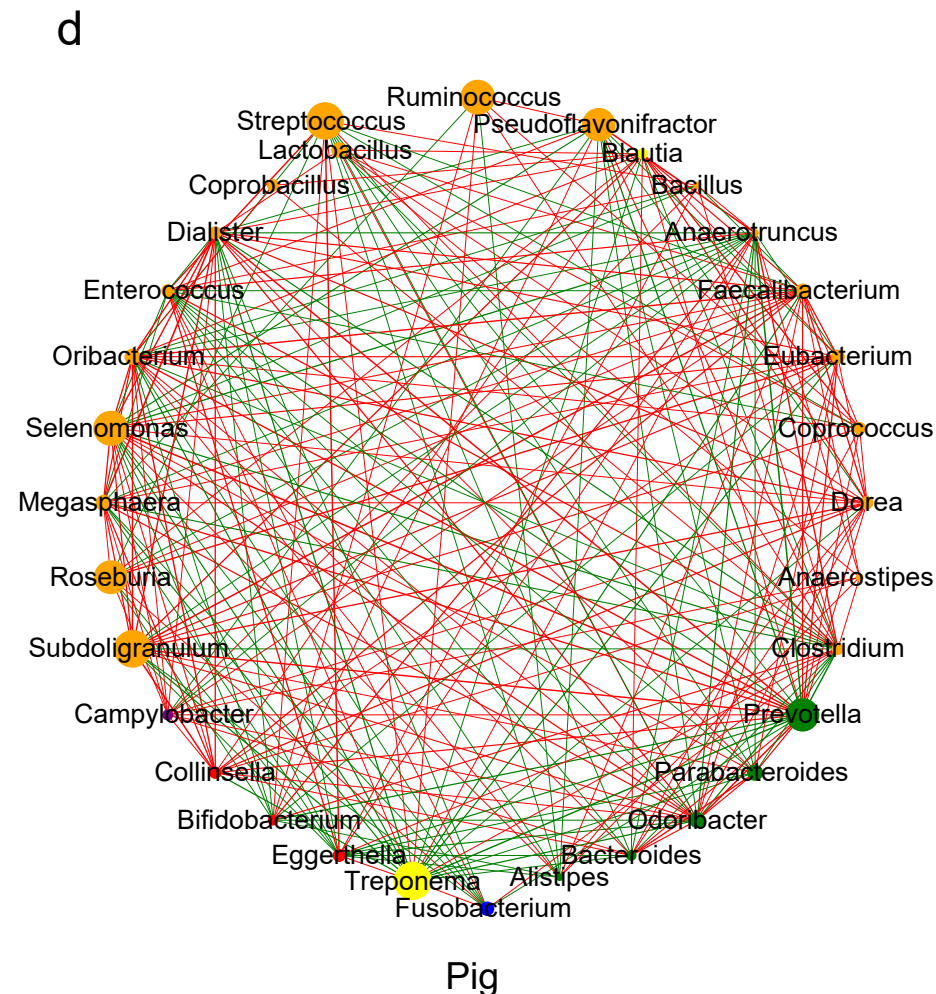

Supplement: Additional Files [file giy100_supplemental_files.zip › Additional file 9.pdf]
