# Supplementary material for: Establishment of a Macaca fascicularis gut microbiome gene catalog and comparison with the human, pig, and mouse gut microbiomes
Source: Gigascience. 2018 Aug 18;7(9):giy100. doi: 10.1093/gigascience/giy100 (PMC6137240; doi:10.1093/gigascience/giy100)
Supplement: Response_to_Reviewer_Comments_Original_Submission.pdf [file giy100_response_to_reviewer_comments_original_submission.pdf]

Author's Response To Reviewer Comments

Close

The detailed response to reviewers is in the attached file "Detailed response to reviewers".

Close
