# Supplementary material for: Establishment of a Macaca fascicularis gut microbiome gene catalog and comparison with the human, pig, and mouse gut microbiomes
Source: Gigascience. 2018 Aug 18;7(9):giy100. doi: 10.1093/gigascience/giy100 (PMC6137240; doi:10.1093/gigascience/giy100)
Supplement: Response_to_Reviewer_Comments_Revision_1.pdf [file giy100_response_to_reviewer_comments_revision_1.pdf]

Author's Response To Reviewer Comments

Close

We are very pleased by the positive comments from the reviewers and the acceptance of our manuscript for publication in GigaScience. We have made minor corrections of grammar and further adjusted the text as required by reviewer 2. See the supplemental file for a more detailed point-by-point response.

Close
