# Supplementary material for: Establishment of a Macaca fascicularis gut microbiome gene catalog and comparison with the human, pig, and mouse gut microbiomes
Source: Gigascience. 2018 Aug 18;7(9):giy100. doi: 10.1093/gigascience/giy100 (PMC6137240; doi:10.1093/gigascience/giy100)
Supplement: Reviewer_1_Report_(Original_Submission) -- Ilias Lagkouvardos, PhD — 02-01-2018 Reviewed [file giy100_reviewer_1_report_(original_submission).pdf]

Reviewer Report

Title: Establishment of a Macaca fascicularis gut microbiome gene catalog and comparison with the human, pig and mouse gut microbiomes

Version: Original Submission **Date:** 2/1/2018

Reviewer name: Ilias Lagkouvardos

Reviewer Comments to Author:

The study of Liang Xiao team, about the gene catalogue of macaques gut microbiome, is a well organized and executed project. The manuscript is clearly written and the findings well presented.
Although no groundbreaking results are shown, the gene catalogue will enhance the inferring capacity of future microbiome projects using the macaques as animal model. This is particularly important as mouse models have limitations in the translation of findings to human biology and physiology.
Despite some slightly outdated tool selections (like CARMA3) overall the technical part of the gene catalogue study is carefully and well executed.

From the other hand, the compositional analysis of the gut microbes appears to be problematic. The genera shown all have too low abundances that cumulatively count for only a very small proportion of the communities. I am afraid that this is an artifact of the taxonomic classification used. We know that a significant proportion of the gut microbes especially for human has cultivable representatives (around 80% of the total community for human, 50% for pig and from 20-80% in mouse). Therefore, I would advise to test other pipelines also, like MetaPhlAn v2 for example, to compare the results. Otherwise, claims based only on such small fraction of the microbiome cannot be expressed as descriptive of the whole communities.

Finally, as a minor note, I would also prefer Alpha diversity to be expressed as Shannon effective (linear scale) rather as Shannon index (logarithmic scale) as more intuitive and more suitable for the statistics.

Methods

Are the methods appropriate to the aims of the study, are they well described, and are necessary controls included? Yes

Conclusions

Are the conclusions adequately supported by the data shown? Yes

Reporting Standards

Does the manuscript adhere to the journal's guidelines on minimum standards of reporting? Yes

Choose an item.

Statistics

Are you able to assess all statistics in the manuscript, including the appropriateness of statistical tests used? Yes, and I have assessed the statistics in my report.

Quality of Written English

Please indicate the quality of language in the manuscript: Acceptable

Declaration of Competing Interests

Please complete a declaration of competing interests, considering the following questions:

- Have you in the past five years received reimbursements, fees, funding, or salary from an organisation that may in any way gain or lose financially from the publication of this manuscript, either now or in the future?
- Do you hold any stocks or shares in an organisation that may in any way gain or lose financially from the publication of this manuscript, either now or in the future?
- Do you hold or are you currently applying for any patents relating to the content of the manuscript?
- Have you received reimbursements, fees, funding, or salary from an organization that holds or has applied for patents relating to the content of the manuscript?
- Do you have any other financial competing interests?
- Do you have any non-financial competing interests in relation to this paper?

If you can answer no to all of the above, write 'I declare that I have no competing interests' below. If your reply is yes to any, please give details below.

I declare that I have no competing interests

I agree to the open peer review policy of the journal. I understand that my name will be included on my report to the authors and, if the manuscript is accepted for publication, my named report including any attachments I upload will be posted on the website along with the authors' responses. I agree for my report to be made available under an Open Access Creative Commons CC-BY license (http://creativecommons.org/licenses/by/4.0/). I understand that any comments which I do not wish to be included in my named report can be included as confidential comments to the editors, which will not be published.

I agree to the open peer review policy of the journal

To further support our reviewers, we have joined with Publons, where you can gain additional credit to further highlight your hard work (see: https://publons.com/journal/530/gigascience). On publication of this paper, your review will be automatically added to Publons, you can then choose whether or not to claim your Publons credit. I understand this statement.

Yes Choose an item.
