# Supplementary material for: Establishment of a Macaca fascicularis gut microbiome gene catalog and comparison with the human, pig, and mouse gut microbiomes
Source: Gigascience. 2018 Aug 18;7(9):giy100. doi: 10.1093/gigascience/giy100 (PMC6137240; doi:10.1093/gigascience/giy100)
Supplement: Reviewer_1_Report_(Revision_1) -- Ilias Lagkouvardos, PhD — 4/6/2018 Reviewed [file giy100_reviewer_1_report_(revision_1).pdf]

Reviewer Report

Title: Establishment of a Macaca fascicularis gut microbiome gene catalog and comparison with the human, pig and mouse gut microbiomes

Version: Revision 1 Date: 4/6/2018

Reviewer name: Ilias Lagkouvardos

Reviewer Comments to Author:

CARMA is indeed more appropriate for gene classification than MetaPhlAn but I did not ask for that, as this is not its intended usage. I recommended using this tool in parallel over the unassembled metagenomic reads to get a different picture of the microbial structure within your samples. In my opinion, this approach is much more reliable than CARMA. In any case, since this is mainly a gene catalogue paper I am not enforcing this and I accept the manuscript as is. As a general and friendly remark, though I would recommend you not to use the argument that a method is commonly used before to support your methodology. All methods get obsolete over time. Rather use publication that show the superiority of your method over suggested ones or make your own comparative study.

Please fix the missing space between the word preceeding the brackets of the references as xxx [1,2] rather than xxx[1,2] in several places over the manuscript.

Methods

Are the methods appropriate to the aims of the study, are they well described, and are necessary controls included? Yes

Conclusions

Are the conclusions adequately supported by the data shown? Yes

Reporting Standards

Does the manuscript adhere to the journal's guidelines on minimum standards of reporting? Yes

Choose an item.

Statistics

Are you able to assess all statistics in the manuscript, including the appropriateness of statistical tests used? Yes, and I have assessed the statistics in my report.

Quality of Written English

Please indicate the quality of language in the manuscript: Acceptable

Declaration of Competing Interests

Please complete a declaration of competing interests, considering the following questions:

- Have you in the past five years received reimbursements, fees, funding, or salary from an organisation that may in any way gain or lose financially from the publication of this manuscript, either now or in the future?
- Do you hold any stocks or shares in an organisation that may in any way gain or lose financially from the publication of this manuscript, either now or in the future?
- Do you hold or are you currently applying for any patents relating to the content of the manuscript?
- Have you received reimbursements, fees, funding, or salary from an organization that holds or has applied for patents relating to the content of the manuscript?
- Do you have any other financial competing interests?
- Do you have any non-financial competing interests in relation to this paper?

If you can answer no to all of the above, write 'I declare that I have no competing interests' below. If your reply is yes to any, please give details below.

I declare that I have no competing interests

I agree to the open peer review policy of the journal. I understand that my name will be included on my report to the authors and, if the manuscript is accepted for publication, my named report including any attachments I upload will be posted on the website along with the authors' responses. I agree for my report to be made available under an Open Access Creative Commons CC-BY license (http://creativecommons.org/licenses/by/4.0/). I understand that any comments which I do not wish to be included in my named report can be included as confidential comments to the editors, which will not be published.

I agree to the open peer review policy of the journal

To further support our reviewers, we have joined with Publons, where you can gain additional credit to further highlight your hard work (see: https://publons.com/journal/530/gigascience). On publication of this paper, your review will be automatically added to Publons, you can then choose whether or not to claim your Publons credit. I understand this statement.

Yes Choose an item.
