# Supplementary material for: Establishment of a Macaca fascicularis gut microbiome gene catalog and comparison with the human, pig, and mouse gut microbiomes
Source: Gigascience. 2018 Aug 18;7(9):giy100. doi: 10.1093/gigascience/giy100 (PMC6137240; doi:10.1093/gigascience/giy100)
Supplement: Reviewer_2_Report_(Original_Submission) -- Intawat Nookaew — 2/21/2018 Reviewed [file giy100_reviewer_2_report_(original_submission).pdf]

Reviewer Report

Title: Establishment of a Macaca fascicularis gut microbiome gene catalog and comparison with the human, pig and mouse gut microbiomes

Version: Original Submission **Date:** 2/21/2018

Reviewer name: Intawat Nookaew

Reviewer Comments to Author:

The manuscript titled "Establishment of a Macaca fascicularis gut microbiome gene catalog and comparison with the human, pig and mouse gut microbiomes" by Li et al. describe the construction of Macaca fascicularis gut microbiome gene catalogue from 20 individuals. The taxonomic profile and gene functional profile were reported and compare with pig and mouse microbiome from the previous work. The comparison between high-fat and low-fat diet microbiota of macaque was conducted. The manuscript contains many standard bioinformatics analysis on gut microbiome that difficult to follow because it is not clear detail in the methodology section. The biological interpretation is too small.

- For reproducibility and transparency of bioinformatics analyses performed in the study, I recommended the authors provide codes/commands and parameters for individual step with comments for the reader can walk through.
- Summary of gene catalogue in term of size should be provided and compared across different data set include macaque human, mice, pig. This will be the first evaluation that I strongly recommended for the authors to do.
- What is the reference taxonomic database that CAMAR3 used? This is very important to have up to date database to have a good coverage of taxonomic profile in metagenomics. The authors need to clarify on this in the manuscript.
- How to assign KO to gene. There is no clear explanation to follow.
- The authors compared the taxonomic abundance and profile among macaque, human and pig and mouse. Is the way to obtained taxonomic information of different gut microbiome the same? I believe that in the previous study of human, mouse and pig, CAMAR3 was not be employed. Therefore, taxonomic comparison across species need to be re-calculated in the exact same way for fair comparison before claiming the core bacteria genera/sepcies of mammalian gut.
- What is the massage of genera network analysis in figure 2c? Please add some context in the manuscript otherwise discard it out.
- The PCA of KO in figure 3C is plotted based on presence/absence of KO or relative abundance? The authors need to provide PCA of gene catalogue because KO cannot be assigned for all genes. How many percent of gene catalogue can be assigned KO?
- In diet-related changes study the author reported the significantly different in KO and gene. What is about taxonomic different? Please provide in the manuscript.
- How functionally enriched KO pathway was performed? There is no description in methodology.
- Analysis phage sequences was performed only in macaque. This kind of analysis should be performed and compared with human, mouse, pig gut microbiome.
- Biological interpretation of the results is need to added to improve the manuscript

Methods

Are the methods appropriate to the aims of the study, are they well described, and are necessary controls included? No

Conclusions

Are the conclusions adequately supported by the data shown? Yes

Reporting Standards

Does the manuscript adhere to the journal's guidelines on minimum standards of reporting? Yes

Choose an item.

Statistics

Are you able to assess all statistics in the manuscript, including the appropriateness of statistical tests used? Yes, and I have assessed the statistics in my report.

Quality of Written English

Please indicate the quality of language in the manuscript: Acceptable

Declaration of Competing Interests

Please complete a declaration of competing interests, considering the following questions:

- Have you in the past five years received reimbursements, fees, funding, or salary from an organisation that may in any way gain or lose financially from the publication of this manuscript, either now or in the future?
- Do you hold any stocks or shares in an organisation that may in any way gain or lose financially from the publication of this manuscript, either now or in the future?
- Do you hold or are you currently applying for any patents relating to the content of the manuscript?
- Have you received reimbursements, fees, funding, or salary from an organization that holds or has applied for patents relating to the content of the manuscript?
- Do you have any other financial competing interests?
- Do you have any non-financial competing interests in relation to this paper?

If you can answer no to all of the above, write 'I declare that I have no competing interests' below. If your reply is yes to any, please give details below.

I declare that I have no competing interests

I agree to the open peer review policy of the journal. I understand that my name will be included on my report to the authors and, if the manuscript is accepted for publication, my named report including any attachments I upload will be posted on the website along with the authors' responses. I agree for my report to be made available under an Open Access Creative Commons CC-BY license (http://creativecommons.org/licenses/by/4.0/). I understand that any comments which I do not wish to be included in my named report can be included as confidential comments to the editors, which will not be published.

I agree to the open peer review policy of the journal

To further support our reviewers, we have joined with Publons, where you can gain additional credit to further highlight your hard work (see: https://publons.com/journal/530/gigascience). On publication of this paper, your review will be automatically added to Publons, you can then choose whether or not to claim your Publons credit. I understand this statement.

Yes Choose an item.
