# Supplementary material for: Establishment of a Macaca fascicularis gut microbiome gene catalog and comparison with the human, pig, and mouse gut microbiomes
Source: Gigascience. 2018 Aug 18;7(9):giy100. doi: 10.1093/gigascience/giy100 (PMC6137240; doi:10.1093/gigascience/giy100)
Supplement: Reviewer_2_Report_(Revision_1) -- Intawat Nookaew — 4/24/2018 Reviewed [file giy100_reviewer_2_report_(revision_1).pdf]

Reviewer Report

Title: Establishment of a Macaca fascicularis gut microbiome gene catalog and comparison with the human, pig and mouse gut microbiomes

Version: Revision 1 Date: 4/24/2018

Reviewer name: Intawat Nookaew

Reviewer Comments to Author:

The author improved the manuscript. There are some comments that I would like the author to fixed as below.

- As the author said that "The main goal of our research was to establish the first macaque gut gene catalog and compare it with human, mouse and pig gut gene catalogs." I recommended the authors put the statement in the abstract and conclusion.

- Reproducibility if very important issue that community need to closely pay attention. One good article published in Giga science paper "Tools and techniques for computational reproducibility" (PMC4940747) lay a good ground to reach reproducibility in bioinformatics. The author provided some chunks of code for produce figure that a good start. However, as we known that using the same tool with different parameter set, we will get different results, leading to different biological interpretation. I STRONGLY recommend the author provide kind of simple workflow contained details of command with parameter that the author used, following the example in the published paper above. I really believe that reader include me will really appreciate and can reproduce all the results present in the manuscript.

- Author now described the way to calculate KEGG enrichment and cited the paper (ref 12) which is the original paper of reporter metabolite algorithm. Please the authors cite the reference properly as references listed below.

http://www.pnas.org/content/102/8/2685

https://bmcsystbiol.biomedcentral.com/articles/10.1186/1752-0509-2-17

Methods

Are the methods appropriate to the aims of the study, are they well described, and are necessary controls included? Choose an item.

Conclusions

Are the conclusions adequately supported by the data shown? Choose an item.

Reporting Standards

Does the manuscript adhere to the journal's guidelines on minimum standards of reporting? Choose an item.

Choose an item.

Statistics

Are you able to assess all statistics in the manuscript, including the appropriateness of statistical tests used? Choose an item.

Quality of Written English

Please indicate the quality of language in the manuscript: Choose an item.

Declaration of Competing Interests

Please complete a declaration of competing interests, considering the following questions:

- Have you in the past five years received reimbursements, fees, funding, or salary from an organisation that may in any way gain or lose financially from the publication of this manuscript, either now or in the future?
- Do you hold any stocks or shares in an organisation that may in any way gain or lose financially from the publication of this manuscript, either now or in the future?
- Do you hold or are you currently applying for any patents relating to the content of the manuscript?
- Have you received reimbursements, fees, funding, or salary from an organization that holds or has applied for patents relating to the content of the manuscript?
- Do you have any other financial competing interests?
- Do you have any non-financial competing interests in relation to this paper?

If you can answer no to all of the above, write 'I declare that I have no competing interests' below. If your reply is yes to any, please give details below.

'I declare that I have no competing interests'

I agree to the open peer review policy of the journal. I understand that my name will be included on my report to the authors and, if the manuscript is accepted for publication, my named report including any attachments I upload will be posted on the website along with the authors' responses. I agree for my report to be made available under an Open Access Creative Commons CC-BY license (http://creativecommons.org/licenses/by/4.0/). I understand that any comments which I do not wish to be included in my named report can be included as confidential comments to the editors, which will not be published.

I agree to the open peer review policy of the journal

To further support our reviewers, we have joined with Publons, where you can gain additional credit to further highlight your hard work (see: https://publons.com/journal/530/gigascience). On publication of this paper, your review will be automatically added to Publons, you can then choose whether or not to claim your Publons credit. I understand this statement.

Yes Choose an item.
